# Genome-wide association analyses identify genotype-by-environment interactions of growth traits in Simmental cattle

**DOI:** 10.1038/s41598-021-92455-x

**Published:** 2021-06-25

**Authors:** Camila U. Braz, Troy N. Rowan, Robert D. Schnabel, Jared E. Decker

**Affiliations:** 1 Division of Animal Sciences, University of Missouri, Columbia, MO 65211 USA; 2 Genetics Area Program, University of Missouri, Columbia, MO 65211 USA; 3 Informatics Institute, University of Missouri, Columbia, MO 65211 USA

**Keywords:** Agricultural genetics, Genetic association study, Genetic interaction, Genomics, Heritable quantitative trait, Quantitative trait

## Abstract

Understanding genotype-by-environment interactions (G × E) is crucial to understand environmental adaptation in mammals and improve the sustainability of agricultural production. Here, we present an extensive study investigating the interaction of genome-wide SNP markers with a vast assortment of environmental variables and searching for SNPs controlling phenotypic variance (vQTL) using a large beef cattle dataset. We showed that G × E contribute 10.1%, 3.8%, and 2.8% of the phenotypic variance of birth weight, weaning weight, and yearling weight, respectively. G × E genome-wide association analysis (GWAA) detected a large number of G × E loci affecting growth traits, which the traditional GWAA did not detect, showing that functional loci may have non-additive genetic effects regardless of differences in genotypic means. Further, variance-heterogeneity GWAA detected loci enriched with G × E effects without requiring prior knowledge of the interacting environmental factors. Functional annotation and pathway analysis of G × E genes revealed biological mechanisms by which cattle respond to changes in their environment, such as neurotransmitter activity, hypoxia-induced processes, keratinization, hormone, thermogenic and immune pathways. We unraveled the relevance and complexity of the genetic basis of G × E underlying growth traits, providing new insights into how different environmental conditions interact with specific genes influencing adaptation and productivity in beef cattle and potentially across mammals.

## Introduction

Genotype-by-environment interactions (G × E) refer to a variable response of genotypes across environments, resulting in different trait values^1,2^. G × E is a central issue in genetics, evolution, and ecology^3^, and cattle represent a unique opportunity to address these questions due to a large number of genotyped animals^4^ reared over a wide range of climatic and topographic regions. G × E may contribute to poor adaptation, which negatively affects the profitability and sustainability of the beef cattle production systems^1,5^. Studies using the relationship between breeding values and the environment have reported the existence of G × E for body weight in beef cattle^6,7^; however, limited research has focused on understanding the genetic loci of G × E underlying such traits. This knowledge could be used to better drive selection and mating decisions^8^ as well as in statistical models incorporating environmental information to accomplish more accurate genomic predictions^9^. In addition, G × E information could also be useful to predict the vulnerability of populations to climate change^3^ and to understand environmental adaptation in other mammalian species.

**Figure 1:**
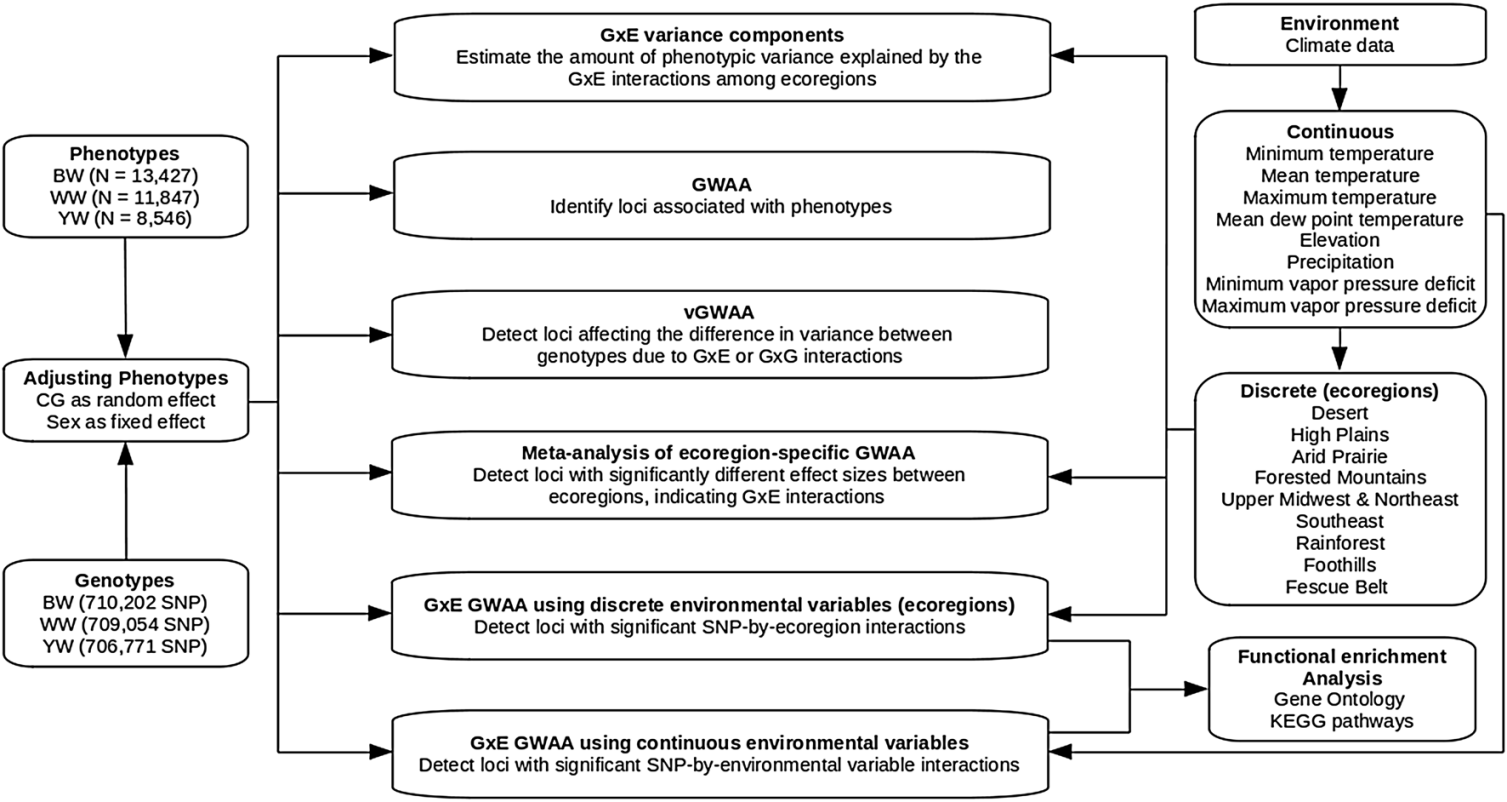
A flowchart overview of the entire study. Each topic is discussed in detail in the corresponding sections. *N *sample size, *CG *contemporary group, *BW *birth weight, *WW* weaning weight, *YW* yearling weight, *GWAA* genome-wide association analysis, *G* × *E* genotype-by-environment interactions, *vGWAA* variance-heterogeneity GWAA.

Here, we present a comprehensive study of G × E for birth weight (BW), weaning weight (WW), and yearling weight (YW) using SNP marker information and over 13,000 Simmental cattle. First, we estimated the contribution of G × E variance on the total phenotypic variances using a restricted maximum likelihood (REML) approach. Then, we searched for SNPs enriched for G × E using four linear mixed model approaches: (i) direct G × E genome-wide association analyses (G × E GWAA) using continuous environmental variables; (ii) direct G × E GWAA using discrete environmental variables (United States ecoregions); (iii) variance-heterogeneity genome-wide association analyses (vGWAA), which enable identification of interactions, using phenotypes adjusted for additive relationship matrix or additive, dominance and epistatic relationship matrices; and (iv) meta-analysis of ecoregion-specific GWAA. Lastly, we performed enrichment analyses using G × E candidate genes to investigate the biological mechanisms by which environmental factors modulate such phenotypes. A flowchart overview of this study is shown in Fig. 1. To our knowledge, this is the most comprehensive study investigating the interactions of genome-wide SNP markers with a vast assortment of environmental variables and searching for SNPs controlling phenotypic variance using a large beef cattle dataset. Our results revealed novel trait associations and alternative biological mechanisms involved in shaping the total phenotypic variance of growth traits providing new insights into how the environment influences growth traits and adaptation in beef cattle and potentially across mammals.

## Results

### Environmental variance component estimates

Phenotypes were pre-adjusted for fixed effect of sex and random effect of contemporary groups (combination of farm, season and year of birth), which represent differences in management, to remove any confounding between environment and management effects. The amount of BW, WW, and YW variation explained by contemporary group effects was 19%, 53%, and 61%, respectively, showing that the effect of farm management on the animal body weight increases with age. After adjusting the phenotypes, we estimated the contribution of G × E variance on the total phenotypic variances using additive relationship matrix calculated based on SNP marker information, and United States ecoregions as environmental factor. Ecoregions were determined based on the combination of 30-year normal values of mean temperature, precipitation, and elevation^10^. Each 1 km square of the United States was then assigned to one of 9 resulting ecoregions. We found that G × E variances contribute in 10.1%, 3.8%, and 2.8% to the BW, WW, and YW total phenotypic variances, respectively, suggesting that there is a decrease in the effect of G × E on the animal body weight throughout life. These results indicate an inverse relationship between farm management and environmental stress and that management practices (e.g. season of birth, forage quality and quantity, feed supplementation, health programs) are more relevant for later weights than climate environmental factors. One possible explanation for these findings is that birth weight reflects intrauterine growth which is highly affected by environmental conditions^11^; however, as the animal ages physiological, morphological, endocrine, cellular and molecular mechanisms are altered in order to cope up with environmental challenges^12^. These results also suggest that as management inputs are decreased, animals become more sensitive to environmental stress, which has ramifications for animal performance as we seek to decrease inputs to make beef production more sustainable.

### Genotype-by-environment GWAA

**Figure 2:**
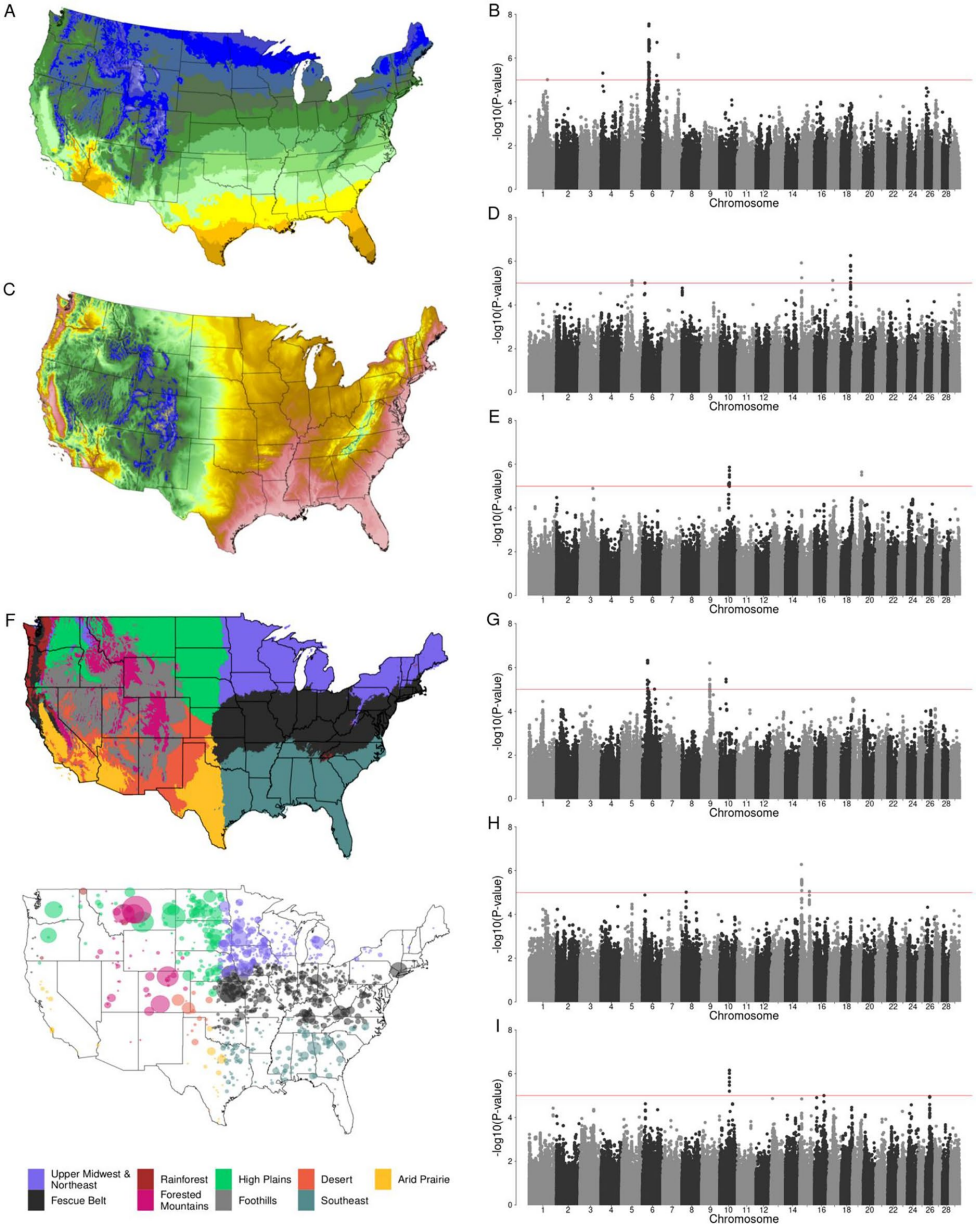
Genotype-by-environment interaction genome-wide association analyses (G × E GWAA) for growth traits in Simmental cattle. (**a**) Mean temperature average annual over the most recent three full decades covering the conterminous United States. (**b**) Manhattan plot of G × E GWAA of mean temperature for birth weight. (**c**) Elevation of the conterminous United States. (**d**) Manhattan plot of G × E GWAA of elevation for weaning weight. (**e**) Manhattan plot of G × E GWAA of elevation for yearling weight. (**f**) Boundaries for ecoregion assignments in the United States; (top panel) United States partitioned into nine ecoregions based on similar topographic and environmental conditions; (bottom panel) location of beef farms for which data was retrieved. (**g**) Manhattan plot of G × E GWAA of Forested Mountains ecoregion for birth weight. (**h**) Manhattan plot of G × E GWAA of Forested Mountains ecoregion for weaning weight. (**i**) Manhattan plot of G × E GWAA of Forested Mountains ecoregion for yearling weight. In Manhattan plots, horizontal red line indicates a significant threshold (*P* < 1e−5). Environmental continuous variables were drawn from the PRISM climate dataset (http://prism.oregonstate.edu). The United States was partitioned into nine regions using k-means clustering. Maps were plotted using the maps R package (version 3.1, https://cran.r-project.org/web/packages/maps/), using public domain data from the US Department of Commerce, Census Bureau.

The G × E GWAA for BW, WW, and YW were performed by fitting univariate and multivariate linear mixed models considering continuous or discrete environmental variables. The continuous environmental variables used were minimum, mean, maximum, and mean dew point temperatures; elevation; precipitation; and minimum and maximum vapor pressure deficit, separately (Additional file 1: Figures S1–S8). Discrete environmental variables included United States ecoregions (Desert and Arid Prairie, Southeast, High Plains, Forested Mountains, Fescue Belt, and Upper Midwest and Northeast—Fig. 2f), which are combinations of mean temperature, precipitation, and elevation 30-year normal values (Additional file 1: Figures S9–S14). Descriptive information for growth traits and environmental variables are included in Additional file 2: Table S1.

**Figure 3:**
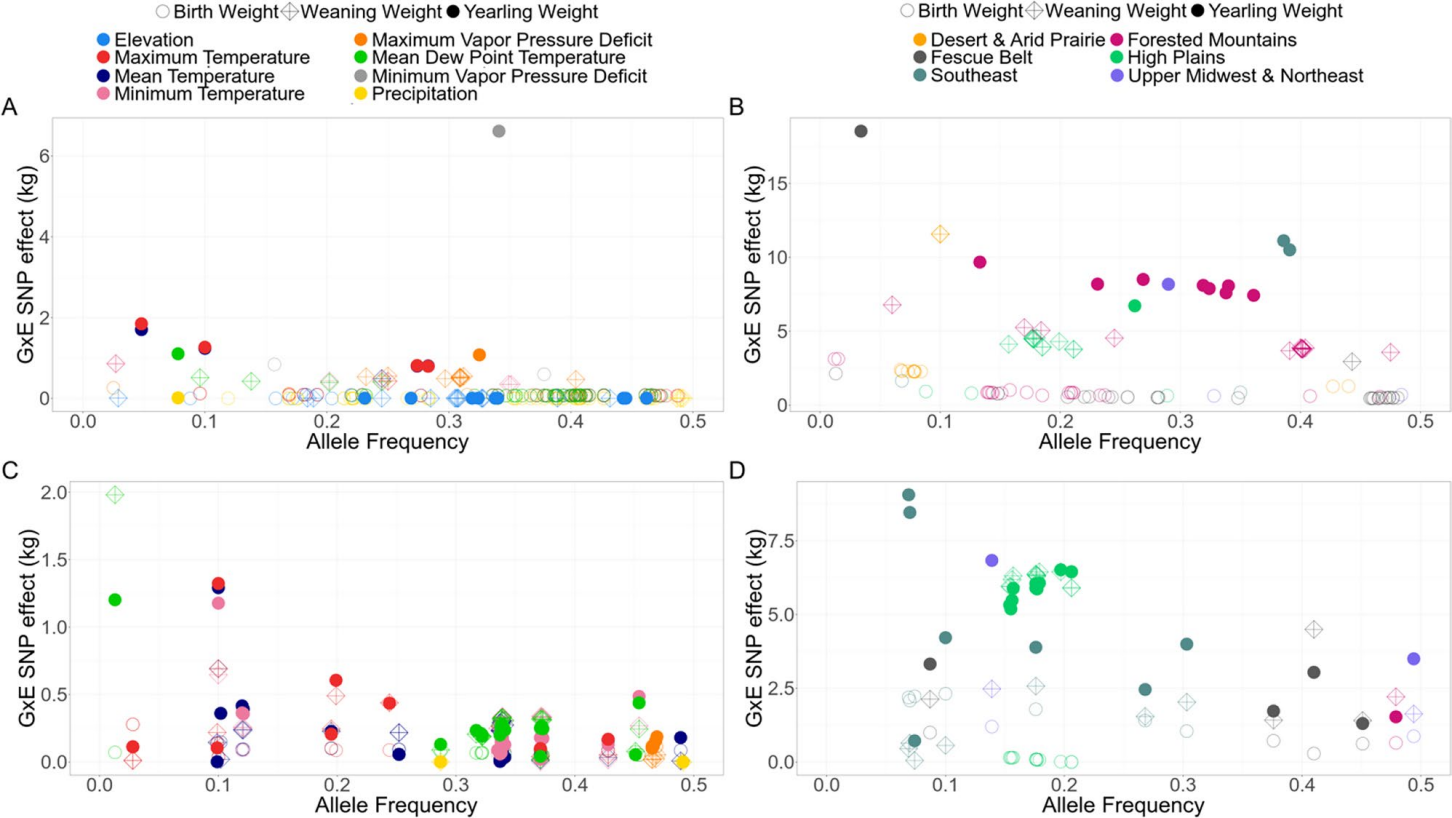
The absolute values of the significant G × E SNP effects and their allele frequency using (**a**) univariate models with continuous environmental variables, (**b**) univariate models with ecoregion, (**c**) multivariate models with continuous environmental variables, and (**d**) multivariate models with ecoregion. Figure made with R version 3.6.3. (https://www.r-project.org/).

Univariate and multivariate models using both continuous and ecoregions environmental variables detected a total of 2319 G × E SNPs (*P* < 1e−5, Table 1, Additional files 3 and 4). However, G × E GWAA can be affected by population environmental substructure (in addition to population genetic substructure), which may cause spurious signals^13^, therefore for such analyses the *P*-values were adjusted using the genomic control inflation factor (Additional file 2, Table S2). After this adjustment, 614 G × E SNPs remained significant (*P* < 1e−5, Table 1, Additional files 3 and 4), not all of which are independent. Figure 3 shows the absolute values of the significant G × E SNP effects and their allele frequency. The majority of the G × E SNP had small effects on the traits; however, large G × E SNP effects were also detected, such as the *rs109164178* (chromosome 2: 81,187,988 bp) with effect of 6.6 kg on YW when interacting with minimum vapor pressure deficit (Fig. 3a); the *rs110995777* (chromosome 13: 16,232,193 bp) with effect of 18.5 kg on YW when comparing animals reared in the Fescue Belt ecoregion and the total dataset (Fig. 3b), among others. The multivariate model also detected G × E SNP with large effects, such as *rs42917868* (chromosome 14:52,925,690 bp) when interacting with mean dew point temperature has an effect of 0.07 kg, 2 kg, and 1.2 kg on BW, WW, and YW, respectively (Fig. 3c); and *rs385339402* (chromosome 16: 49,283,484 bp) with G × E effect of 2.2 kg, 0.46 kg, and 9.1 kg on BW, WW, and YW, respectively, when comparing animals from the Southeast ecoregion and the total dataset (Fig. 3d).

**Table 1:** Results of the G × E GWAA before and after adjustment for genomic control using continuous environmental variables or United Stated ecoregions as environmental factors in univariate and multivariate analysis for birth weight, weaning weight, and yearling weight.

Env	Birth weight			Weaning weight			Yearling weight			Multivariate		
	N_B_	N_A_	QTL_A_	N_B_	N_A_	QTL_A_	N_B_	N_A_	QTL_A_	N_B_	N_A_	QTL_A_
Elev	142	5	5	28	15	7	15	10	3	50	–	–
Precip	338	77	11	4	4	1	1	1	1	12	2	2
MTemp	555	55	18	1	1	1	4	4	4	69	24	10
MinTemp	756	55	14	6	6	4	–	–	–	88	28	6
MaxTemp	408	49	18	1	1	1	4	4	4	23	9	8
MDpTemp	941	63	15	4	4	3	1	1	1	94	25	7
MinVPD	152	2	2	2	–	–	1	1	1	10	–	–
MaxVPD	224	5	4	10	10	4	1	1	1	17	6	1
SE	54	2	2	–	–	–	2	2	1	7	7	5
HP	40	3	3	16	12	2	4	1	1	23	13	1
FM	90	21	9	31	17	5	49	8	1	148	1	1
FB	47	32	9	1	1	1	1	1	1	11	4	4
UN	24	2	2	–	–	–	1	1	1	10	2	2
DA	125	13	3	8	3	1	6	–	–	*	*	*
Total	1923	384	115	101	74	30	78	35	20	353	121	47

*Env *environmental variables, *N*_*B*_ number of significant SNPs prior to genomic control adjustment, *N*_*A*_ number of significant SNPs after genomic control adjustment, *QTL*_*A*_ number of significant G × E QTL based on haplotype blocks of the significant G × E SNP after adjustment for genomic control, *Elev* elevation, *Precip* precipitation, *MTemp* mean temperature, *MinTemp* minimum temperature, *MaxTemp* maximum temperature, *MDpTemp* mean dew point temperature, *MinVPD* minimum vapor pressure deficit, *MaxVPD* maximum vapor pressure deficit, *SE* Southeast, *HP* High Plains, *FM* Forested Mountains, *FB* Fescue Belt, *UN* Upper Midwest and Northeast, *DA* Desert and Arid Prairie. The complete description of the G × E SNPs can be found in the Additional files 3 and 4. *Multivariate G × E GWAA was not performed for DA due to small sample size.

G × E SNPs were clustered into haplotype blocks based on their linkage disequilibrium, which are referred to as G × E QTL hereafter (Table 1). In total, 212 independent G × E QTL were detected. Few G × E QTL detected by the ecoregion G × E GWAA were also identified by the continuous G × E GWAA. These results indicate that the use of ecoregion information as environmental variable allowed us to capture G × E QTL interacting with region-specific stressors that were not directly measured (e.g. forage differences, water availability, pathogens) and the combination of multiple stressors. There was no overlap between the G × E QTL identified across the traits using univariate models, however, multivariate models detected several G × E QTL associated with BW, WW, or YW (*P* < 1e−5, Table 1). In addition, multivariate analyses were able to detect G × E QTL that were not identifiable by standard univariate analyses, indicating advantages of incorporating a multivariate statistical approach to G × E genome-wide association studies of correlated traits.

Consistent with our G × E variance component results, a larger number of G × E QTL was detected for BW than for WW or YW (Table 1). Temperature showed more interactions with QTL affecting BW than the other environmental variables analyzed (*P* < 1e−5, Fig. 2a,b). The ecoregions that most interacted with QTL for BW were Fescue Belt and Forested Mountains (*P* < 1e−5, Fig. 2g). For WW and YW, elevation (Fig. 2c) was found to be the continuous environmental variable that most interacted with genotype (*P* < 1e−5, Fig. 2d,e respectively). In agreement, Forested Mountains, the ecoregion with the highest elevation (Additional file 2: Table S1), showed more genotype-by-environment interaction for WW and YW (P < 1e−5, Fig. 2h,i*,* respectively), compared with other ecoregions. No common G × E QTL was identified for two or more different ecoregions indicating that the loci interacting with abiotic and biotic stressors are different across divergent environments, and consequently, the ecoregions have specific genetic architecture involved in G × E.

We explored genomic regions that flanked all significant G × E QTL (*P* < 1e−5) to scan for genes in their vicinity and to identify possible regulatory elements (Additional file 5). Using these G × E candidate gene sets, we performed functional enrichment analysis (FDR ≤ 10%) to get insights into biological mechanisms by which beef cattle respond to changes in their environment (Additional file 2, Table S3). Enriched GO terms revealed molecular functions of G × E genes that are involved in neurotransmission processes such as transmitter-gated channel activity, ligand-gated channel activity, ionotropic glutamate receptor activity, GABA-A receptor activity, neurotransmitter receptor activity involved in regulation of postsynaptic membrane potential, neurotransmitter receptor activity, among others. Function analysis also unveiled KEGG pathways involved in endocrine system (estrogen signaling pathway, and steroid hormone biosynthesis), immune response (*Staphylococcus aureus* infection, and viral myocarditis), metabolic processes (metabolism of xenobiotics by cytochrome P450, ascorbate and aldarate metabolism, pentose and glucuronate interconversions, and retinol metabolism), signal transduction (phospholipase D signaling pathway, and VEGF signaling pathway), cellular processes (endocytosis), and nervous system activity (glutamatergic synapse). Reactome pathways such as formation of the cornified envelope and keratinization were also enriched. In addition, many G × E genes participate in nitrogen compound metabolic process, response to stimulus, protein metabolic process, developmental process, anatomical structure development, gene expression, nervous system development, immune system, among other biological processes (Additional file 2, Table S4).

The majority of G × E SNP associations were identified for BW in this study. Studies have shown that loci identified in GWAA of BW could either represent direct effects of the fetal genotype, indirect effects of maternal genotype (acting via the intrauterine environment), or some combination of the two^14,15^. According to Sharma et al.^16^ the uterine environment may even have a greater influence on fetal growth and BW than the parental genome. Thus, we investigated whether G × E SNPs affecting BW could be reflecting maternal uterine environmental conditions. To test this, we adjusted BW by including random maternal effects in the model (MAM) and compared the results with those from the G × E GWAA without maternal effects (MA). Results showed that 876 SNPs were not associated with G × E effects when accounting for the effect of dam. After adjustment for genomic control, 94 G × E SNP were not detected when accounting for the effect of dam (Additional file 6), suggesting that those SNPs are interacting with the uterine environment. Several maternally-influenced G × E genes identified in this study have been previously reported as maternal genes influencing embryonic development and/or response to environmental stress in cows and also in other mammals^17–26^. These results demonstrate that the bovine is an important model to understand the interaction between the physical environment, uterus, and fetus, and could be used in future research.

### Variance-heterogeneity GWAA

The vGWAA identify loci associated with differences in variance, rather than differences in mean, between genotype classes, called vQTL, which can be explained by G × E or G × G (epistatic) interactions^27^. Therefore, we performed a REML analysis to estimate how much of the BW, WW, and YW variance is explained by additive, dominance and epistasis effects. We fitted models (M) using only additive relationship matrix (MA) or using additive, dominance, and epistasis relationship matrices (MADE), see Additional file 2: Table S5. Overall, the heritabilities were greater than the ratio of the variance explained by the non-additive genetic effects to the phenotypic variances. Proportions of dominance variances in WW and YW were low (2% and 5%, respectively), whereas no dominance variance was estimated for BW. Epistatic relationship matrix accounted for 25%, 12% and 11% of the phenotypic variances for BW, WW, and YW, respectively, suggesting that epistasis has a substantial contribution to phenotypic variation for body weight traits in Simmental cattle. Thus, to search for vQTL we used squared normalized residuals (z^2^), which are a measure of phenotypic variation^28^, as dependent variables fitting univariate and multivariate models. Using z^2^ from the MA, we expected to detect loci with both G × E and G × ﻿G effects, since this phenotype was adjusted only for the additive relationship matrix. However, using z^2^ from the MADE, we should detect exclusively G × E loci as the phenotype was adjusted for additive, dominance and epistatic relationship matrices. In total, 44 SNPs with effect on z^2^ (*P* < 1e−5) were identified on chromosomes 3, 5, 8, 10, 11, 13, 14, 16, 17, 18, 21, 27, and 29 (Additional file 1: Figures S15 and S16, Additional file 2: Table S5). These 44 SNPs tag 24 putative vQTL (grouped by LD patterns), with seven, four, and eleven vQTL for univariate BW, WW, and YW, respectively. The multivariate analysis identified five vQTL, of which two vQTL (*rs109142386*, chromosome 8:4,822,842 bp; and *rs135379559*, chromosome 29:39,441,489 bp) were not identified by the univariate analyses.

Nine vQTL were only significant using z^2^ from MA (three vQTL for univariate BW, three vQTL for univariate WW, two vQTL for univariate YW, and one vQTL using multivariate analysis). Among these vQTL likely involved in G × ﻿G, one vQTL for BW is located on chromosome 14 at 23 Mb and resides near or within the genes *LYN, RPS20, MOS, PLAG1,* and *CHCHD7*. This region has been reported to have epistatic effects on several chromosomes affecting post-weaning weight in beef cattle^29^. These effects could be due to the gene *PLAG1* which encodes a transcription factor that regulates the expression of several genes involved in a variety of cellular processes^30^, and has been previously associated with stature and growth traits in many beef cattle breeds^31–33^. In addition, we detected a G × ﻿G vQTL on chromosome 3 at 106 Mb within *TRIT1*, a gene that was also reported to harbor a vQTL involved in epistatic interactions in humans^34^.

Using z^2^ from MADE, 15 vQTL were detected (four vQTL for univariate BW; one vQTL for univariate WW; nine vQTL for univariate YW; and four vQTL using multivariate analyses, of which three vQTL were also identified by the univariate analyses), which likely identify G × E. Based on GO information, genes near or within these vQTL are involved in response to stimulus (*NOS1, FBLN1, YWHAH,* and *DEPDC5*), inflammation and immunity mechanisms (*LYZL1, KSR2, FBXO21, IL18RAP, XCL2, XCL1, TTYH1, LENG8, LENG9, CDC42EP5, LAIR1, OSCAR,* and *TARM1*), neurogenesis (*ZHX2,* and *NOS1*), regulation of transcription (*ZHX2, YWHAH*, and *CNOT3*), developmental process (*FBXW8,* and *CNOT3*), transmembrane transport (*SLC9A2, SLC9A4, SLC5A1, SLC5A4,* and *TTYH1*), neurotransmission regulation (*SHANK2*), and cell proliferation (*DPT, RPS9,* and *TACC1*). Some of those genes were previously reported to be involved in environmental adaptation in cattle (*XCL2*^35^, *SLC9A4*^36^, *ZMAT4*^37^), and heat tolerance in buffalo (*XCL1*^38^) and dairy cattle (*SHANK2*^39^).

Using z^2^ as dependent variables in the analyses allowed the estimation of the proportion of residual variance that is explained by the SNPs in GEMMA (PVE). For BW z^2^, WW z^2^ and YW z^2^, SNPs explained 4.9% (SD = 0.8), 4.7% (SD = 0.8), and 5.9% (SD = 1.1), respectively, showing that residual variance is heritable and could be changed by selection, supporting previous studies^40–42^. Rönnegard et al.^41^ reported advantages in using breeding values explaining differences in residual variance (vEBV) for milk yield uniformity in Holstein cattle. According to Mulder et al.^40^ the reduction of phenotypic variance by selecting for reduced residual variance could be more beneficial for low heritability traits.

### Meta-analysis of ecoregion-specific GWAA

We performed a meta-analysis of ecoregion-specific GWAA (GWAA for each ecoregion separately) to look for significant differences (Cochran’s Q statistics) in effect size of SNPs between the ecoregions, which are interpreted as G × E^43^. In addition, we calculated the m-value statistic for the significant SNPs (Cochran's Q statistic's *P*-value < 1e−5), which is the posterior probability of an effect being present in an ecoregion given the observations from all other ecoregions^43^ allowing us to identify which ecoregions a given variant has an effect. Southeast and Desert and Arid Prairie ecoregions were not included in the meta-analysis due to their small sample sizes. Meta-analysis of ecoregion-specific GWAA detected 12 G × E SNPs for BW located on chromosome 6 (Additional file 1: Figure S17a); two G × E SNPs for WW on chromosome 15 (Additional file 1: Figure S17b); and two G × E SNPs for YW, one located on chromosome 13 and one on chromosome 15 (Additional file 1: Figure S17c, Additional file 7). Analyzing the PM-plot (*P*-values against m-values) and PB-plot (*P*-values against beta-values, SNP effects estimates obtained from the ecoregion-specific GWAA) of the G × E SNPs detected by the meta-analysis, we observed that some G × E SNPs on chromosome 6 at 30 Mb have an effect on animal’s BW from Forested Mountain and Upper Midwest and Northeast ecoregions, however with different certainty (Additional file 1: Figures S18a and S19a). For WW and YW, despite that the analysis had shown a significant heterogeneity between the ecoregions, they did not show different posterior probabilities of an effect in any ecoregion (m-value), (Additional file 1: Figure S18b,c, respectively). However, large differences in the SNP effect size (beta) were noticed between ecoregions (Additional file 1: Figure S19b,c, respectively).

### Traditional genome-wide association analyses

We also performed traditional GWAA using univariate and multivariate linear mixed models in order to compare with the G × E GWAA results. We detected major QTL on chromosomes 6 (26–48 Mb), 7 (88–90 Mb), 14 (15–30 Mb), and 20 (41–78 Mb) for univariate BW, WW and YW (Additional file 1: Figure S20a–c and Additional file 10). In addition, significant SNPs (P < 1e−5) were identified on chromosomes 3 (53.6 Mb) and 17 (73 Mb) for univariate BW; and on chromosomes 1 (81.1–81.3 Mb), 2 (116.1 Mb), and 5 (96.7 Mb) for univariate WW. The GWAA using multivariate model detected QTL also identified in the univariate GWAA on chromosomes 5, 6, 7, 14, 17, and 20 in addition to on chromosomes 4 (24.5 Mb), 9 (57.9 Mb), 13 (63.3 Mb), 19 (50.5 Mb), and 26 (45.4 Mb) (Additional file 1: Figure S20d and Additional file 9). All genes detected by GWAA are included in the Additional file 10. In total, 240, 92, and 141 haplotype blocks were detected for univariate BW, WW, and YW GWAA, respectively, and 222 haplotype blocks for multivariate analysis.

Out of 948 total significant SNPs using multivariate GWAA analysis for the growth traits, 531 SNPs were located on chromosome 6 (from 26.8 to 48.8 Mb). Likewise, several SNPs interacting with Forested Mountains ecoregion, elevation, precipitation and, mainly, with the temperatures were also detected in the same positions on chromosome 6. Given the importance of chromosome 6 underlying the growth traits, we repeated the multivariate GWAA including six independent significant SNPs (e.g. *rs109849093*, *rs464458177*, *rs110305942*, *rs43459303*, *rs109278547*, and *rs137209027* located at 37,211,057; 37,234,136; 37,418,164; 37,695,352; 38,139,495; and 41,084,765 bp, respectively) as fixed effects in the model. In this way, if those SNPs are causal mutations accounting for QTL effect the loci association signals would be missed. Results showed that even including six significant SNPs (not in LD) simultaneously as fixed effects in the model, a small number of SNPs were still associated (six out of 531 SNPs), indicating that there are possibly multiple causal mutations in this region. Based on the linkage disequilibrium between adjacent significant SNP, we found 166, 49, and 95 haplotype blocks in this region for univariate BW, WW, and YW, respectively, and 131 haplotype blocks for multivariate analysis.

### Comparison of the GWA approaches

A nominal significant threshold value (*P* < 1e−5) was used to equally interpret the results of all GWAA approaches, including continuous and discrete G × E GWAA, vGWAA, ecoregion-specific GWAA, and traditional GWAA. Therefore, the false discovery rate (FDR) was estimated for each analysis (Additional file 2: Table S2). Overall, the FDR showed high values for G × E GWAA and vGWAA suggesting that many significant SNPs in this study may be false positives. However, QQ plots from several analyses showed that *P-*values lower than 1e−5 may show deviation from the null hypothesis, therefore, can be considered significant (Additional file 1: Figures S21–S36). The high FDR for the analyses detecting G × E may be due to the requirement of larger sample size to detect such interactions and the small amount of variation explained by G × E effects. Bolormaa et al.^29^ also reported high FDR for non-additive SNP effects on growth traits in beef cattle. The FDR estimation for traditional GWAA, which detect SNPs with additive effects, was very low varying from 0.007 to 0.014. QQ plots of GWAA are displayed in Additional file 1: Figure S37.

Comparing traditional GWAA with all G × E GWAA results, we verified that 312 SNPs with additive effects also have G × E effects (mainly on chromosome 6, but also on chromosomes 7 and 20). After adjusting the G × E GWAA for genomic control, 68 SNP on chromosome 6 remained significant in both analyses. Some SNPs showed G × E effects even greater than their additive effects on the traits. For example, the SNP *rs136523580* (chromosome 6 at 30,566,685) has an additive effect of 0.41 kg for BW in the total dataset, but its G × E effects is even more impactful (0.67 kg) on the BW of animals from the Forested Mountains ecoregion. These findings show the importance of G × E effects on quantitative traits and the need for precision, ecoregion-specific genomic predictions to optimize animal performance.

Comparing vGWAA with the traditional GWAA results, only one vQTL, on chromosome 14 at 23.1–23.3 Mb harboring the genes *LYN, MOS, RPS20, PLAG1,* and *CHCHD7*, was also found as a direct effect QTL for BW, meaning that this locus affects both mean and variance of BW in this population. Five vQTL were also identified by the G × E GWAA, which were found to interact with multiple continuous environmental variables as well as interacting with ecoregions. However, after genomic control adjustment on the G × E GWAA, only one vQTL was also identified by the G × E GWAA, which was found to interact with mean and minimum temperature. G × E GWAA detected a greater number of G × E loci when compared with the vGWAA results.

Only few G × E SNPs were detected using the meta-analysis of ecoregion-specific GWAA (Cochran's Q statistic's *P*-value < 1e−5), which were also identified by the G × E GWAA. In addition, using the meta-analysis approach the Southeast and Desert and Arid Prairie ecoregions could not be included in the analysis due to their small sample sizes, suggesting that this was not an optimal approach to detect G × E associations for our dataset. However, creating PM-plots (contrast between environment-specific *P*-value and posterior probability of an effect, m-value)^44^, and PB-plots (environment-specific *P*-value and SNP effect size) helped interpret results of our G × E GWAA and vGWAA. For significant G × E SNPs detected by the G × E GWAA, there were clear differences in the SNP effect sizes between the significant ecoregion and the remaining ecoregions (Additional file 1: Figures S38 and S39). For SNPs with significant variance-heterogeneity effects (detected by vGWAA), patterns were not as consistent. While some vQTL had differences in mean effects, others did not (Additional file 1: Figures S40 and S41).

## Discussion

This G × E study provides a more comprehensive understanding of how G × E modulate growth traits in beef cattle and possibly in other mammalian species. We observed that G × E explain substantial proportions of growth traits phenotypic variances, and a great number of G × E SNPs, some with large effects, were detected affecting such traits. Detection of large G × E SNP effects indicates that there is considerable opportunity to improve environmental resistance and performance in cattle. Our study also revealed the first evidence of vQTL controlling phenotypic variation in body weights due to G × ﻿G or G × E effects in beef cattle. In addition, we uncovered several biological pathways affected by G × E that likely drive environmental adaptation.

Among the environmental variables analyzed, birth weight was most affected by temperature. Also, a greater number of G × E QTL for birth weight were detected in animals born in the Fescue Belt and Forested Mountain ecoregions, which have high and low temperatures, respectively. The effect of higher temperature on calf birth weight may be due to the reduced feed intake or the decreased uterine blood flow and placental function often found in heat-stressed cows, factors that contribute to impaired cow-to-fetal nutrient exchange, negatively affecting fetal growth^45–47^. On the other hand, low temperatures can increase calf birth weight due to the fact that the cow’s body directs blood flow toward internal organs, including the uterus, to conserve heat and maintain body temperature, increasing the nutrients being carried to the calf^48^. Weaning weight and yearling weight were more affected by elevation and Forested Mountain, followed by the High Plains, ecoregions with high elevation. Higher altitude regions are associated with reduced atmospheric oxygen (hypoxia), which causes pulmonary hypertension and vasoconstriction leading to right-side heart failure in cattle^49^. This condition is known as brisket disease and accounts for significant losses in growth and reproductive performance, being one of the top causes of mortality in cattle residing at high altitudes^50^. Therefore, the genomic regions detected interacting with Forested Mountain and High Plains ecoregions may be related to adaptation to oxygen deprivation in beef cattle.

Molecular functions involved in neurotransmission processes were enriched by G × E genes. The release of specific neurotransmitters in the brain, such as dopamine, acetylcholine, glutamate, serotonin and GABA, is the main physiological mechanism that organizes the response to a stressful situation^51,52^. Specifically, GABA receptor activity and glutamatergic synapse pathway were enriched. Genes with G × E associations with roles in the activity of the GABA receptor (*GABRA4,* and *GABRB1)* were found interacting with Fescue Belt ecoregion. This may be due to the consumption of tall fescue, a grass commonly used in grazing cattle systems in the Southeastern United States^53^, which usually contains an endophytic fungus that produces ergot alkaloids^54^. Ergot alkaloids have vasoconstrictive effects and interact with GABAergic receptor affecting multiple aspects of animal’s physiology^55–57^. Animals consuming the endophyte-infected tall fescue forage often develop fescue toxicosis, which is characterized by reduced feed intake, weight gain, and blood flow, as well as elevated body temperature associated with heat stress^58–60^. The glutamatergic synapse pathway was enriched mainly by G × E genes interacting with Forested Mountains and/or elevation (*PLA2G4B, PLA2G4E, GRIN2D,* and *GRIK2*), supporting that neurotransmitters respond to the effects of hypoxia^61^.

Endocytosis and VEGF signaling pathways were also enriched by candidate G × E genes interacting with elevation or ecoregions with high altitudes. Endocytosis pathway plays an important role in cell survival under hypoxia condition^62^. VEGF signaling is involved in growth of blood vessels promoting angiogensis and have an important role in response to hypoxia^63^. Both endocytosis and VEGF signaling pathways have previously been related to high altitude adaptation in cattle^64^ and other species^65,66^.

Genes of the keratin (KRT) family (*KRT15, KRT31, KRT32, KRT33A, KRT34,* and *KRT36*) are candidates to interact with Fescue Belt ecoregion. Such G × E genes enriched pathways involved in the keratinocyte differentiation (formation of the cornified envelope and keratinization). Such process is involved in the formation of cornified cell envelope (keratin filaments covalently attached to the cornified envelope) which provides the barrier function of epidermis helping to protect the body against environmental stimuli^67^. Keratin proteins are also involved in the hair-fiber formation cyclical process, which involves growth, regression, resting phases and shedding of the hair shaft^68^. In cattle, hair shedding is an adaptive process that help the animal to cope with heat stress, which is aggravated by fescue toxicosis condition^69^. Durbin et al.^70^ detected genes of the KRT family associated with hair shedding in Angus cattle reared in the Fescue Belt ecoregion. Interestingly, the KRT family genes also enriched the estrogen signaling pathway, which is involved in fertility in cattle^71^. This result may explain, at least in part, fertility problems in heifers grazing endophyte-infected tall fescue^72^.

Genes that are candidates for G × E associations detected in the Upper Midwest & Northeast ecoregion may be involved in metabolic adjustment to adapt to cold weather. Steroid hormone biosynthesis, retinol metabolism and bile secretion are involved in the regulation of thermogenic processes^73–75^. In addition, ascorbate and aldarate metabolism, and pentose and glucuronate interconversions have previously been involved in molecular mechanisms responsive to cold stress^76^.

Pathways related to immune responses, as *Staphylococcus aureus* infection and viral myocarditis were also enriched. Immune functions reflect the involvement of innate and adaptive immune systems in the activation of host defense reactions to deal with pathogens^77^. Many of these immune G × E genes have been associated with diseases caused by pathogens leading to reduced productivity in cattle^78–88^. This result is in agreement that heterogeneity in the immune response to infectious diseases across populations living in different environmental conditions is under genetic control^89^. In addition, pathogen resistance increases host fitness and has driven the evolution of plant and animals through time^90^.

One of our major findings is that the ecoregions showed specific G × E with large effects on the traits. In addition, ecoregion G × E GWAA detected G × E loci that were not identified by the continuous G × E GWAA. One possible explanation for these results is that when analyzing ecoregions, we take into account not only climate and topography, but also unmeasured variables like forage quality and local pathogens, as well as the combination of them. This information allowed us to identify environmentally sensitive genotypes. It indicates that the same trait can be influenced by different genes in the various ecoregions which may affect particular physiological and behavioral functions leading to local adaptation. This assumption is supported by the GO and pathways enrichment analysis of the G × E genes identified for each ecoregion showing that the diverse ecoregion environment affects different biological mechanisms. We also found that major additive QTL also carry G × E effects on growth traits and that some of their G × E effects are greater than their additive effects. Additionally, novel G × E associations were detected in multiple genomic regions. These findings support the importance of creating ecoregion-specific genomic predictions to identify local resilient or adapted animals, using the G × E SNPs detected in this study to assist selection decisions which would lead to an increase in animal performance within an ecoregion. However, the lack of overlap of G × E QTL between the ecoregions may reflect a lack of statistical power to detect such interactions due to the different sample size within the ecoregions.

We showed that vGWAA may be able to detect loci enriched with G × E effects without requiring prior knowledge of the interacting environmental factors. A vGWAA may capture G × E loci that interact with multiple environmental variables, possibly even those not included in the G × E GWAA model (e.g. water availability, pasture condition, pathogens). When multiple interacting factors induce variance heterogeneity, the power of identification of any single one of them or all together may be lower than the power of the variance heterogeneity test^91^, which may explain why vGWAA identified G × E loci that were not detected by direct G × E GWAA. We also observed that some vQTL have differences in means across ecoregions, however, not all do. This suggests that some vQTL could be the difference between robust genotypes (little variation, flat reaction norms) across environments while other genotypes at the same locus are more variable across environments, indicating that selection for low variance vQTL genotypes may provide sustainability advantages. Therefore, genetic variation in residual variance may be utilized to increase uniformity of production in livestock populations^40^. Improvement of uniformity through selection is particularly interesting for traits with an intermediate optimum value (e.g. litter size in sheep, egg weight in laying hens, carcass weight and quality traits in pigs and broilers, and maternal growth in beef), or for decreasing variation in performance within sire’s daughter group^40,41^. Nonetheless, many genes harboring the vQTL identified here were previously reported to be involved in environmental adaptation in cattle^35–39^ supporting that phenotypic variability may be an adaptive evolutionary solution to environmental changes^92^. However, our results support Paré et al.^93^ which reported that when an environmental factor of interest has been measured on all the genotyped individuals, the direct G × E GWAA will be able to detect a larger number of G × E loci.

Our study also demonstrated the challenges of detecting G × E effects. Although we have used a relatively large beef cattle population to date, it still seems that a greater number of animals representing all possible environments are needed to detect such interactions. Also, several G × E QTL have small effect sizes on quantitative traits increasing the difficulty to differentiate them from potential bias. These limitations directly impact the statistical power to detect G × E as previously reported. For example, G × E studies observed unclear evidence of physical activity modulating the effects of *FTO* gene variants on obesity in humans^94–96^ until a study with large sample size (~ 218,000 individuals) confirmed this interaction^97^. Therefore, studies using a larger population size could validate our results.

The environmentally affected biological mechanisms that modulate growth traits identified in this study have been reported to be involved in resilience^12,52,77^, and adaptation processes^10,49,64,98,99^, revealing the role that G × E plays in adaptation and evolution at the level of the genome in cattle and potentially across mammals. It is important to understand such biological mechanisms since the inability of an animal to cope with its environment results in failure to produce optimally leading to lost potential profitability of livestock operations^12,50,100,101^. Further, these biological insights may help us understand how adaptation occurs in wild populations and the potential effect of climate change on animal physiological functions.

## Conclusions

G × E GWAA and vGWAA detected several loci affecting growth traits that the traditional GWAA did not, showing that functional loci may have non-additive genetic effects between genotype classes regardless of differences in genotypic means. We revealed biological mechanisms by which beef cattle respond to changes in their environment. Neurotransmitter activity, hypoxia-induced processes, keratinization, hormone, thermogenic and immune pathways were highlighted, indicating that climate change may become a burden on animal health and productivity. In addition, ecoregion-specific G × E SNPs detected in this study may play a crucial role in resilience and adaptation of beef cattle to divergent environments. Although conventional genomic selection has shown great promise in improving genetic gain, it only considers additive effects and, according to our findings, ecoregion-specific genomic predictions should be created to identify animals best suited for given environments. This could help producers in making optimal decisions given their geographical location. Our results revealed novel trait associations and alternative genetic mechanisms involved in shaping the total phenotypic variance of growth traits providing new insights into how the environment influences such traits and adaptation in beef cattle and other mammalian species.

## Methods

### Phenotype, genotype and environmental data

Data from the American Simmental Association males and females born between 1975 and 2016 and genotyped for genomic-enhanced expected progeny differences (EPDs, i.e. genomic predictions) were transferred to the University of Missouri. Bulls who were born decades ago were genotyped from cryopreserved semen used for artificial insemination. The traits studied were birth weight (BW), 205-d adjusted weaning weight (WW) and 365-d adjusted yearling weight (YW), with the traits also adjusted for age of dam as per breed association practice. The animals were assigned to contemporary groups (CG), defined by the combination of farm, season (spring vs. fall) and year of birth.

The DNA samples were genotyped with various low-density assays (GeneSeek GGP-LDv3, GeneSeek GGP-LDv4, GeneSeek GGP-90KT, GeneSeek GGP-HDv3, GeneSeek GGP-F250, IlluminaSNP50, and IlluminaHD) and were imputed to the combination of Illumina BovineHD (Illumina, San Diego, CA) and the GeneSeek Genomic Profiler F250 (GeneSeek, Lincoln, NE) according to Rowan et al.^102^. Briefly, the imputation pipeline begins with genotype filtering to remove non-autosomal variants, as well as both variants and individuals with call rates less than 0.90, using PLINK 1.9^103^. SNP positions were based on the ARS-UCD1.2 Bovine reference genome assembly^104^ and all genotypes were coded as reference versus alternate alleles as A, C, G, and T bases. The genotypes were then phased using Eagle 2.4^105^ and imputed using Minimac3^106^. The final imputed genotype data contained a total of 835,947 bi-allelic variants with imputation accuracy of 99.6%^102^. Variants with minor allele frequency less than 0.01 were removed using PLINK 1.9 for further analysis, leaving 710,202, 709,054, and 706,771 SNP markers for 13,427, 11,847, and 8546 animals for BW, WW and YW, respectively.

Phenotypes were adjusted for fixed effect of sex and random effect of CG. A random maternal permanent environmental effect was also included in the WW adjustment to account for differences in milk production and maternal care of the animal’s dam. For G × E GWAS models, birth weights were analyzed twice, once adjusted for a maternal effect and once without. Fixed effect of sex was estimated using ‘--reml-est-fix’ option, and random effects were predicted by the BLUP method using ‘--reml-pred-rand’ flag while controlling for population stratification using single-trait animal model implemented in GCTA software^107^. Contemporary groups were included in the univariate model as random effect since 19% of individuals analyzed belong to CG with only a single animal^108^. After removing confounding management effects, the pre-adjusted phenotypes were used in the downstream analyses.

Eight continuous environmental variables were used for the G × E GWAA, including information of 30-year normal of minimum, mean, maximum temperatures and mean dew point temperature (^o^C); elevation (m); precipitation (ml); and minimum and maximum vapor pressure deficit (hPs) data, which were drawn from the PRISM climate dataset^109^. The United States was partitioned into nine regions (Fig. 2f*,* top) based on similar topographic and environmental conditions (ecoregions), including mean temperature, elevation and precipitation information, using k-means clustering implemented in ‘RStoolbox’ R package^110,111^. The optimal ecoregions were identified using ‘pamk’ function from the R package ‘fpc’^112^. The ecoregions were named Desert (D), High Plains (HP), Arid Prairie (AP), Forested Mountains (FM), Upper Midwest and Northeast (UN), Southeast (SE), Rainforest (R), Foothills (FH), and Fescue Belt (FB)^10^. Animals were assigned to the ecoregions based on the zip code of their breeder (Fig. 2f*,* bottom), and only ecoregions with more than 200 records were analyzed. Thus, animals located in the Rainforest ecoregion were removed from the dataset, and those that were reared in the Desert and Arid Prairie ecoregions were analyzed together (in a group referred to as DA) due to their similar environmental conditions. No animal record was assigned to the Foothills ecoregion. A complete description of the dataset, including weight records and environmental variable measurements is shown in Additional file 2: Table S2.

### Estimation of G × E variance components

In order to estimate the amount of phenotypic variance explained by the G × E interactions among the ecoregions, G × E variance component for pre-adjusted BW, WW, and YW were estimated. This analysis was performed using multi-component restricted maximum likelihood (REML) approach, implemented in GCTA software, considering the ecoregions as an environmental factor in the univariate linear mixed model as follows:

$$\user2{y}^{*} = \mu + \user2{eco} + \user2{u} + \user2{gxe} + \user2{e}$$

where y^*^ is the vector of pre-adjusted phenotypes; $\mu$ is the overall mean; ***eco*** is the fixed effects of the environmental factor (ecoregion); ***u*** is the vector of random additive genetic effects; G × E is the vector of random G × E effects; and ***e*** is the vector of random residuals. Assuming that $\user2{u}\sim N(0,\;\user2{G}\sigma _{a}^{2} )$, $\user2{gxe}\sim N(0,\;\user2{G}_{{gxe}} \sigma _{{gxe}}^{2} )$, $\user2{e}\sim N(0,\;\user2{I}\sigma _{e}^{2} )$, where $\sigma _{a}^{2}$, $\sigma _{{gxe}}^{2}$, and $\sigma _{e}^{2}$ are the additive, genotype-by-environment, and residual variances, respectively. ***I*** is the identity matrix and ***G*** is the genomic relationship matrix. The ***G*** matrix was calculated using SNP marker information according to^107^ as: $\user2{G} = \user2{W}_{G} \user2{W}_{G}^{'} /N$, where $w_{{G(i)}} = (x_{{G\left( i \right)}} - 2p_{i} )/\sqrt {2p_{i} (1 - p_{i} )}$, with $x_{G}$ being 0, 1, or 2 for genotypes AA, AB or BB, respectively; $p_{i}$ is the reference allele frequency at SNP $i$; and *N* is the number of SNPs. ***G***_G × E﻿__[*i,j*]_ is ***G***_[*i,j*]_ if *i* and *j* are from the same ecoregion and zero otherwise^113^.

### Genome-wide association analyses (GWAA)

Univariate linear mixed model analyses were performed for the pre-adjusted BW, WW or YW using GEMMA software^114^ as follows:

$$\user2{y}^{*} = 1\mu + \user2{X\beta } + \user2{Zu} + \user2{e}$$

where y^*^ is an *n*-vector of pre-adjusted phenotypes; $\mu$ is the overall mean; ***X*** is the incidence matrix of genotypes; $\user2{\beta }$ is the genotype effects; ***Z*** is the design matrix for random animal effects, ***u*** is an *n*-vector of random additive genetic effects; and ***e*** is an *n*-vector of random residual. Assuming that $\user2{u} \sim N(0,\;\user2{G}\sigma _{a}^{2} )$ and $\user2{e} \sim N(0,\;\user2{I}\sigma _{e}^{2} )$, ***G*** is the *n* × *n* genomic relationship matrix; $\sigma _{a}^{2}$ is the additive genetic variance; $\sigma _{e}^{2}$ is the residual variance component; ***I*** is an *n* × *n* identity matrix. Genomic relationship matrices were estimated using the standardized genotypes of 710,202, 709,054, and 706,771 SNP markers for 13,427, 11,847, and 8546 animals for BW, WW and YW, respectively.

We also fitted a multivariate linear mixed model for the pre-adjusted BW, WW and YW using GEMMA^115^ in the following form:

$$\user2{Y}^{*} = 1\mu + \user2{X\beta } + \user2{ZU} + \user2{E}$$

where Y^*^ is an *n* × *d* matrix of *d* pre-adjusted phenotypes for *n* individuals; $\mu$ is the overall mean; ***X*** is the incidence matrix of genotypes; $\user2{\beta }$ is a *d* vector of the genotype effects for the *d* phenotypes; **Z** is the design matrix for random animal effects; ***U*** is an *n* × *d* matrix of random additive genetic effects; and ***E*** is an *n* × *d* matrix of random residuals. $\user2{U} \sim N(0,\;\user2{G} \otimes \user2{V}_{\user2{a}} )$ and $\user2{E} \sim N(0,\;\user2{I} \otimes \user2{V}_{\user2{e}} )$, ***G*** is the *n* × *n* genomic relationship matrix; ***V***_***a***_ is the *d* × *d* additive genetic (co)variance matrix; ***V***_***e***_ is a *d* × *d* residual (co)variance matrix; ***I*** is an *n* × *n* identity matrix. ⊗ denotes Kronecker product operation. For all GWAA performed, single-marker *P*-values were used to generate Manhattan and QQ plots using ‘manhattan’ and ‘qq’ functions, respectively, implemented in ‘qqman’ R package^116^.

### Genotype-by-environment GWAA

The G × E GWAA were performed using GEMMA through two approaches: using eight continuous environmental variables (i.e. minimum, mean, maximum and mean dew point temperatures; elevation; precipitation; and minimum and maximum vapor pressure deficit) separately; and using the ecoregions as discrete environmental variable, where each ecoregion was compared against the total dataset using 0 and 1 dummy coding. In the G × E GWAA, for each SNP in turn, GEMMA fits a linear mixed model that controls both the SNP main effect and environmental main effect, while testing for the interaction effect and controlling for population stratification, evaluating the alternative hypothesis (H_1_: $x_{i} *x_{j} \beta _{{ij}}$ ≠ 0) against the null hypothesis (H_0_:$~x_{i} *x_{j} \beta _{{ij}}$ = 0) for each interaction, therefore the resulting *P*-values correspond to the significance of the G × E interaction^31^. This model can be described as:

$$\user2{y}^{*} = 1\mu + \user2{x}_{i} \user2{\beta }_{i} + \user2{x}_{j} \user2{\beta }_{j} + \user2{x}_{i} *\user2{x}_{j} \user2{\beta }_{{ij}} + \user2{u} + \user2{e}$$

where y^*^ is an n-vector of pre-adjusted phenotypes; ***x***_*i*_ is an *n*-vector of the genotypes of the SNP *i*; $\user2{\beta }_{i}$ is the additive effect of the SNP *i*; ***x***_*j*_ is an *n*-vector of environmental variable *j*; $\user2{\beta }_{j}$ is the fixed effect of the environmental variable *j*; $\user2{\beta }_{{ij}}$ is the interaction effect between the genotypes of the SNP *i* and the environmental variable *j*; ***u*** is an *n*-vector of random additive genetic effects [$\user2{u} \sim N(0,\;\user2{G}\sigma _{a}^{2} )$]; and ***e*** is an *n*-vector of random residual [$\user2{e} \sim N(0,\;\user2{I}\sigma _{e}^{2} )$]. G × E GWAA using multivariate linear mixed model were also performed for all ecoregions but Desert and Arid Prairie due to small sample size.

### Variance-heterogeneity GWAA

Variance-heterogeneity GWAA (vGWAA) can detect loci affecting the difference in variance between genotypes (vQTL), which can be explained by G × E or GxG (epistatic) interactions^27^. Therefore, other possible effects on the phenotypes should be removed in the model in order to detect G × E using vGWAA approach. To do this, we estimated additive, dominance and epistasis variances for BW, WW, and YW using REML approach in univariate linear mixed models, using GCTA software. We fitted two different models (M): including only additive effects (MA), or using additive, dominance, and epistasis effects (MADE) as follows:

$$\user2{y}^{*} = 1\mu + \user2{Z}_{u} \user2{u} + \user2{e}_{A} \quad ({\text{MA}})$$

$$\user2{y}^{*} = 1\mu + \user2{Z}_{u} \user2{u} + \user2{Z}_{d} \user2{d} + \user2{Z}_{{ep}} \user2{ep} + \user2{e}_{{ADE}} \quad ({\text{MADE}})$$

where y^*^ is the vector of pre-adjusted phenotypes; $\mu$ is the overall mean; ***u*** is the vector of random additive genetic effects; ***d*** is the vector of random dominance effects; ***ep*** is the vector of random epistatic effects; and ***e***_*A*_ and ***e***_*ADE*_ are vectors of random residuals for MA and MADE models, respectively. ***Z ***matrices are incidence matrices relating observations to animals. Assuming that $\user2{u}\sim N(0,\;\user2{G}\sigma _{a}^{2} )$, $\user2{d}\sim N(0,\;\user2{D}\sigma _{d}^{2} )$, $\user2{ep}\sim N(0,\;\user2{E}\sigma _{{ep}}^{2} )$, $\user2{e}_{A} \sim N(0,\;\user2{I}\sigma _{{e_{A} }}^{2} )$, $\user2{e}_{{ADE}} \sim N(0,\;\user2{I}\sigma _{{e_{{ADE}} }}^{2} )$, where $\sigma _{a}^{2}$, $\sigma _{d}^{2}$, $\sigma _{{ep}}^{2}$, $\sigma _{{e_{A} }}^{2}$, and $\sigma _{{e_{{ADE}} }}^{2}$ are the additive, dominance, epistatic, MA residual, and MADE residual variances, respectively. ***I*** is the identity matrix; and ***G***, ***D***, and ***E*** are the additive (genomic), dominance and epistatic genetic relationship matrices, respectively. The ***G*** and ***D*** matrices were calculated using SNP marker information^107,117^ as: $\user2{G} = \user2{W}_{G} \user2{W}_{G}^{'} /N$, and $\user2{D} = \user2{W}_{D} \user2{W}_{D}^{'} /N$, where $w_{{G(i)}} = (x_{{G(i)}} - 2p_{i} )/\sqrt {2p_{i} (1 - p_{i} )}$ and , $w_{{D(i)}} = (x'_{{D(i)}} - 2p_{i}^{2} )/\left\lfloor {2p_{i} (1 - p_{i} )} \right\rfloor$ with $x_{G}$ being 0, 1, or 2 and $x'_{D}$ being 0, $2p$, or $(4p - 2)$ for genotypes AA, AB or BB, respectively; $p_{i}$ is the reference allele frequency at SNP $i$; and *N* is the number of SNPs. According to Henderson^118^ ***E*** can be derived from the additive genomic relationship matrix as: $\user2{E}$ = $\user2{G}$ # $\user2{G}$, where # denotes the Hadamard product operation.

Residual effects (***e***_*A*_ and ***e***_*ADE*_) from the MA and MADE models, respectively, were standardized to z score by the rank-based inverse-normal transformation and squared (z^2^), which is a measure of phenotypic variance^119,120^. The vGWAA were performed for BW z^2^, WW z^2^, and YW z^2^ using univariate and multivariate linear mixed models implemented in GEMMA software as denoted in “Genome-wide association analyses (GWAA)” section, where y^*^ is an *n*-vector of z^2^; and $\user2{\beta }_{i}$ is the effect size of the SNP on z^2^.

### Meta-analysis of ecoregion-specific GWAA

In order to evaluate how significantly the effect sizes of SNPs (Cochran’s Q test) varied between the ecoregions (indicative of G × E)^43^ we combined the outputs from univariate within-ecoregion GWAA (GWAA for each ecoregion separately) into a single meta-analysis for each phenotype using METASOFT software^121^. Only ecoregions with more than 1000 animals were analyzed. In addition, the statistic m-value (posterior probability of an effect) for the significant SNPs (Cochran's Q statistic's *P*-value < 1e−5) were calculated, which is an estimate of the posterior probability of a locus having an effect in a particular ecoregion^122^. The results were visualized through PM-plots and PB-plots, in which ecoregion-specific *P*-values are simultaneously visualized with the m-values and beta-values (SNP effects estimates obtained from the ecoregion-specific GWAA) at each tested locus, respectively.

### Genomic control and false discovery rate estimation

In G × E GWAA samples can have shared environmental effects outside the environmental variable used in the G × E GWAA which may cause spurious signals^13^ similar to cryptic relatedness or population structure. Thus, we calculated the genomic control inflation factor (λ_GC_) for all analyses^123^ to improve the calibration of the G × E GWAS *P-*values. For those G × E GWAA with λ_GC_ > 1, *P-*values were divided by the λ_GC_.

A nominal significant threshold value (*P* < 1e−5) was used to equally compare the results of all GWAA approaches, including continuous and discrete G × E GWAA, vGWAA, ecoregion-specific GWAA, and traditional GWAA. Therefore, false discovery rate (FDR) of the nominal significant threshold (*P* < 1e−5) was estimated for all analyses performed (Additional file 2: Table S2), based on Bolormaa et al.^124^, as:

$FDR = P\left( {1 - \frac{A}{T}} \right)/\left( {\frac{A}{T}} \right)\left( {1 - P} \right)$,where *P* is the nominal significant threshold (e.g., 1e−5); *A* is the number of SNP that were significant at the nominal significant threshold; and *T* is the total number of SNPs tested. Although the true FDR cannot exceed 1, the estimated FDR can exceed 1 if the number of significant SNPs is smaller than expected by chance, meaning that all SNPs that met the nominal significant threshold are likely false positives^29^.

### QTL definition

For all analysis done in this study, QTL were defined based on haplotype blocks^125^ using HaploView software, version 4.2^126^. This methodology defines that pairs of SNP markers are in strong linkage disequilibrium (LD) if the confidence interval is equal to or greater than 95% with D′ equal to 0.98 and the lower limit above 0.70.

### Functional enrichment analyses

We explored 100 kb sequence windows that flanked the significant SNPs (*P* < 1e−5) to scan for putative G × E genes located in their vicinity and to identify possible regulatory elements based on the ARS-UCD1.2 bovine reference genomic positions. Enrichment analysis using the G × E candidate genes based on Gene Ontology (GO), and KEGG^127^ and Reactome pathways were performed using g:Profiler^128^, in which the analyses were adjusted for FDR of 10%. GO annotations were also retrieved from the AmiGO browser^129^.

### Ethics approval and consent to participate

Animal Care and Use Committee approval was not required or obtained for data that were extracted from the existing American Simmental Association database.

## Supplementary Information

Supplementary Information 1.

Supplementary Information 2.

Supplementary Information 3.

Supplementary Information 4.

Supplementary Information 5.

Supplementary Information 6.

Supplementary Information 7.

Supplementary Information 8.

Supplementary Information 9.

Supplementary Information 10.

## Data Availability

The raw data used in this research are available from the American Simmental Association under a Data Use Agreement, but are not publicly available. Derived data (analytical results) are however available as supplementary files associated with this publication.
