# Supplementary material for: Genome-wide association analyses identify genotype-by-environment interactions of growth traits in Simmental cattle
Source: Sci Rep. 2021 Jun 25;11:13335. doi: 10.1038/s41598-021-92455-x (PMC8233360; doi:10.1038/s41598-021-92455-x)
Supplement: Supplementary file 1 — Supplementary Information 1. [file 41598_2021_92455_MOESM1_ESM.docx]

**Genome-wide association analyses identify genotype-by-environment interactions of growth traits in Simmental cattle**

Camila U. Braz, Troy N. Rowan, Robert D. Schnabel, Jared E. Decker

Additional file 1

Supplementary Figures


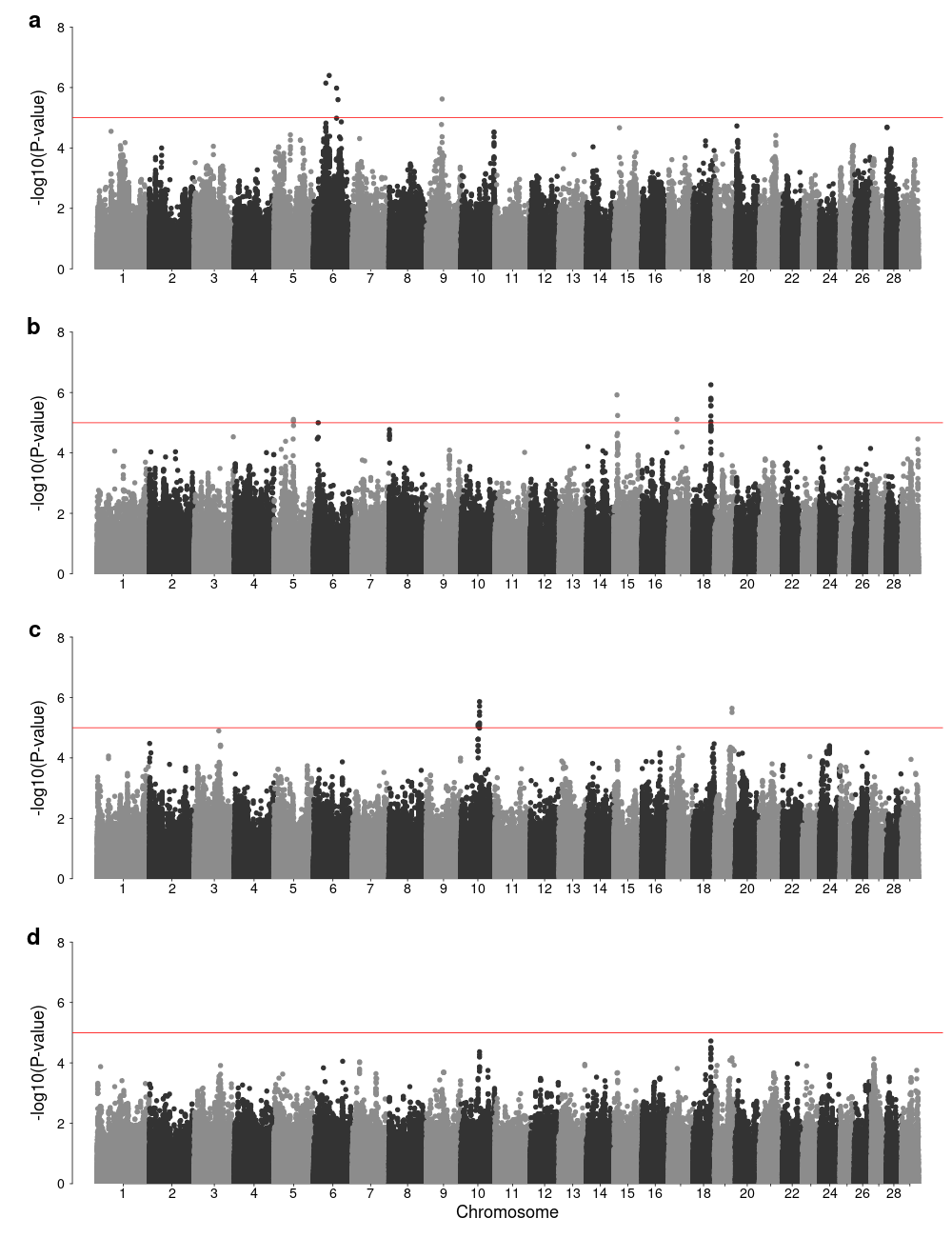
Figure S1. Manhattan plots of genotype-by-environment genome-wide association analysis using elevation as environmental variable for birth weight (a), weaning weight (b), yearling weight (c), and using multivariate analysis (d). Horizontal red line indicates a significant threshold (*P* < 1e-5).


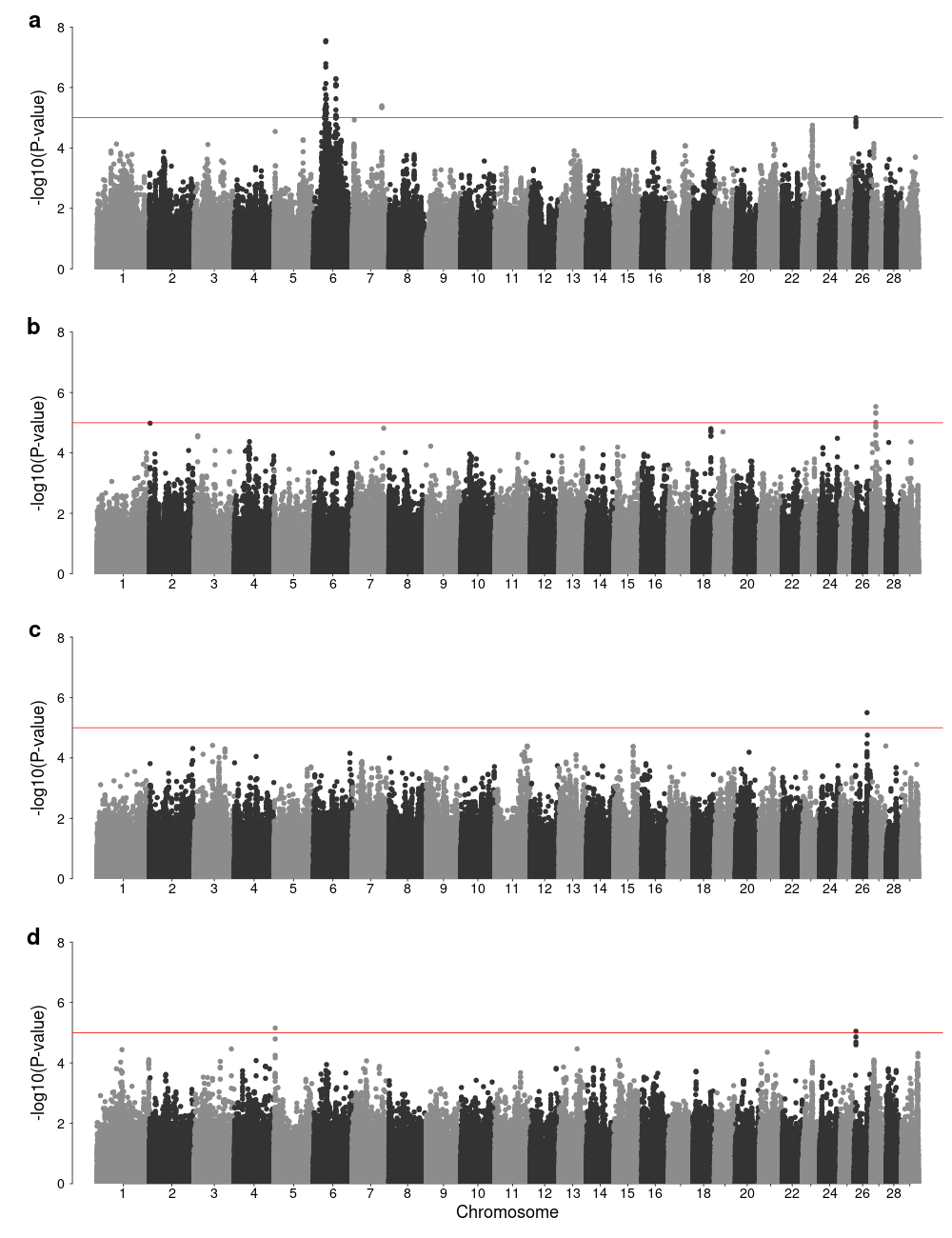
Figure S2. Manhattan plots of genotype-by-environment genome-wide association analysis using precipitation as environmental variable for birth weight (a), weaning weight (b), yearling weight (c), and using multivariate analysis (d). Horizontal red line indicates a significant threshold (*P* < 1e-5).


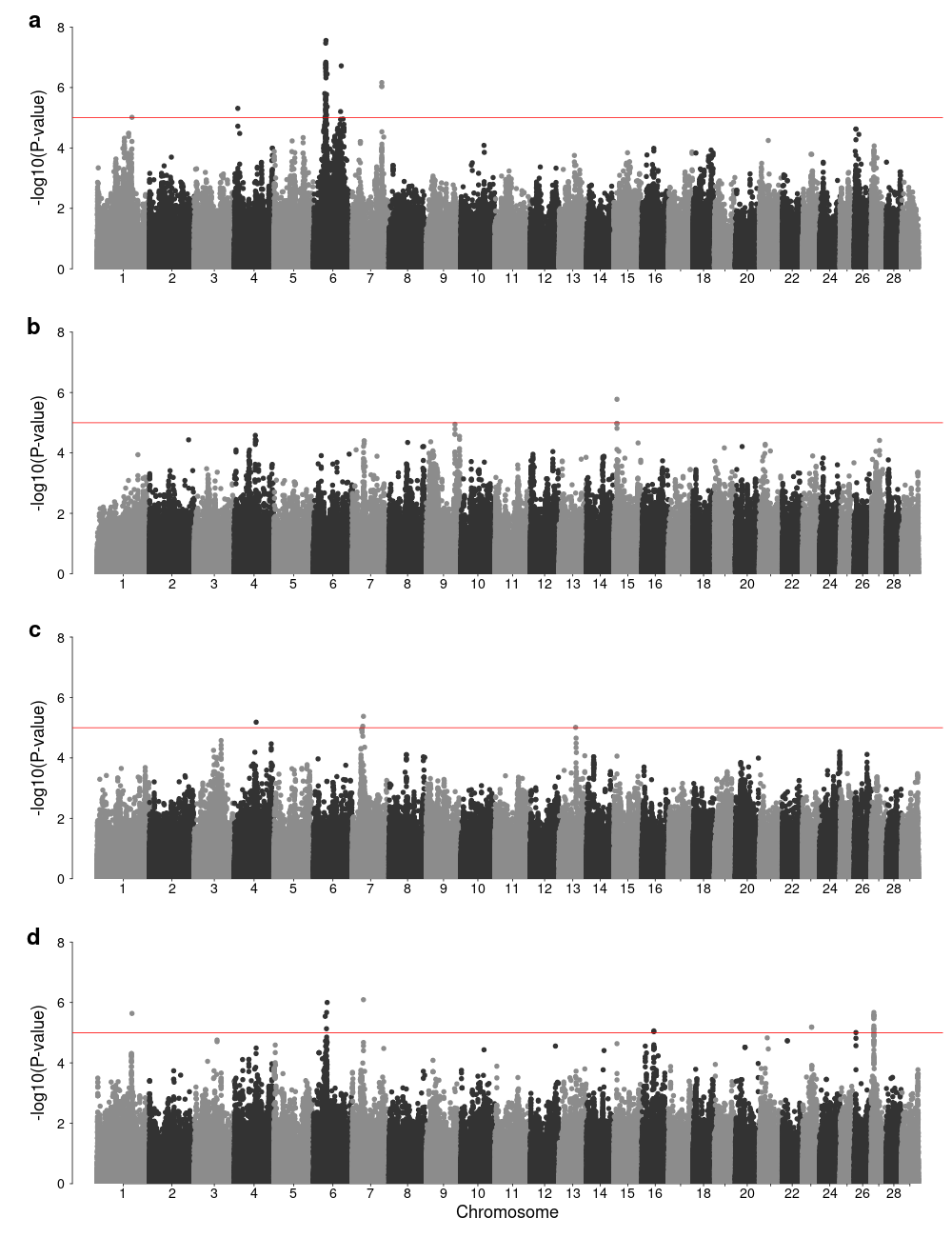
Figure S3. Manhattan plots of genotype-by-environment genome-wide association analysis using mean temperature as environmental variable for birth weight (a), weaning weight (b), yearling weight (c), and using multivariate analysis (d). Horizontal red line indicates a significant threshold (*P* < 1e-5).


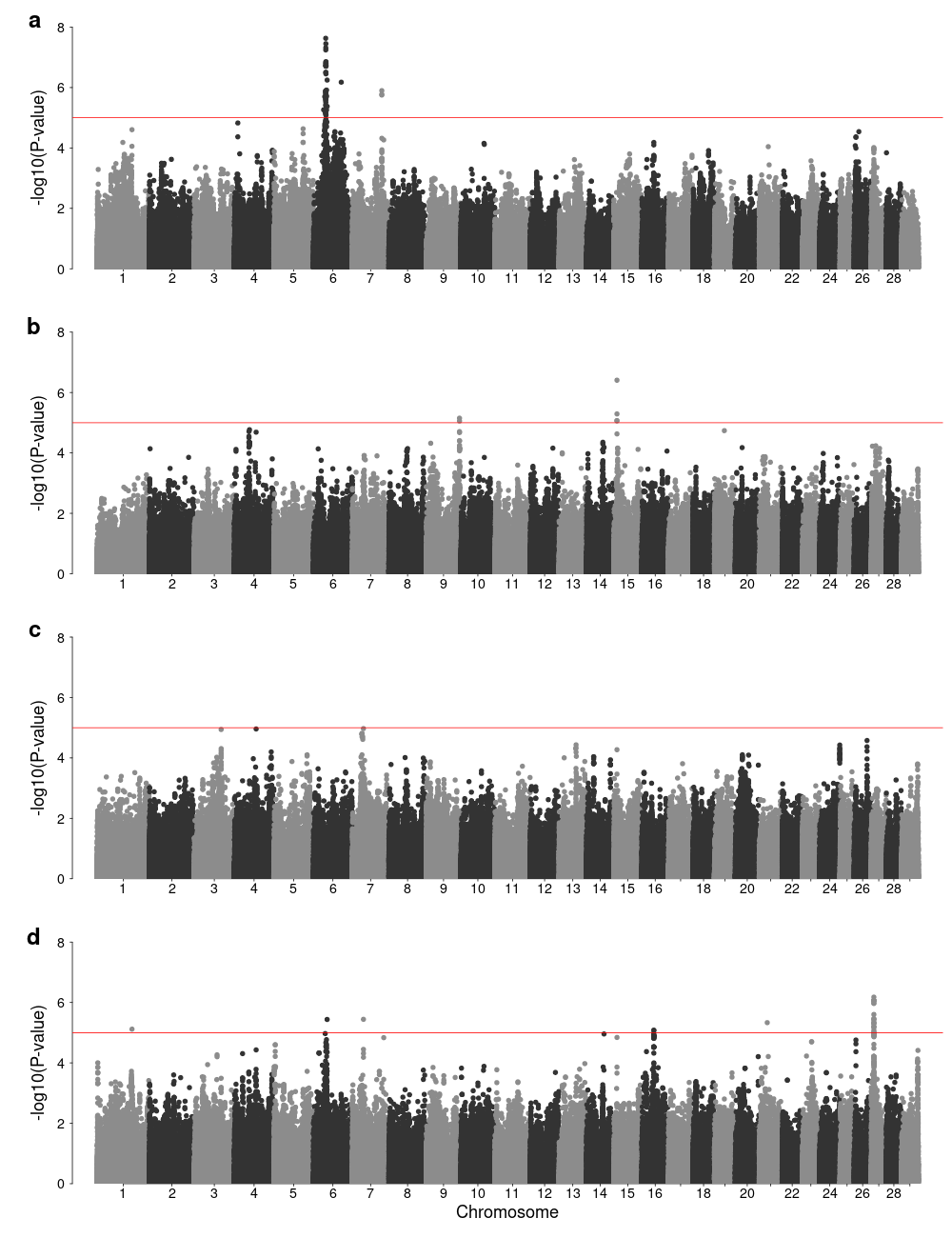
Figure S4. Manhattan plots of genotype-by-environment genome-wide association analysis using minimum temperature as environmental variable for birth weight (a), weaning weight (b), yearling weight (c), and using multivariate analysis (d). Horizontal red line indicates a significant threshold (*P* < 1e-5).


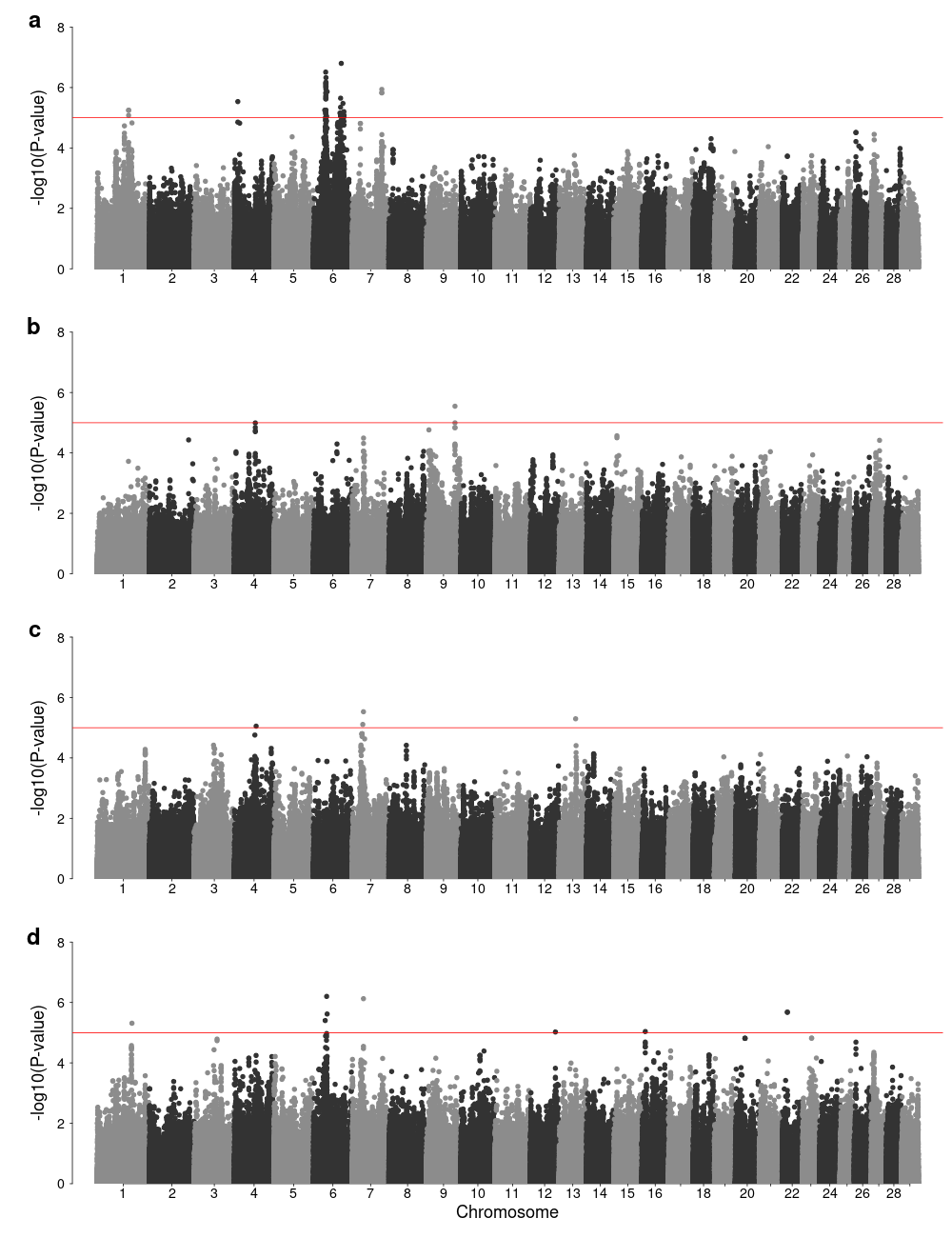
Figure S5. Manhattan plots of genotype-by-environment genome-wide association analysis using maximum temperature as environmental variable for birth weight (a), weaning weight (b), yearling weight (c), and using multivariate analysis (d). Horizontal red line indicates a significant threshold (*P* < 1e-5).


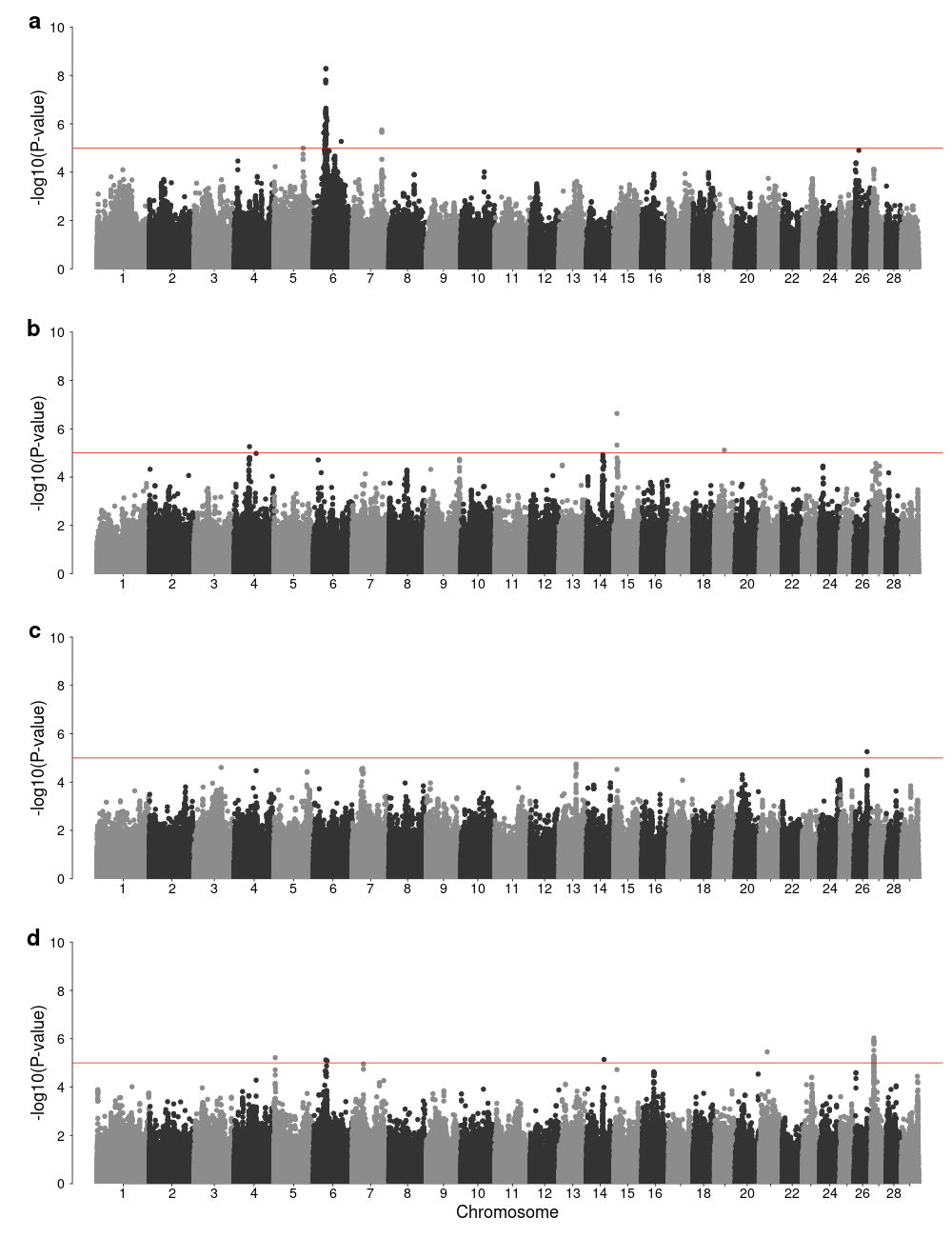
Figure S6. Manhattan plots of genotype-by-environment genome-wide association analysis using mean dew point temperature as environmental variable for birth weight (a), weaning weight (b), yearling weight (c), and using multivariate analysis (d). Horizontal red line indicates a significant threshold (*P* < 1e-5).


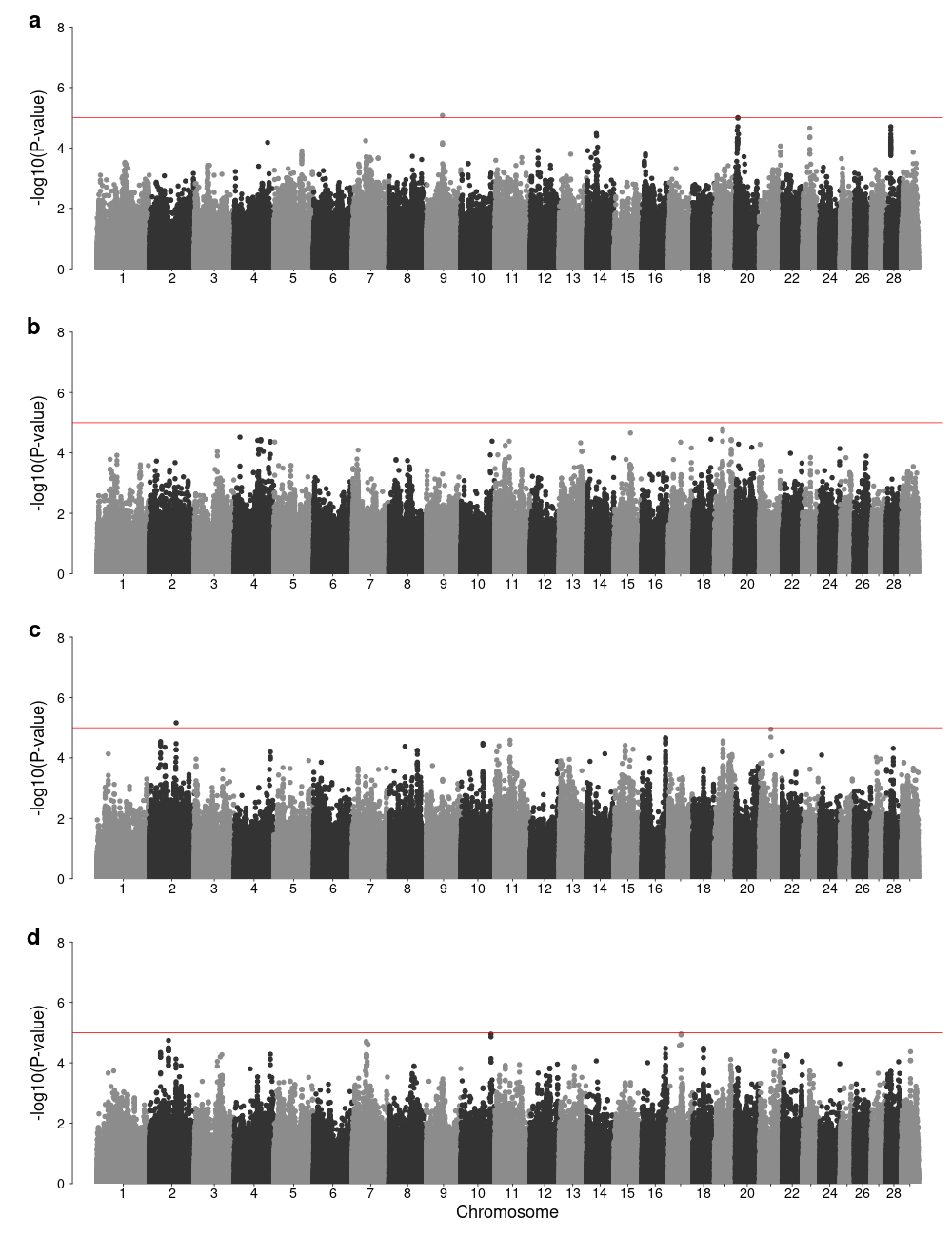
Figure S7. Manhattan plots of genotype-by-environment genome-wide association analysis using minimum vapor pressure deficit as environmental variable for birth weight (a), weaning weight (b), yearling weight (c), and using multivariate analysis (d). Horizontal red line indicates a significant threshold (*P* < 1e-5).


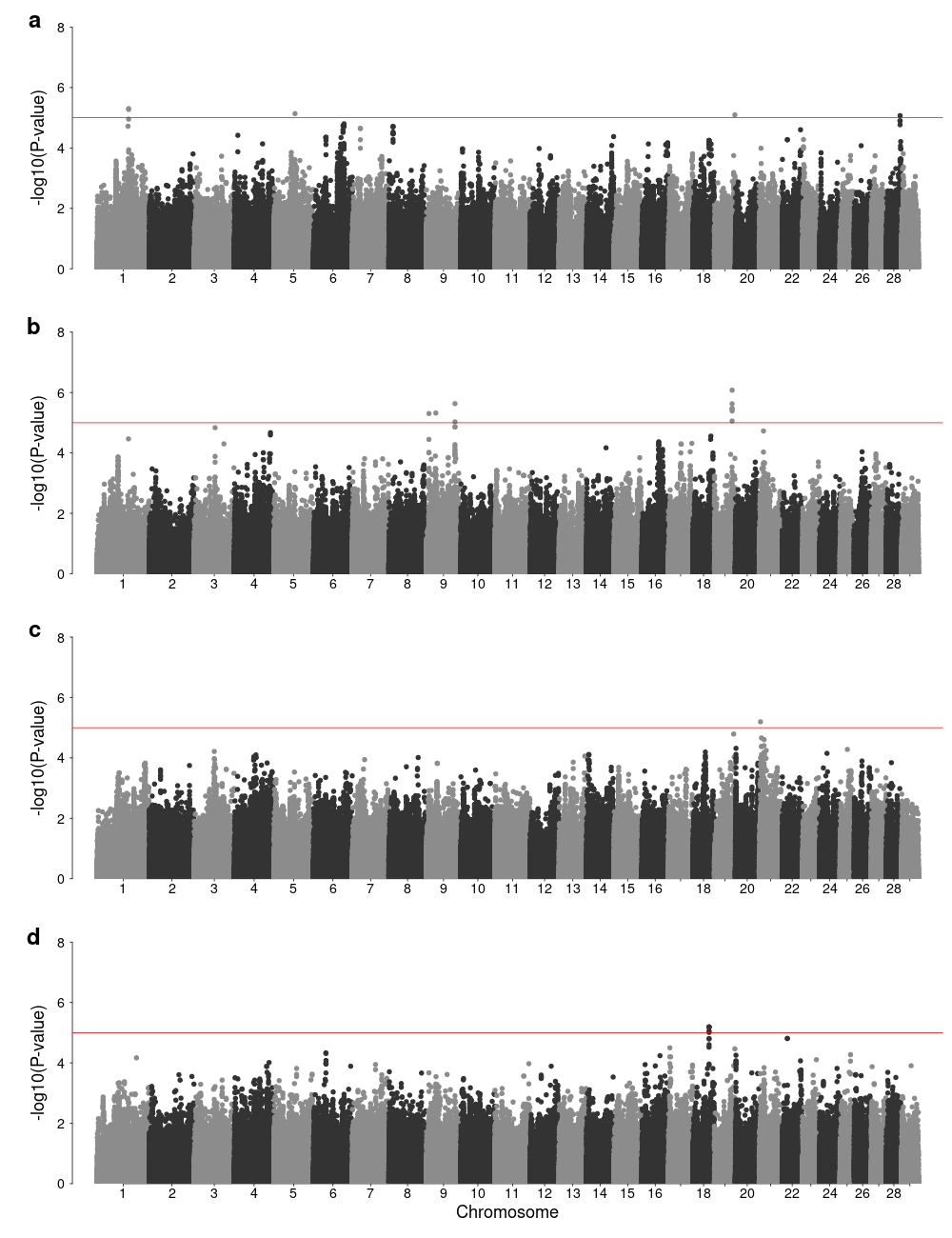
Figure S8. Manhattan plots of genotype-by-environment genome-wide association analysis using maximum vapor pressure deficit as environmental variable for birth weight (a), weaning weight (b), yearling weight (c), and using multivariate analysis (d). Horizontal red line indicates a significant threshold (*P* < 1e-5).


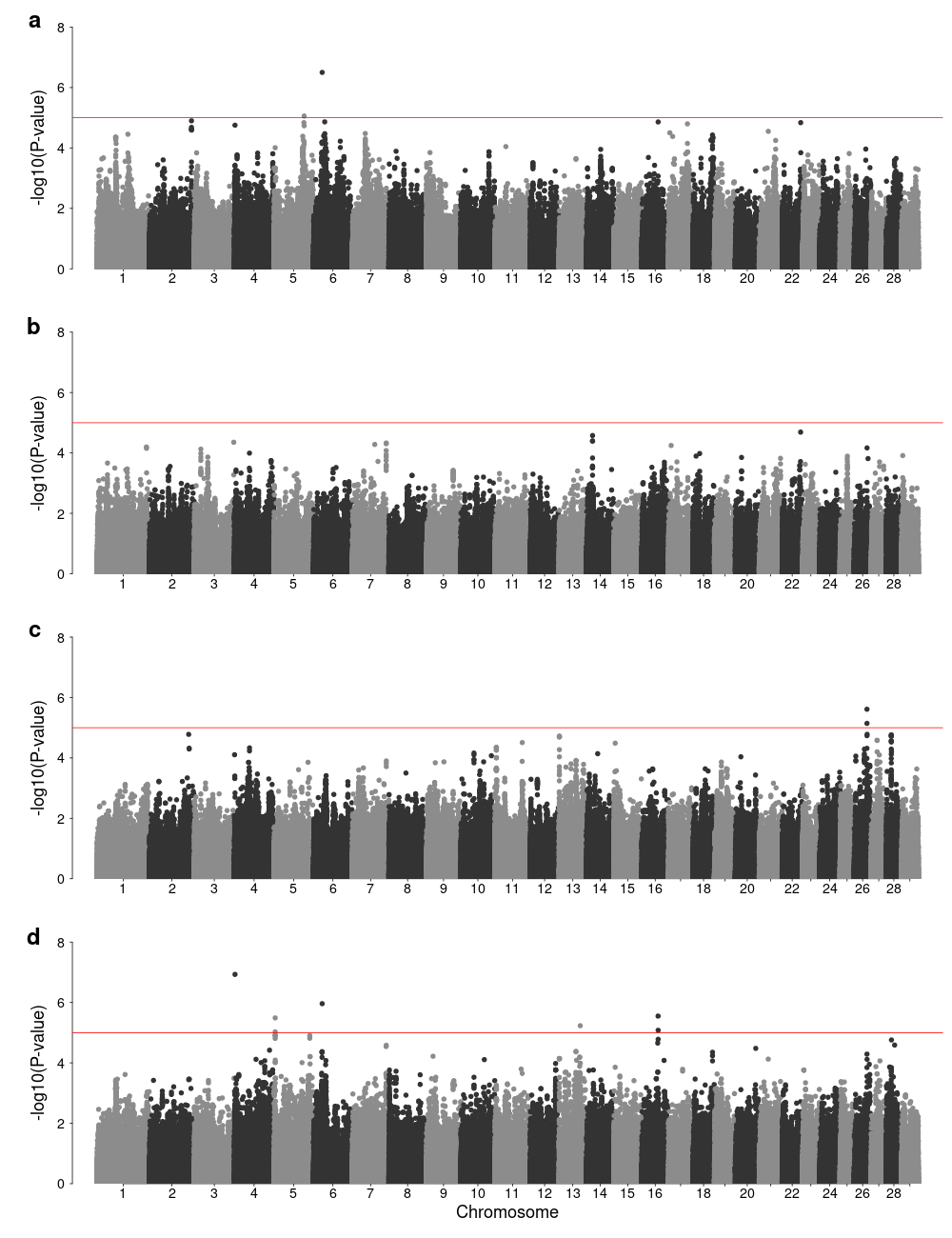
Figure S9. Manhattan plots of genotype-by-environment genome-wide association analysis using Southeast ecoregion as environmental factor for birth weight (a), weaning weight (b), yearling weight (c), and using multivariate analysis (d). Horizontal red line indicates a significant threshold (*P* < 1e-5).


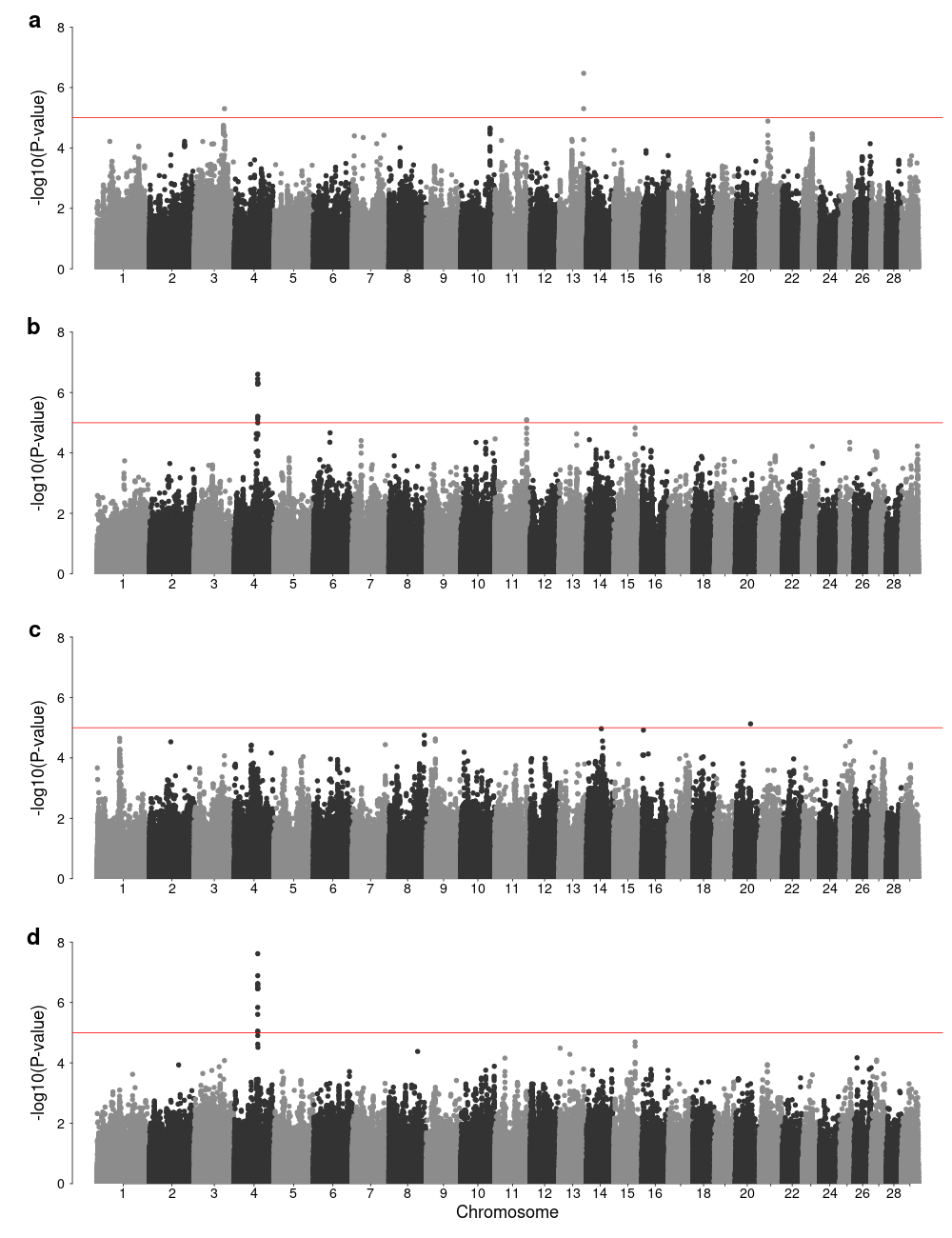
Figure S10. Manhattan plots of genotype-by-environment genome-wide association analysis using High Plains ecoregion as environmental factor for birth weight (a), weaning weight (b), yearling weight (c), and using multivariate analysis (d). Horizontal red line indicates a significant threshold (*P* < 1e-5).


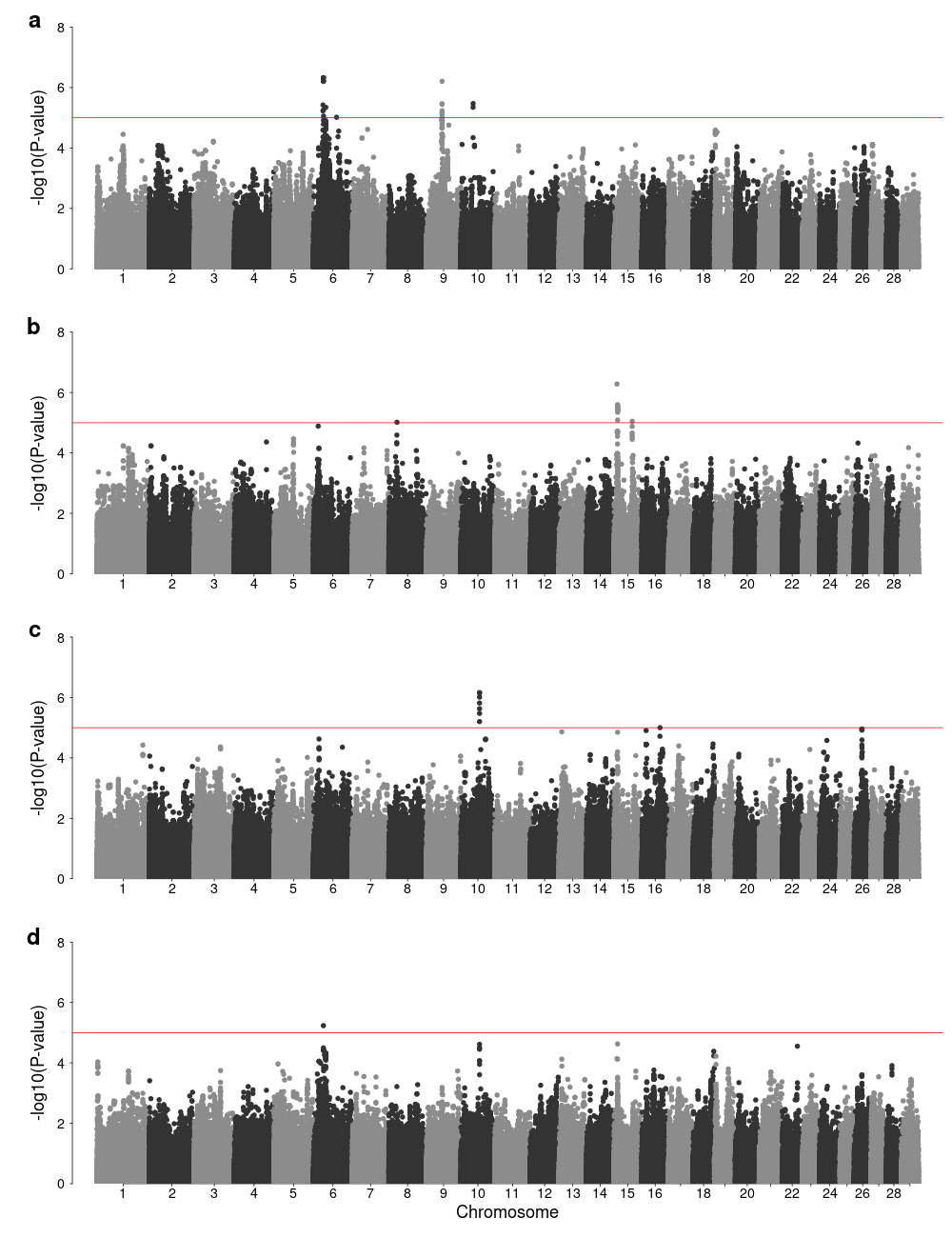
Figure S11. Manhattan plots of genotype-by-environment genome-wide association analysis using Forested Mountains ecoregion as environmental factor for birth weight (a), weaning weight (b), yearling weight (c), and using multivariate analysis (d). Horizontal red line indicates a significant threshold (*P* < 1e-5).


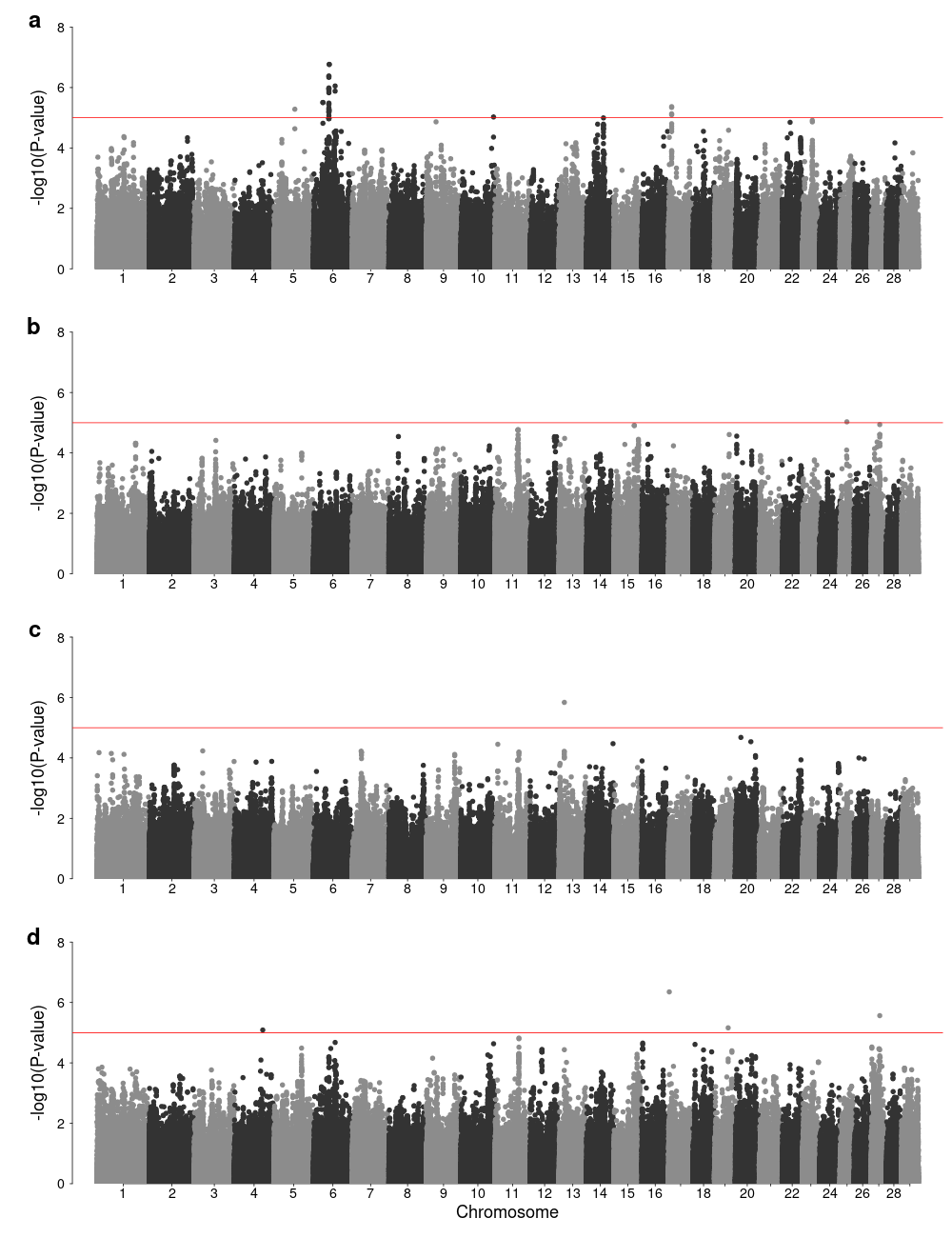
Figure S12. Manhattan plots of genotype-by-environment genome-wide association analysis using Fescue Belt ecoregion as environmental factor for birth weight (a), weaning weight (b), yearling weight (c), and using multivariate analysis (d). Horizontal red line indicates a significant threshold (*P* < 1e-5).


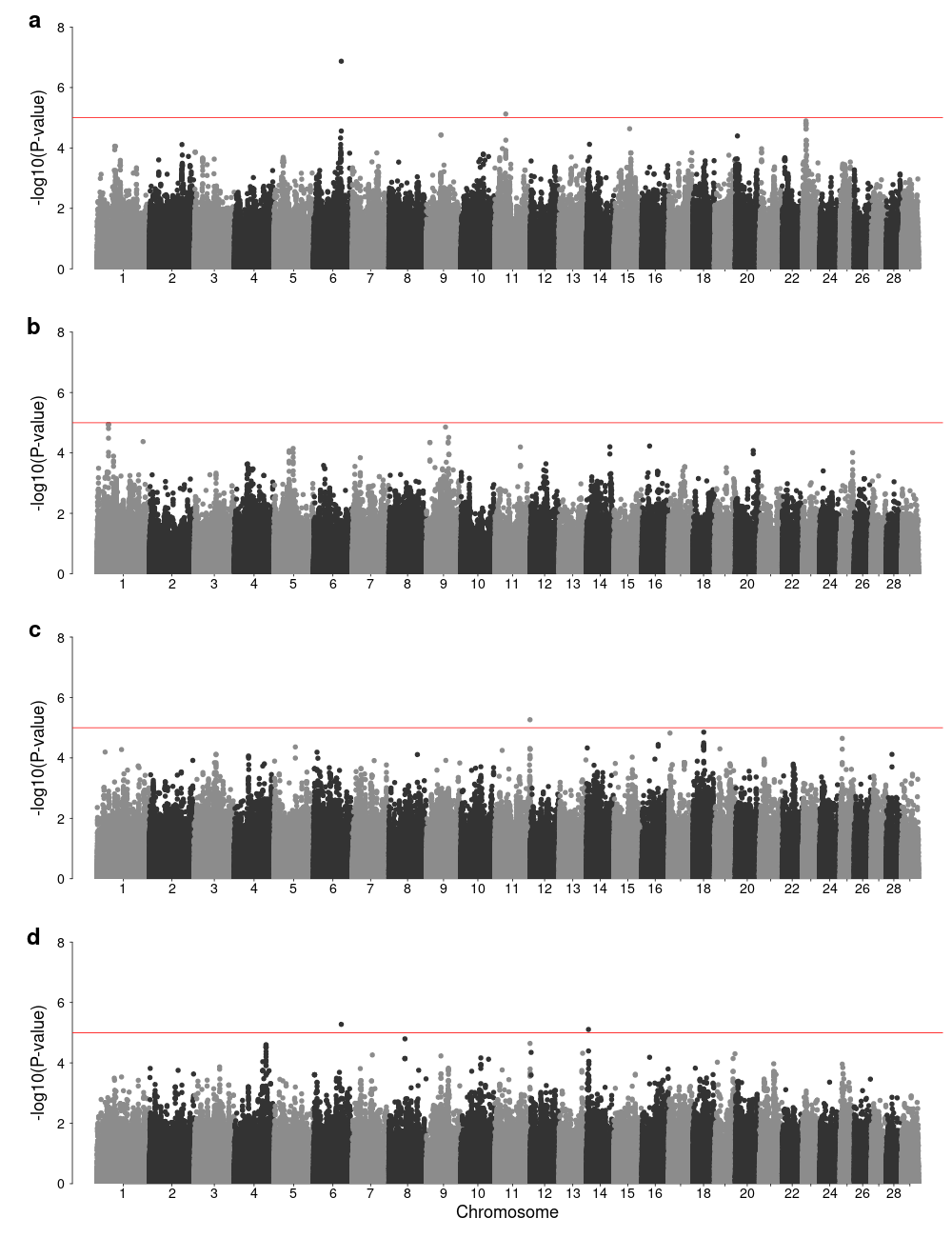
Figure S13. Manhattan plots of genotype-by-environment genome-wide association analysis using Upper Midwest & Northeast ecoregion as environmental factor for birth weight (a), weaning weight (b), yearling weight (c), and using multivariate analysis (d). Horizontal red line indicates a significant threshold (*P* < 1e-5).


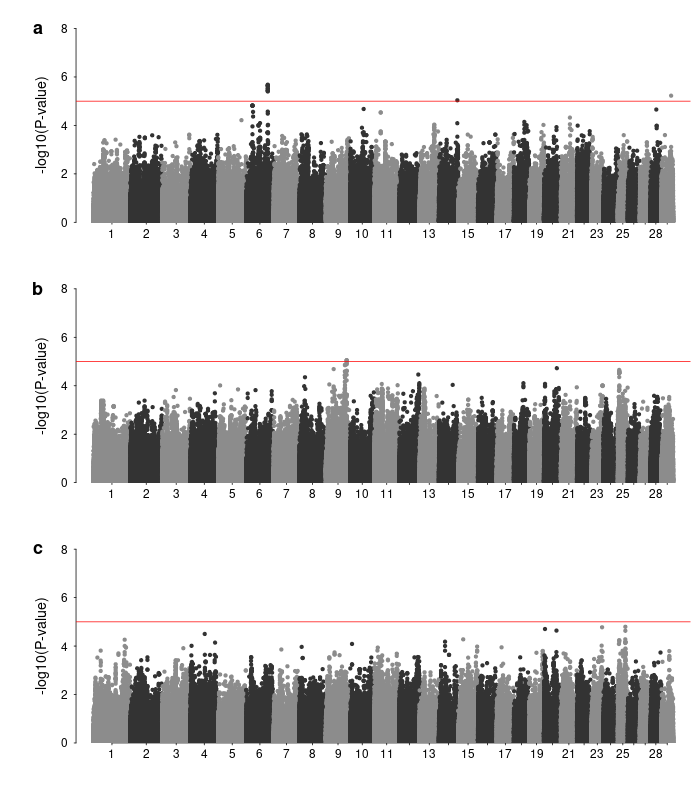
Figure S14. Manhattan plots of genotype-by-environment genome-wide association analysis using Desert & Arid Prairie ecoregion as environmental factor for birth weight (a), weaning weight (b), yearling weight (c). Horizontal red line indicates a significant threshold (*P* < 1e-5).


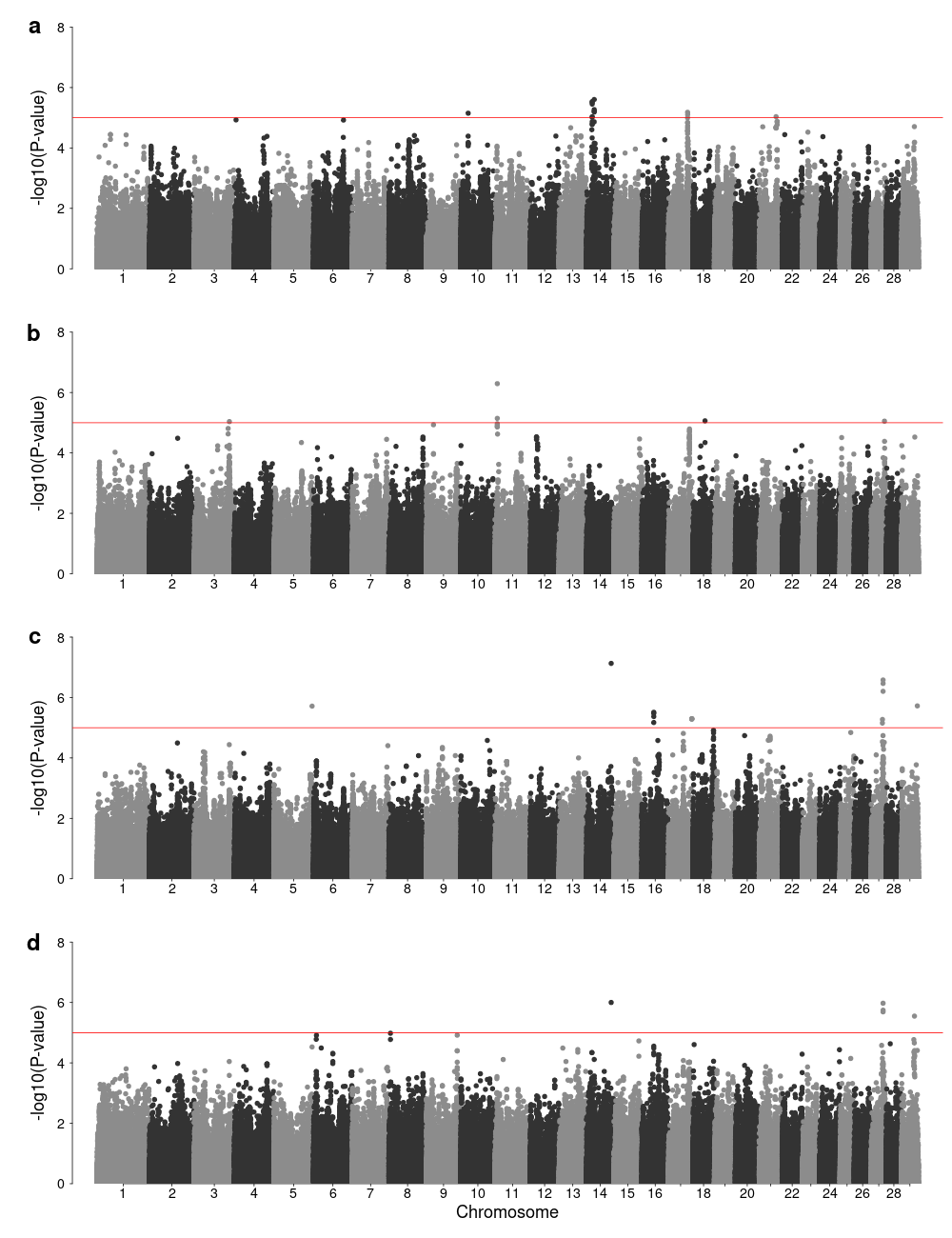
Figure S15. Manhattan plots of variance-heterogeneity genome-wide association analysis using residuals accounting for only additive effects for birth weight (a), weaning weight (b), yearling weight (c), and using multivariate analysis (d). Horizontal red line indicates a significant threshold (*P* < 1e-5).


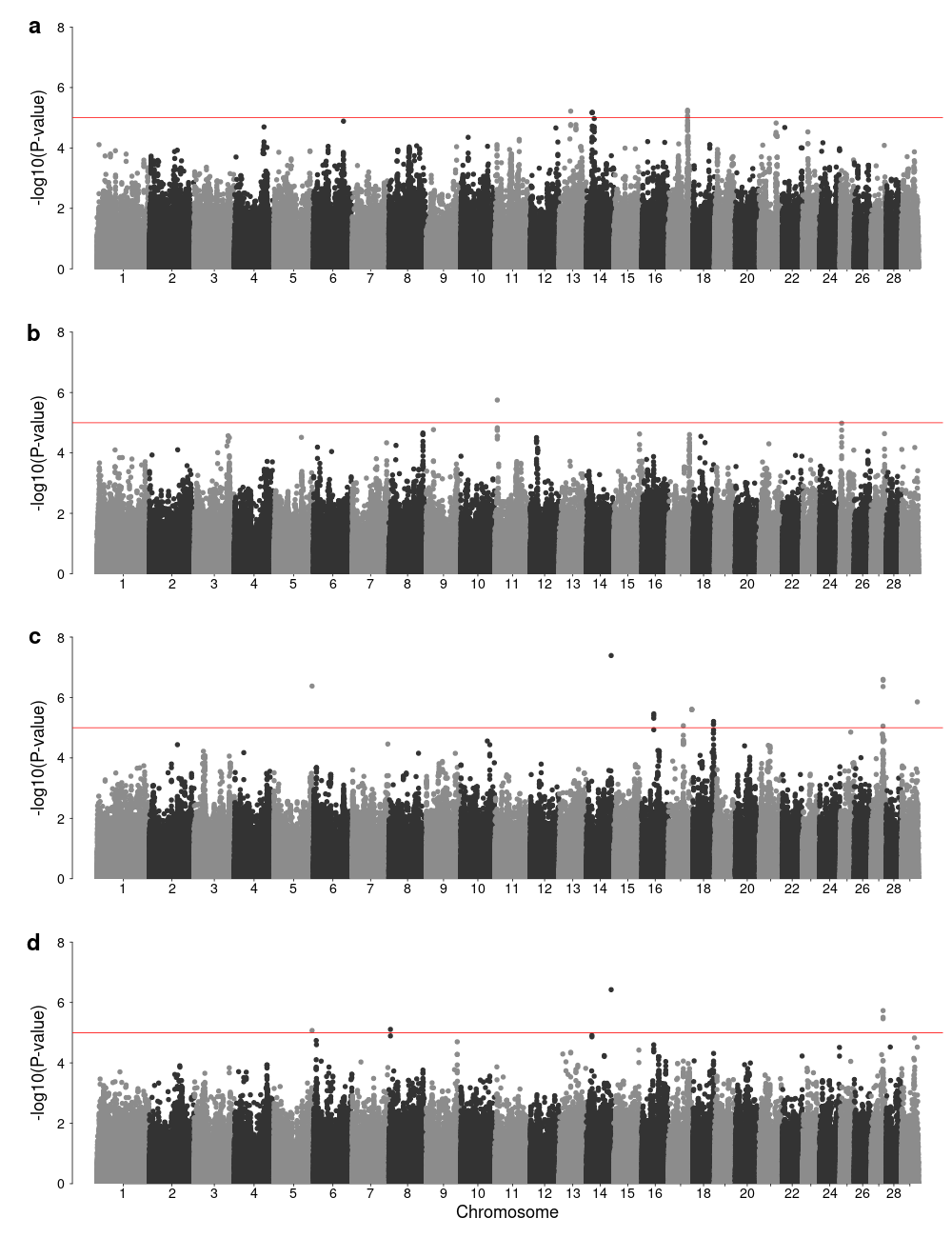
Figure S16. Manhattan plots of variance-heterogeneity genome-wide association analysis using residuals accounting for additive, dominance and epistatic effects for birth weight (a), weaning weight (b), yearling weight (c), and using multivariate analysis (d). Horizontal red line indicates a significant threshold (*P* < 1e-5).


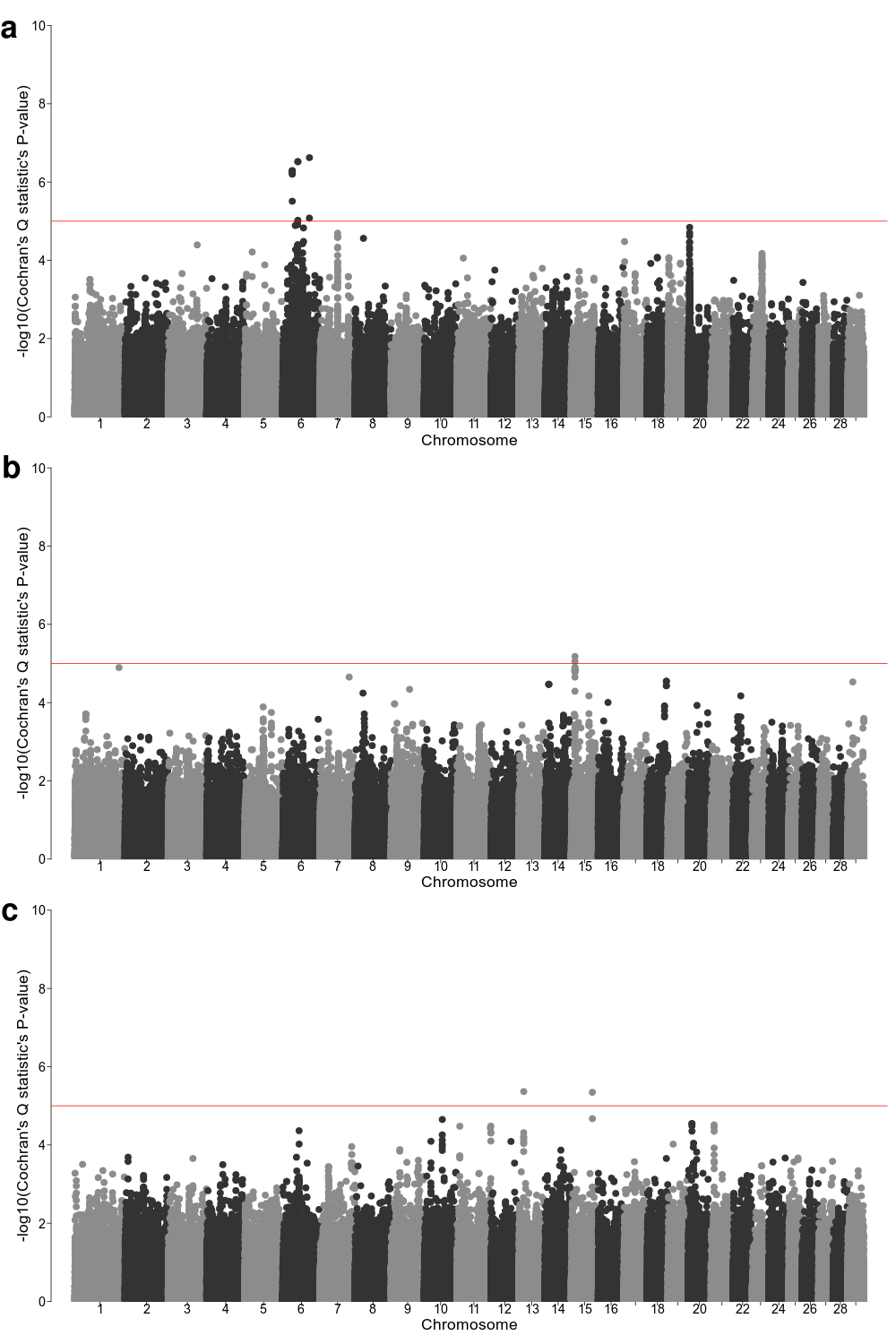
Figure S17. Meta-analysis of ecoregion-specific genome-wide association analysis. (a) Manhattan plot of Cochran's Q statistic's *P*-value for birth weight. (b) Manhattan plot of Cochran's Q statistic's *P*-value for weaning weight. (c) Manhattan plot of Cochran's Q statistic's *P*-value for yearling weight. Horizontal red line indicates a significant threshold (*P* < 1e-5).


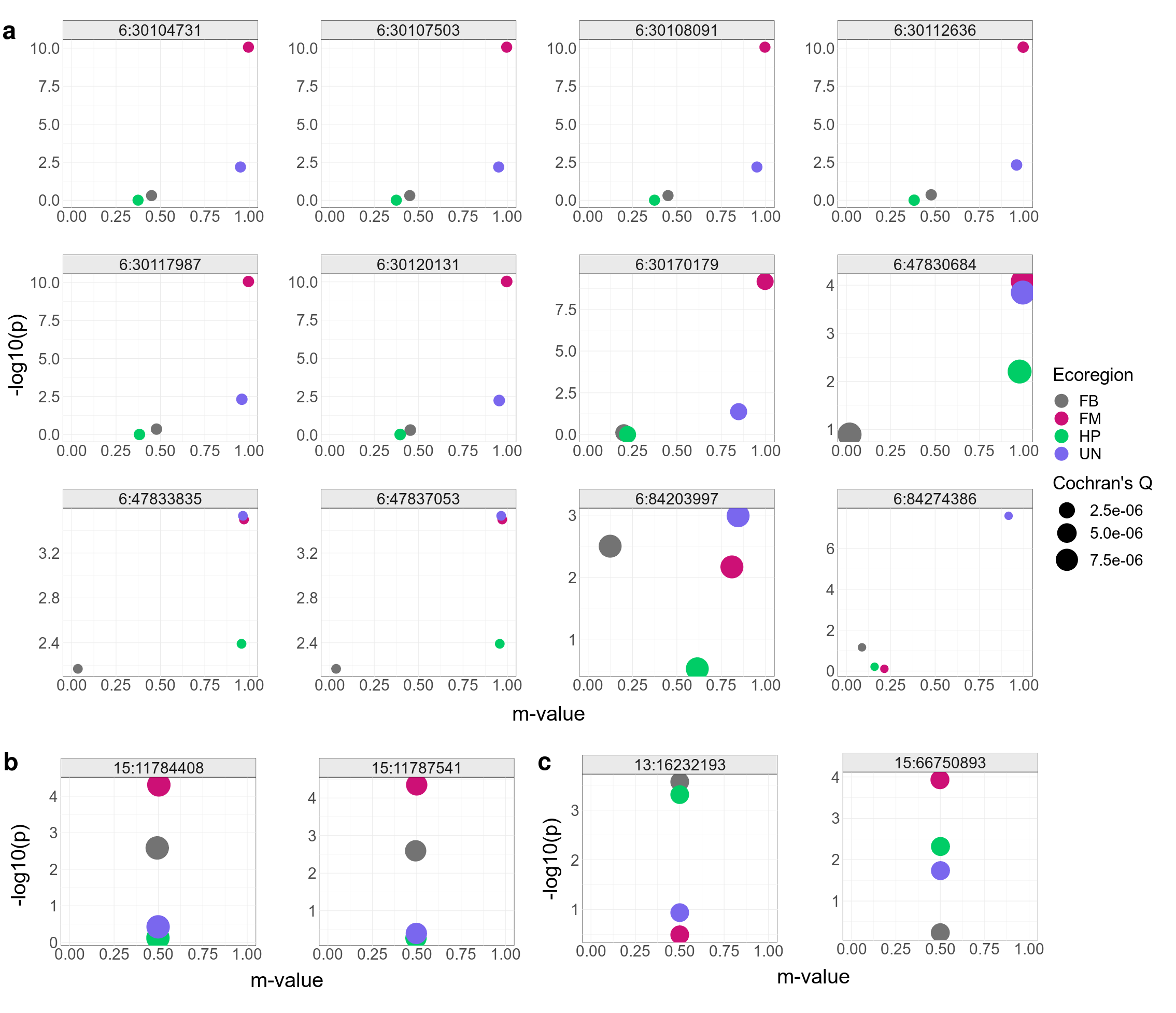
**Figure S18.** PM-plot (ecoregion-specific *P*-value and the posterior probability of an effect) from meta-analysis of ecoregion-specific GWAA for birth weight (**a**), weaning weight (**b**), and yearling weight (**c**). Points are colored by ecoregion and sized based on Cochran's Q statistic's *P*-value. United States ecoregions were represented as Fescue Belt (FB), Forested Mountains (FM), High Plains (HP), and Upper Midwest & Northeast (UN).


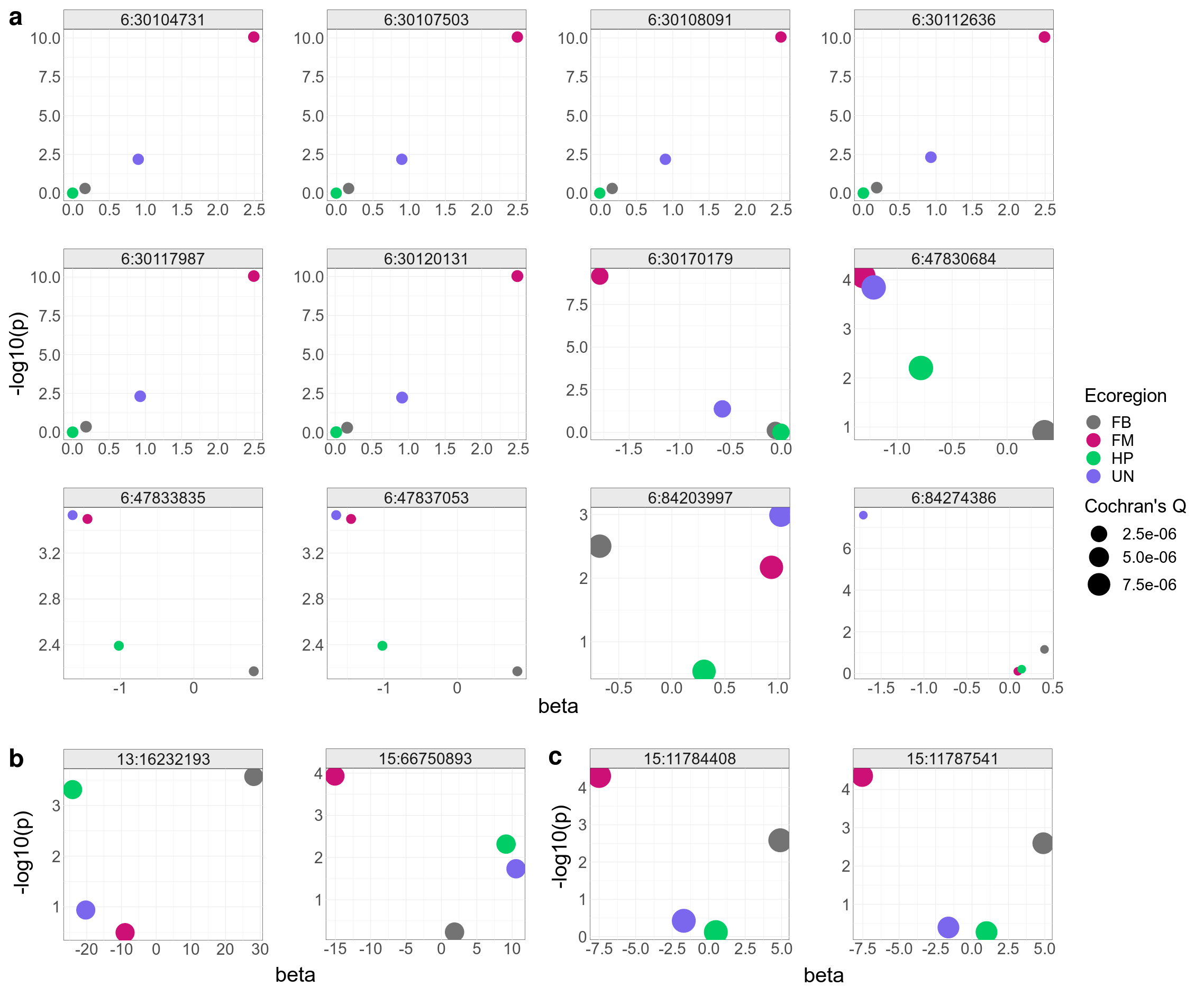
**Figure 19.** PB-plot (ecoregion-specific *P*-value and the effect size) from meta-analysis of ecoregion-specific GWAA for birth weight (**a**), weaning weight (**b**), and yearling weight (**c**). Points are colored by ecoregion and sized based on Cochran's Q statistic's *P*-value. United States ecoregions were represented as Fescue Belt (FB), Forested Mountains (FM), High Plains (HP), and Upper Midwest & Northeast (UN).


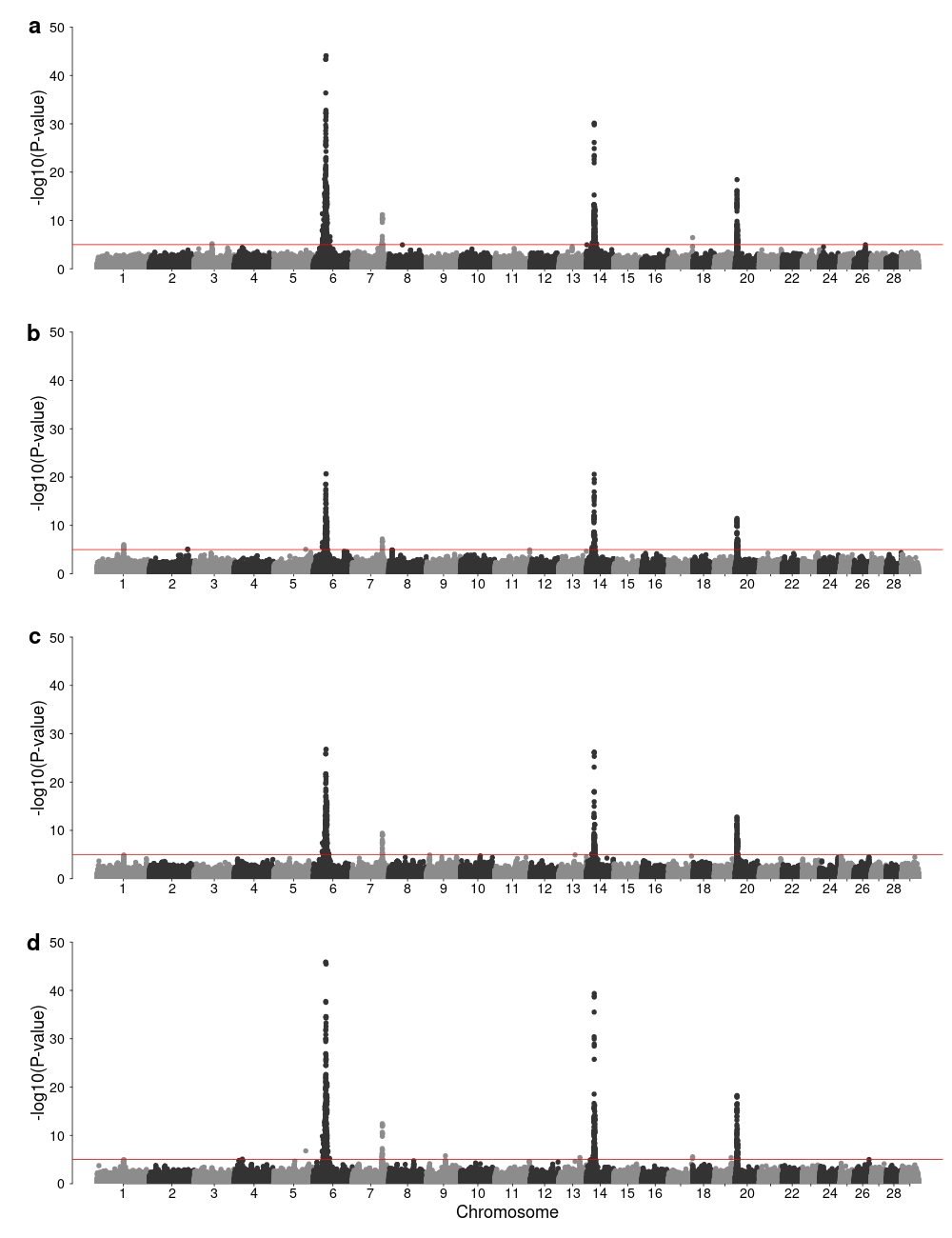
Figure S20. Manhattan plots of genome-wide association analysis for birth weight (a), weaning weight (b), yearling weight (c), and using multivariate analysis (d). Horizontal red line indicates a significant threshold (*P* < 1e-5).


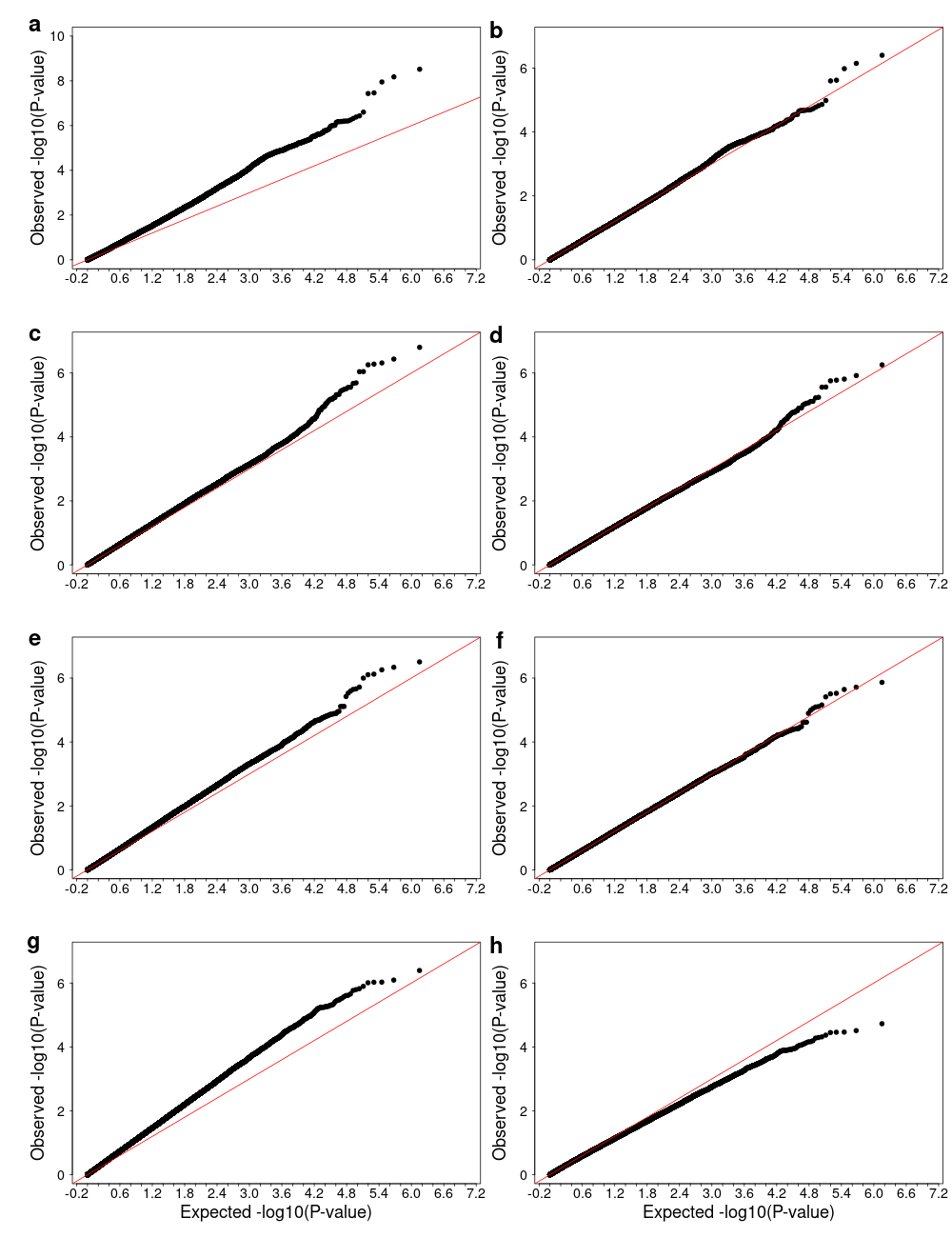
Figure S21. QQ plots of genotype-by-environment genome-wide association analysis using elevation as environmental variable for univariate birth weight before adjustment for genomic control (a), birth weight after adjustment for genomic control (b), weaning weight before adjustment for genomic control (c), weaning weight after adjustment for genomic control (d), yearling weight before adjustment for genomic control (e), yearling weight after adjustment for genomic control (f), multivariate analysis before adjustment for genomic control (g), multivariate analysis after adjustment for genomic control (h).


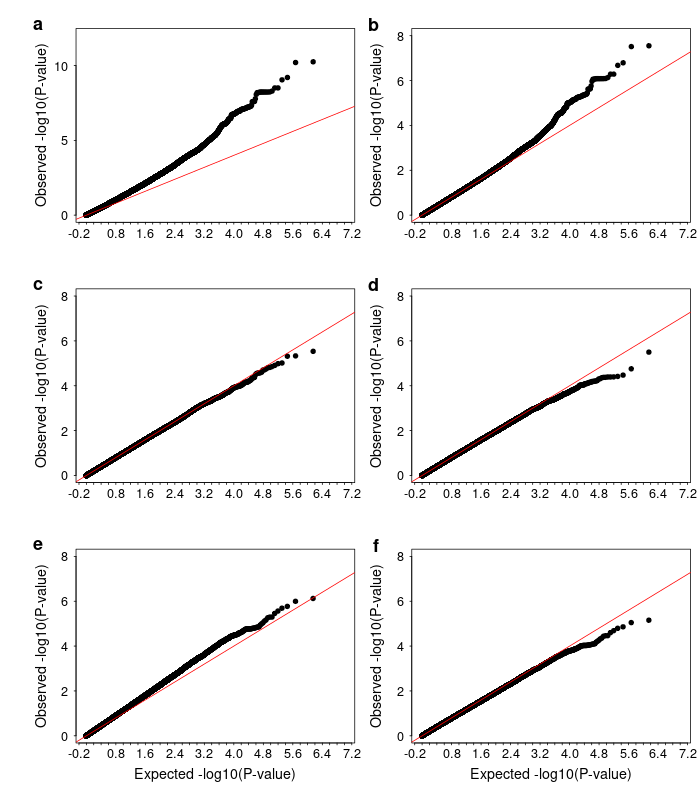
Figure S22. QQ plots of genotype-by-environment genome-wide association analysis using precipitation as environmental variable for univariate birth weight before adjustment for genomic control (a), birth weight after adjustment for genomic control (b), weaning weight (c), yearling weight (d), multivariate analysis before adjustment for genomic control (e), multivariate analysis after adjustment for genomic control (f).


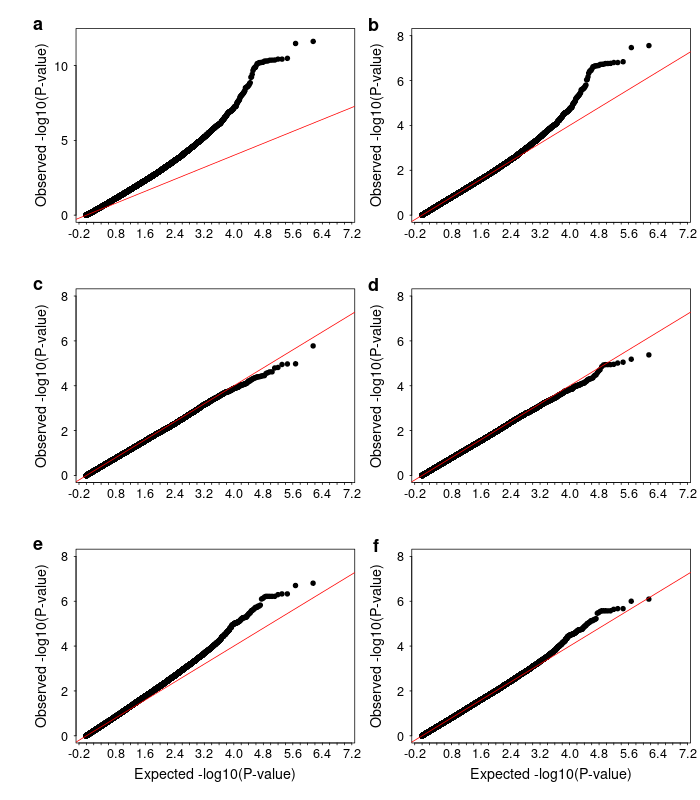
Figure S23. QQ plots of genotype-by-environment genome-wide association analysis using mean temperature as environmental variable for univariate birth weight before adjustment for genomic control (a), birth weight after adjustment for genomic control (b), weaning weight (c), yearling weight (d), multivariate analysis before adjustment for genomic control (e), multivariate analysis after adjustment for genomic control (f).


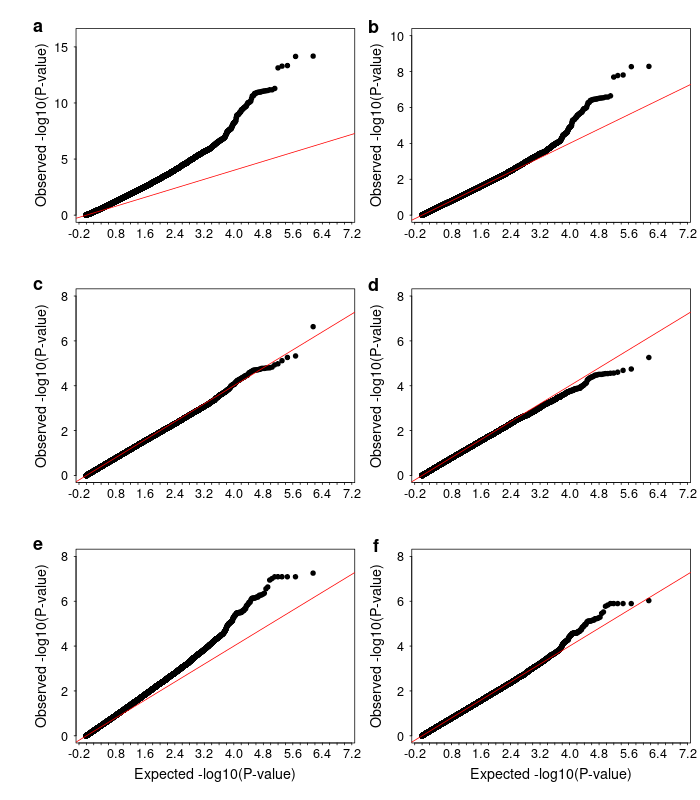
Figure S24. QQ plots of genotype-by-environment genome-wide association analysis using mean dew point temperature as environmental variable for univariate birth weight before adjustment for genomic control (a), birth weight after adjustment for genomic control (b), weaning weight (c), yearling weight (d), multivariate analysis before adjustment for genomic control (e), multivariate analysis after adjustment for genomic control (f).


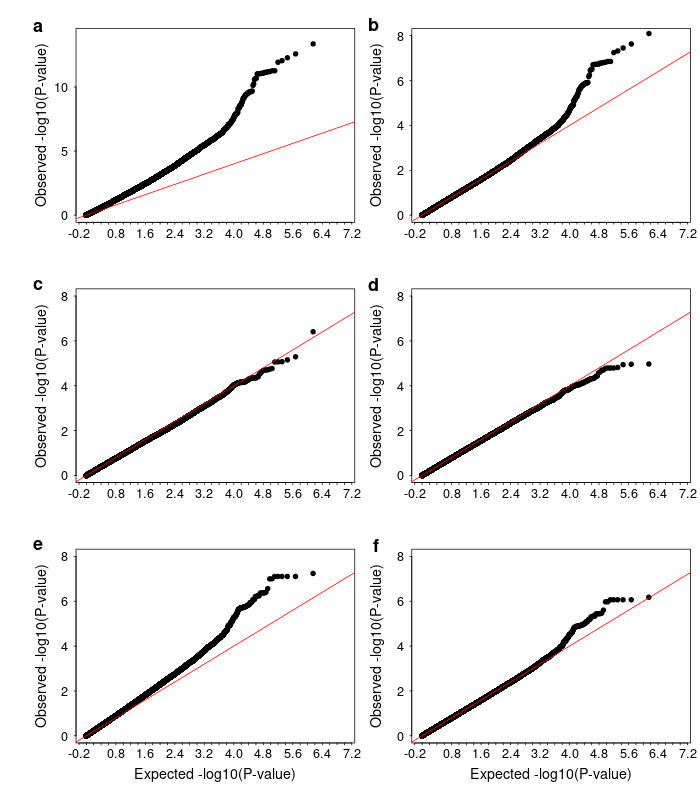
Figure S25. QQ plots of genotype-by-environment genome-wide association analysis using minimum temperature as environmental variable for univariate birth weight before adjustment for genomic control (a), birth weight after adjustment for genomic control (b), weaning weight (c), yearling weight (d), multivariate analysis before adjustment for genomic control (e), multivariate analysis after adjustment for genomic control (f).


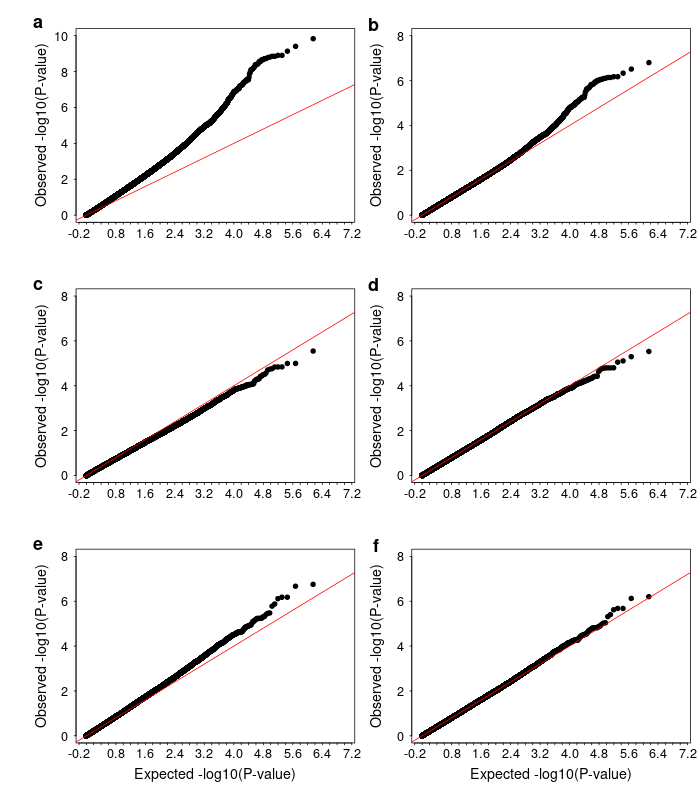
Figure S26. QQ plots of genotype-by-environment genome-wide association analysis using maximum temperature as environmental variable for univariate birth weight before adjustment for genomic control (a), birth weight after adjustment for genomic control (b), weaning weight (c), yearling weight (d), multivariate analysis before adjustment for genomic control (e), multivariate analysis after adjustment for genomic control (f).


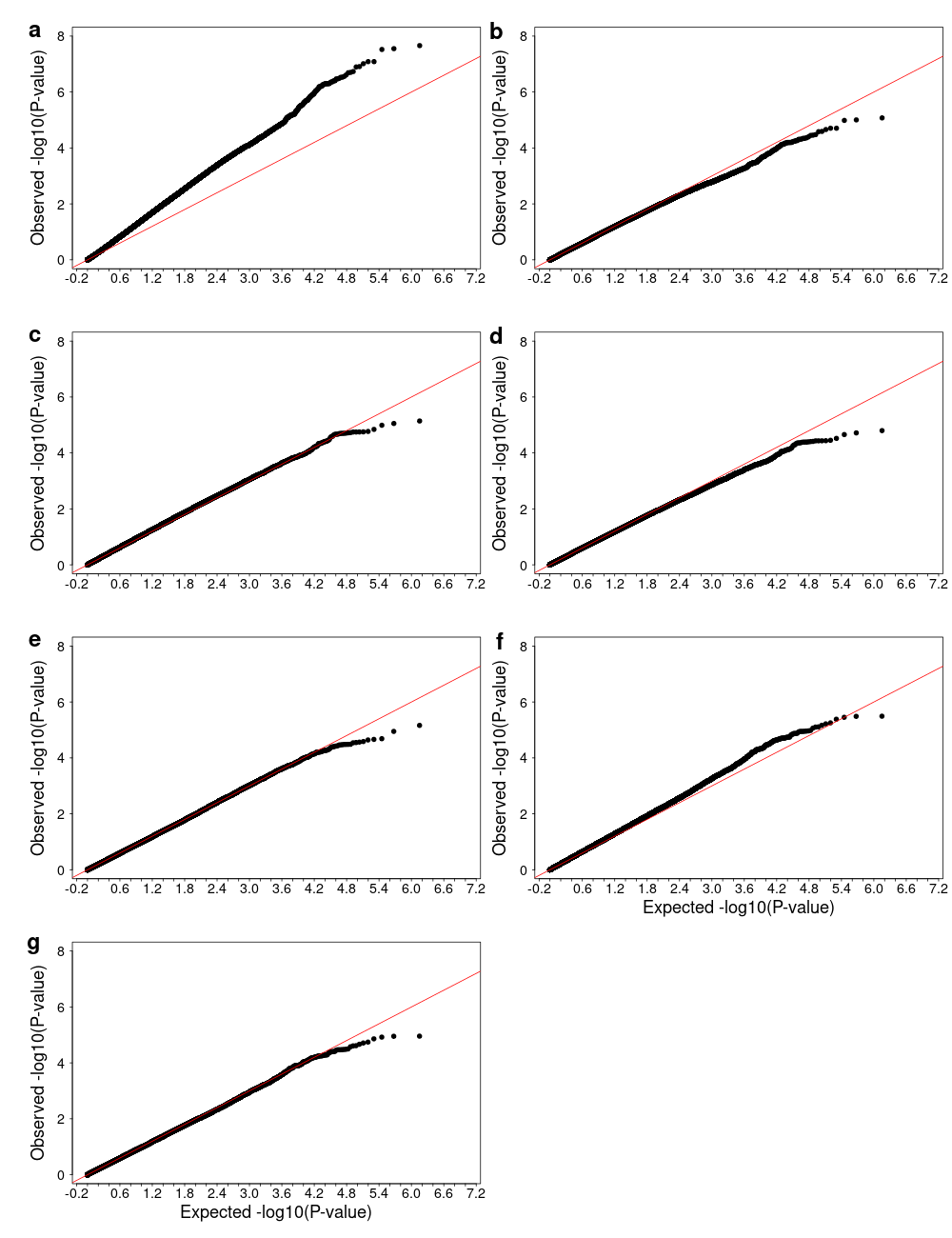
Figure S27. QQ plots of genotype-by-environment genome-wide association analysis using minimum vapor pressure deficit as environmental variable for univariate birth weight before adjustment for genomic control (a), birth weight after adjustment for genomic control (b), weaning weight before adjustment for genomic control (c), weaning weight after adjustment for genomic control (d), yearling weight (e), multivariate analysis before adjustment for genomic control (f), multivariate analysis after adjustment for genomic control (g).


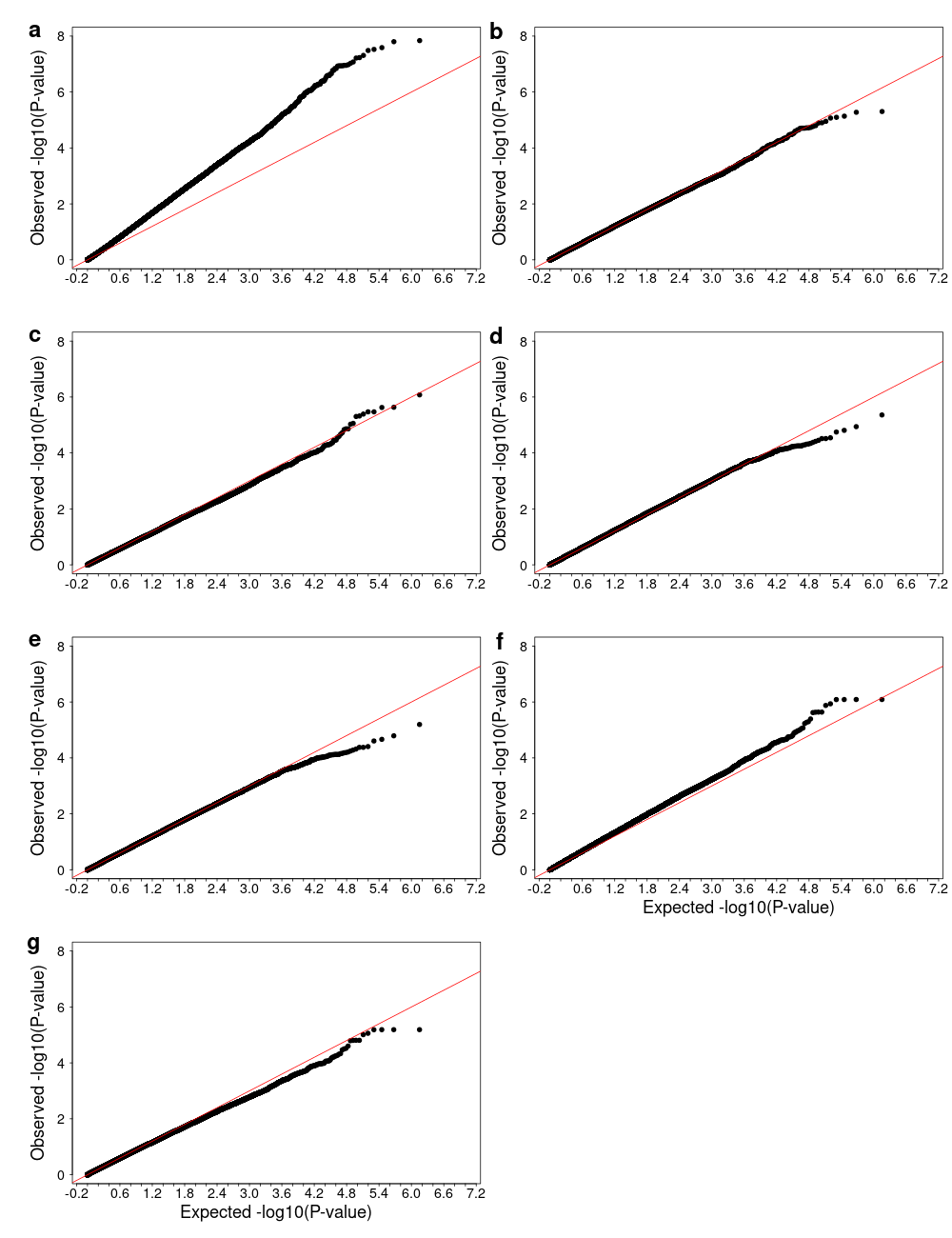
Figure S28. QQ plots of genotype-by-environment genome-wide association analysis using maximum vapor pressure deficit as environmental variable for univariate birth weight before adjustment for genomic control (a), birth weight after adjustment for genomic control (b), weaning weight (c), yearling weight before adjustment for genomic control (d), yearling weight after adjustment for genomic control (e), multivariate analysis before adjustment for genomic control (f), multivariate analysis after adjustment for genomic control (g).


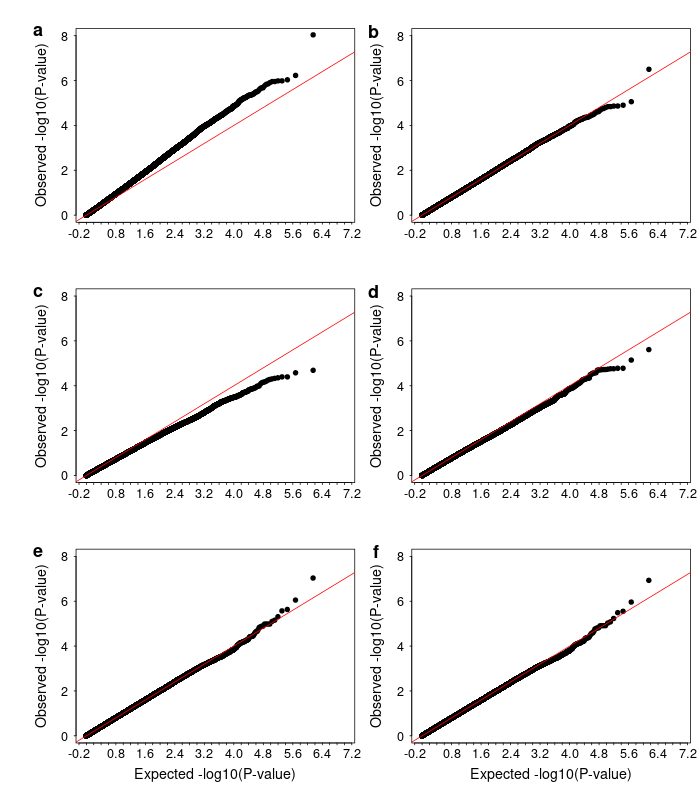
Figure S29. QQ plots of genotype-by-environment genome-wide association analysis using Southeast ecoregion as environmental variable for univariate birth weight before adjustment for genomic control (a), birth weight after adjustment for genomic control (b), weaning weight (c), yearling weight (d), multivariate analysis before adjustment for genomic control (e), multivariate analysis after adjustment for genomic control (f).


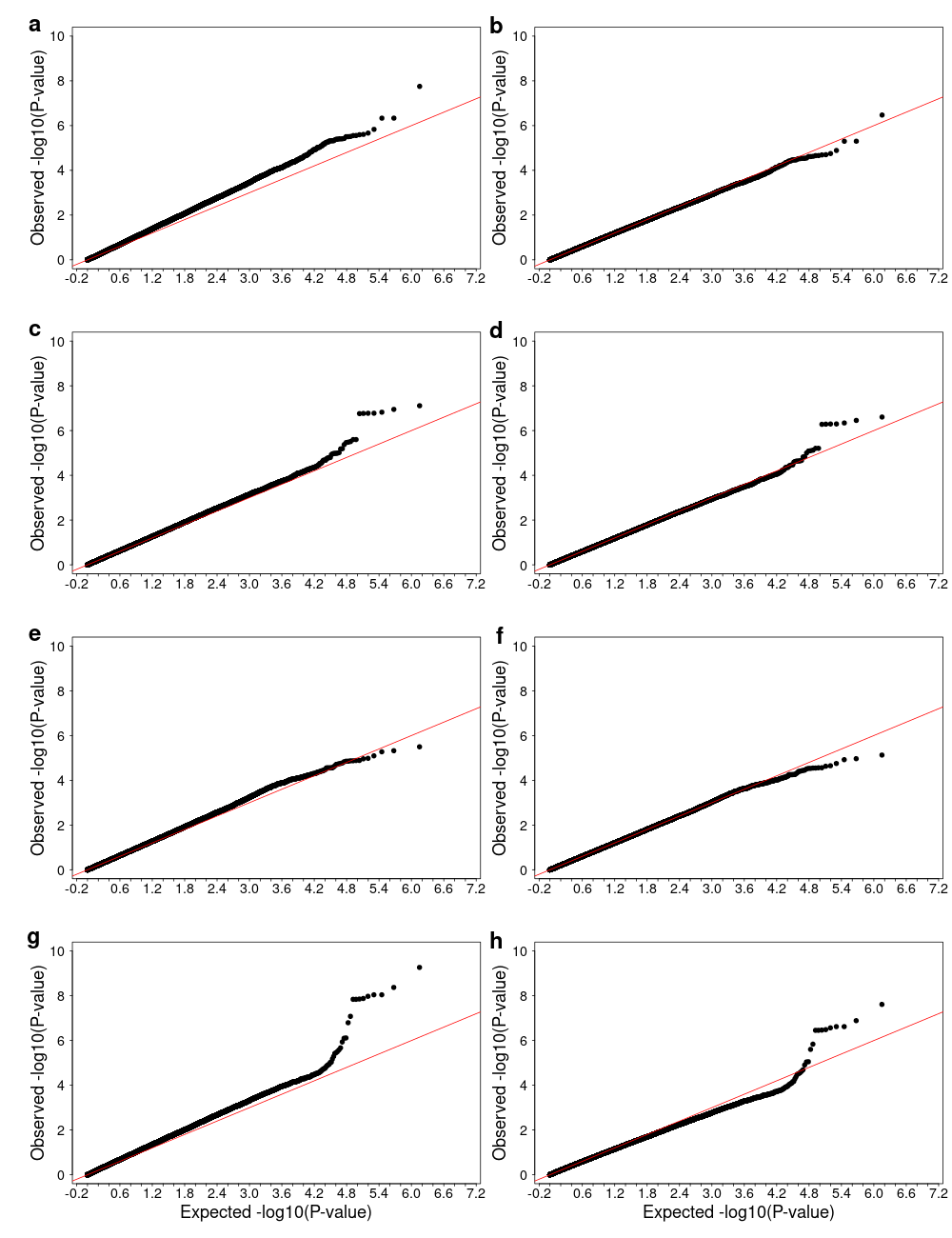
Figure S30. QQ plots of genotype-by-environment genome-wide association analysis using High Plains ecoregion as environmental variable for univariate birth weight before adjustment for genomic control (a), birth weight after adjustment for genomic control (b), weaning weight before adjustment for genomic control (c), weaning weight after adjustment for genomic control (d), yearling weight before adjustment for genomic control (e), yearling weight after adjustment for genomic control (f), multivariate analysis before adjustment for genomic control (g), multivariate analysis after adjustment for genomic control (h).


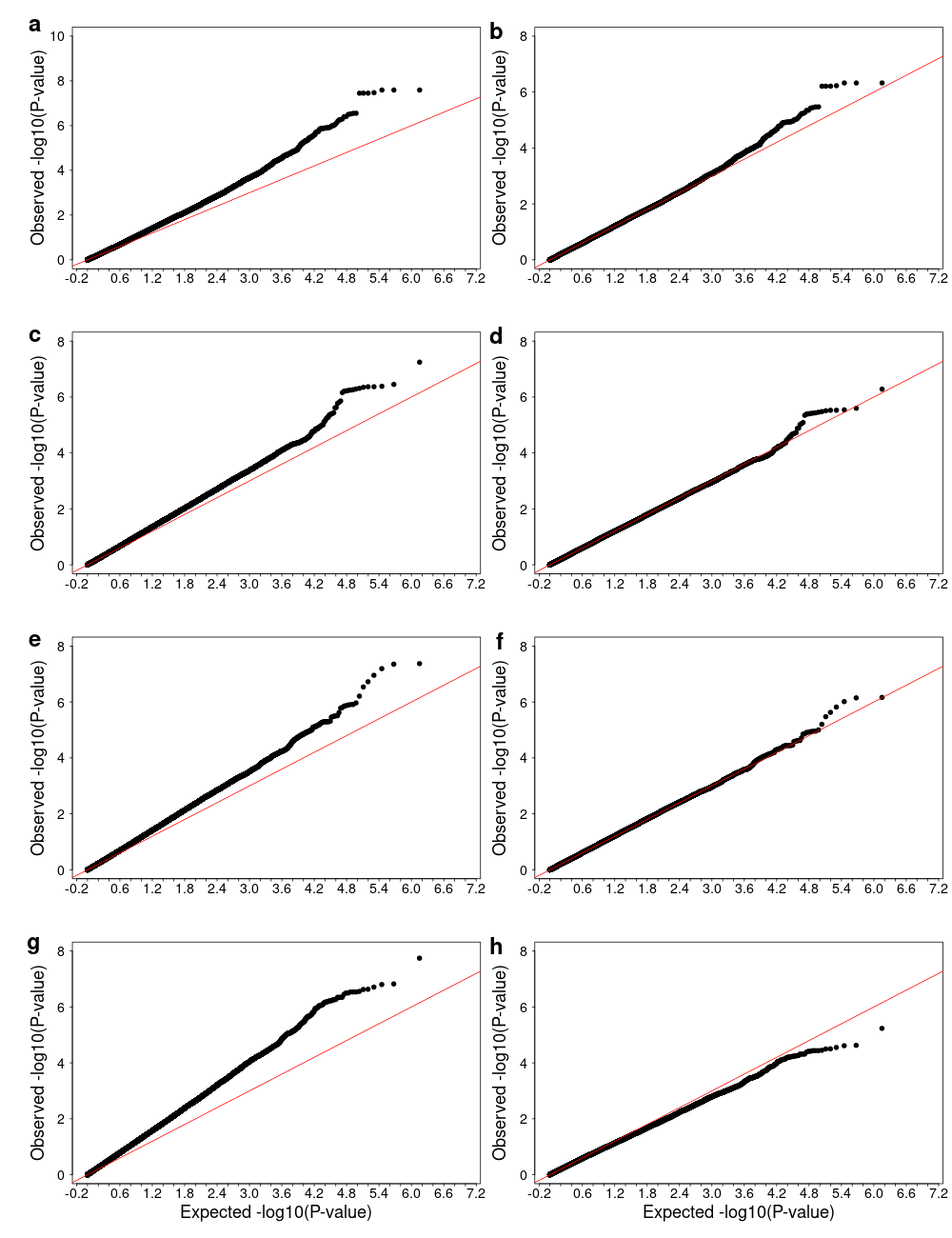
Figure S31. QQ plots of genotype-by-environment genome-wide association analysis using Forested Mountains ecoregion as environmental variable for univariate birth weight before adjustment for genomic control (a), birth weight after adjustment for genomic control (b), weaning weight before adjustment for genomic control (c), weaning weight after adjustment for genomic control (d), yearling weight before adjustment for genomic control (e), yearling weight after adjustment for genomic control (f), multivariate analysis before adjustment for genomic control (g), multivariate analysis after adjustment for genomic control (h).


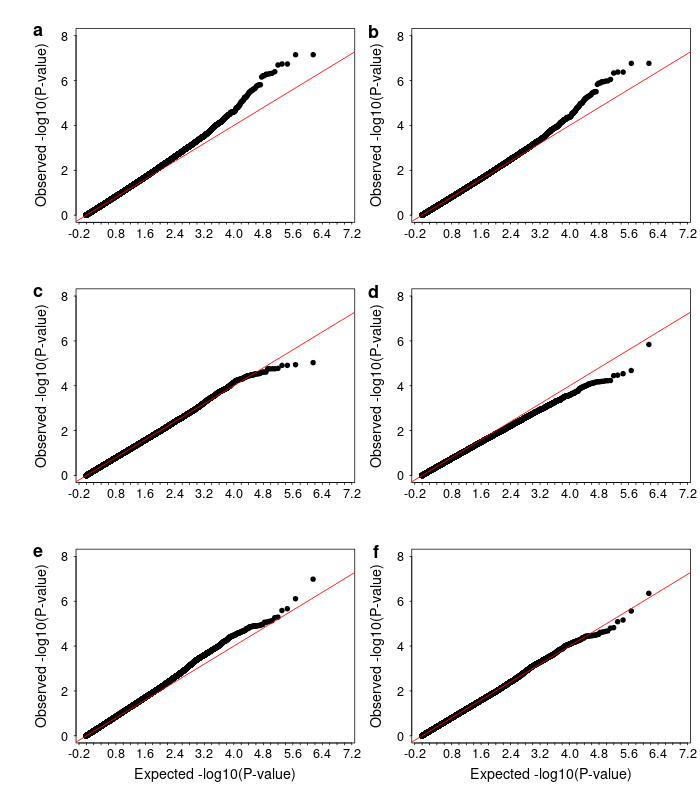
Figure S32. QQ plots of genotype-by-environment genome-wide association analysis using Fescue Belt ecoregion as environmental variable for univariate birth weight before adjustment for genomic control (a), birth weight after adjustment for genomic control (b), weaning weight (c), yearling weight (d), multivariate analysis before adjustment for genomic control (e), multivariate analysis after adjustment for genomic control (f).


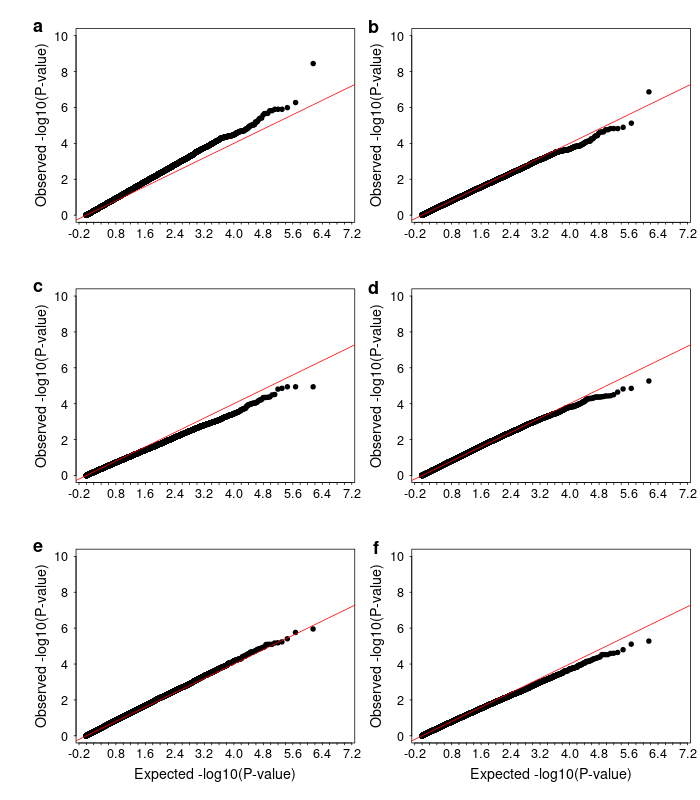
**Figure S33.** QQ plots of genotype-by-environment genome-wide association analysis using Upper Midwest & Northeast ecoregion as environmental variable for univariate birth weight before adjustment for genomic control (**a**), birth weight after adjustment for genomic control (**b**), weaning weight (**c**), yearling weight (**d**), multivariate analysis before adjustment for genomic control (**e**), multivariate analysis after adjustment for genomic control (**f**).


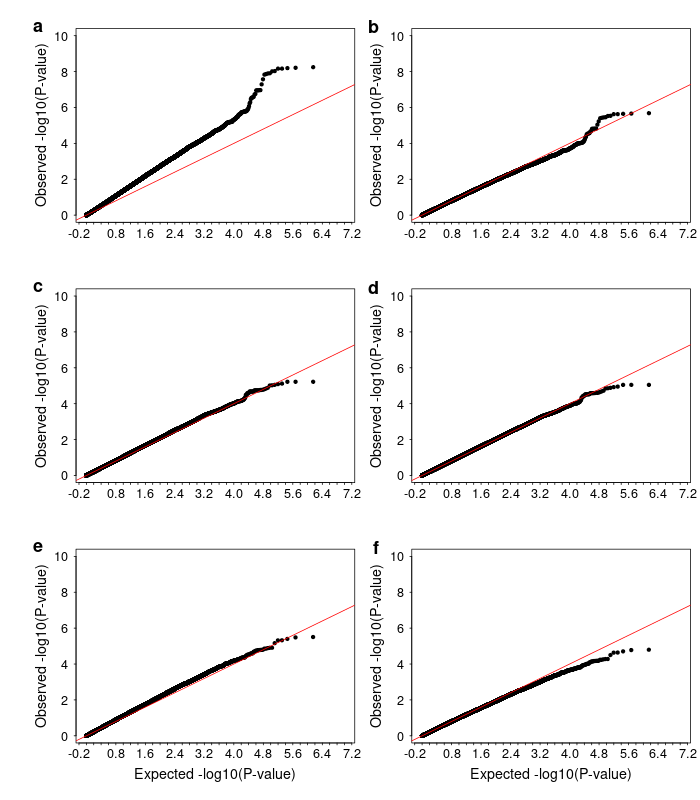
**Figure S34.** QQ plots of genotype-by-environment genome-wide association analysis using Desert & Arid Plains ecoregion as environmental variable for univariate birth weight before adjustment for genomic control (**a**), birth weight after adjustment for genomic control (**b**), weaning weight before adjustment for genomic control (**c**), weaning weight after adjustment for genomic control (**d**), yearling weight before adjustment for genomic control (**e**), yearling weight after adjustment for genomic control (**f**).


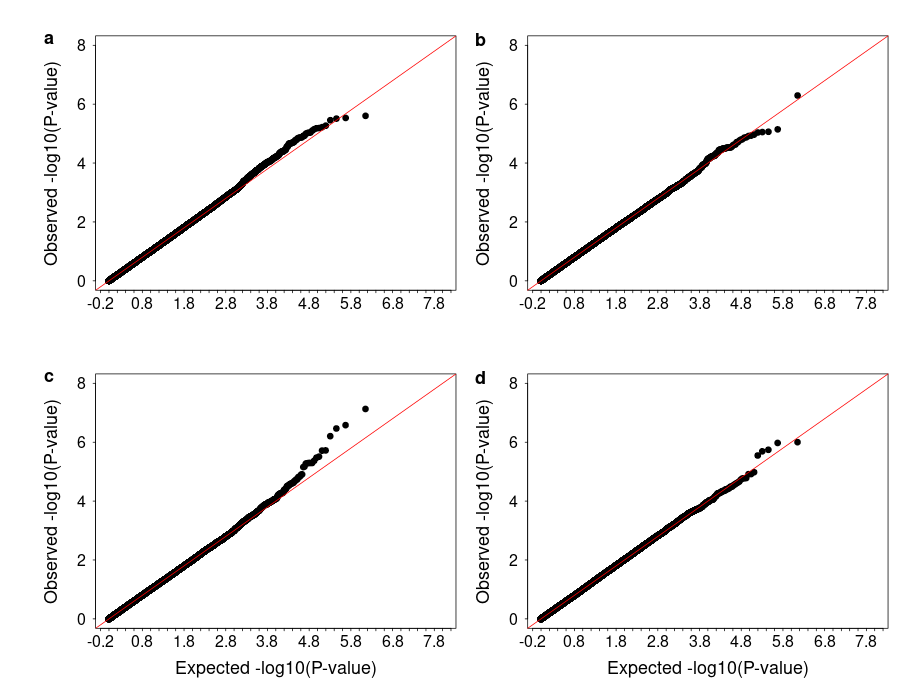
**Figure S35.** QQ plots of variance-heterogeneity genome-wide association analysis using residuals accounting for only additive effects for birth weight (**a**), weaning weight (**b**), yearling weight (**c**), and using multivariate analysis (**d**).


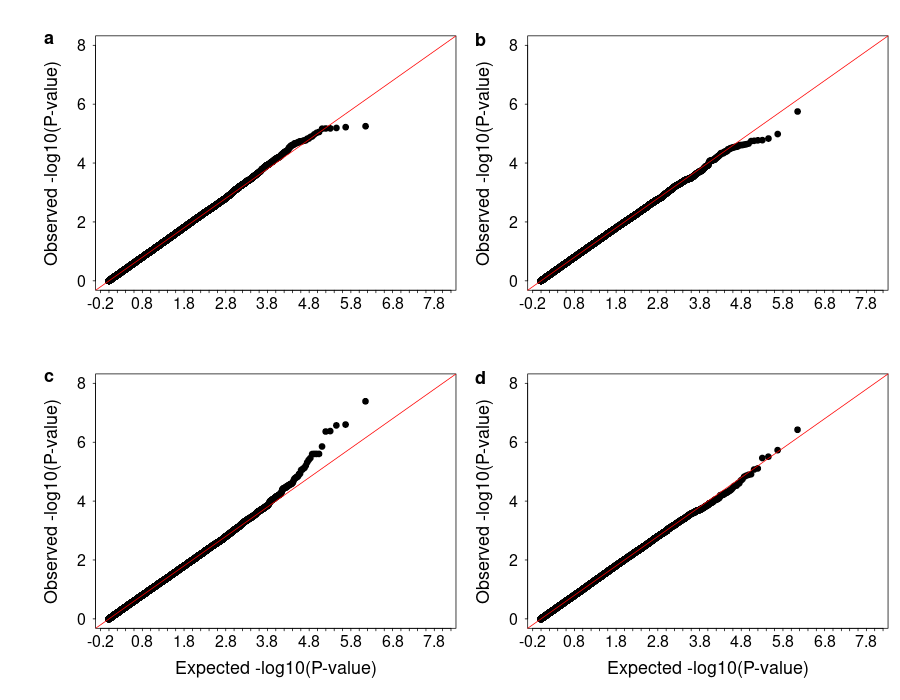
**Figure S36.** QQ plots of variance-heterogeneity genome-wide association analysis using residuals accounting for additive, dominance and epistatic effects for birth weight (**a**), weaning weight (**b**), yearling weight (**c**), and using multivariate analysis (**d**).


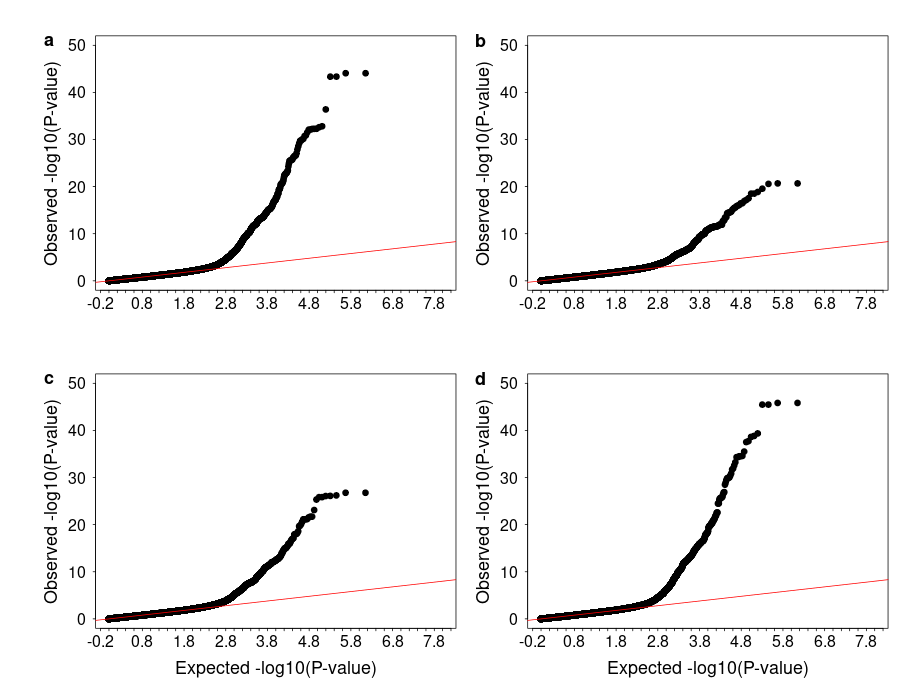
**Figure S37.** QQ plots of genome-wide association analysis for birth weight (**a**), weaning weight (**b**), yearling weight (**c**), and using multivariate analysis (**d**).


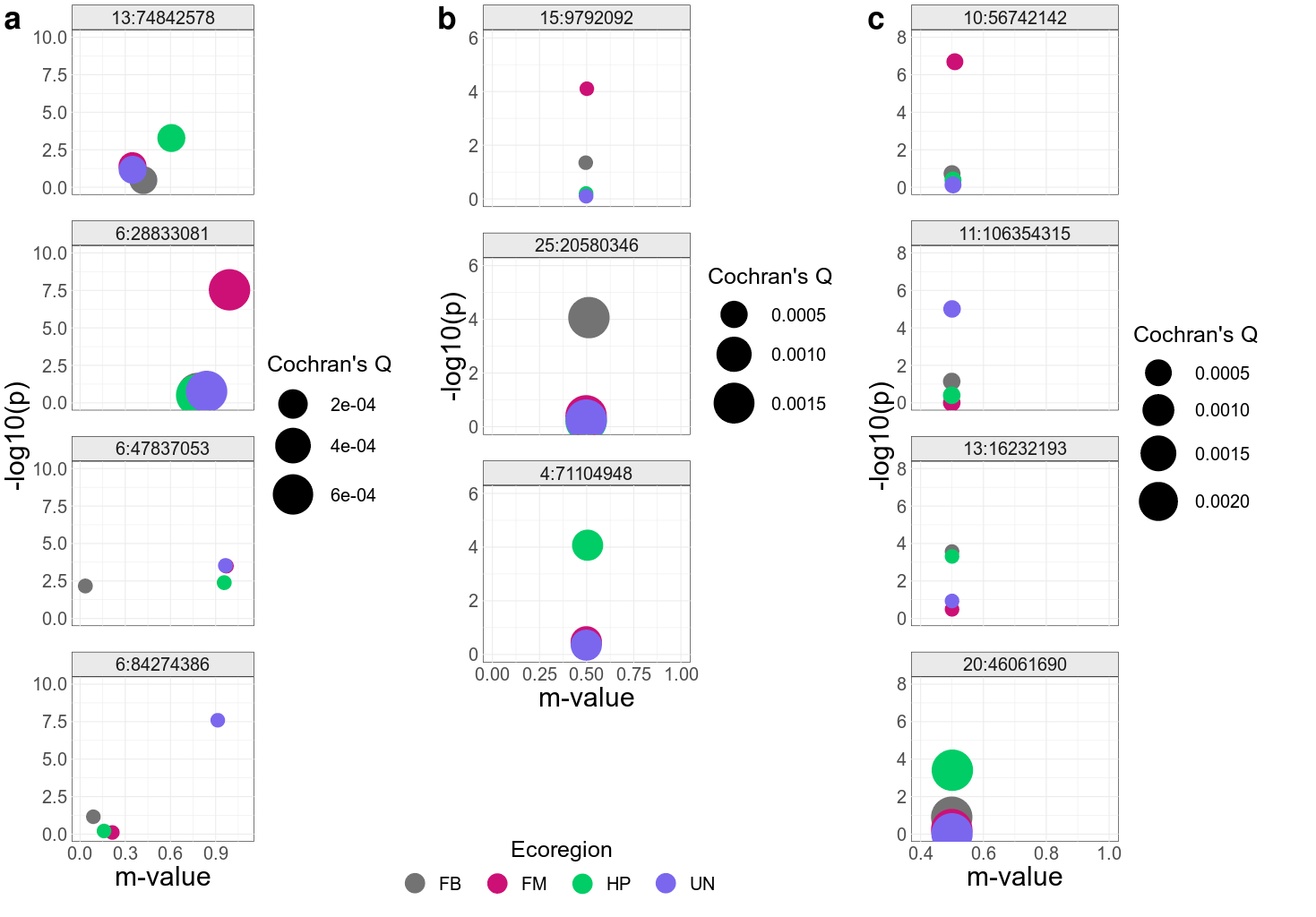
Figure S38. PM-plot (ecoregion-specific *P*-value and the posterior probability of an effect) from meta-analysis of ecoregion-specific GWAA of some of the most significant GxE SNPs identified by GxE GWAA for birth weight (a), weaning weight (b), and yearling weight (c). Points are colored by ecoregion and sized based on Cochran's Q statistic's *P*-value. United States ecoregions were represented as Fescue Belt (FB), Forested Mountains (FM), High Plains (HP), and Upper Midwest & Northeast (UN). Based on GxE GWAA results, SNPs 15:9792092, 6:28833081, 6:47837053, and 6:84274386 interact with HP, FM, FB, and UN ecoregions, respectively, and affect BW; SNPs 13:74842578, 25:30580346, and 4:71104948 interact with FM, FB, HP ecoregions, respectively, influencing WW; SNPs 10:56742142, 11:106354315, 13:16232193, and 20:46061690 interact with FM, UN, FB, and HP ecoregions, respectively, affecting YW.


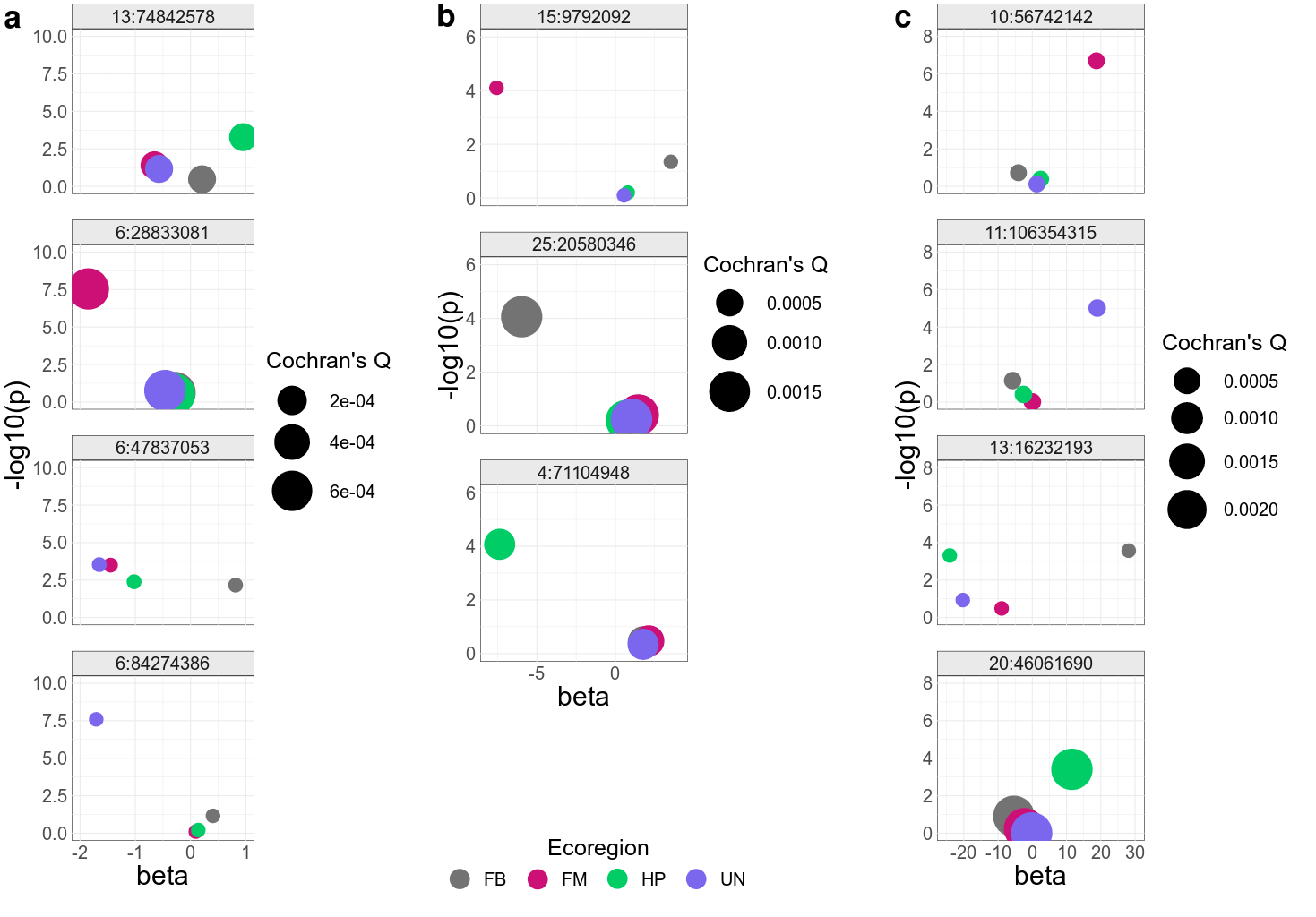
Figure S39. PB-plot (ecoregion-specific *P*-value and the effect size) from meta-analysis of ecoregion-specific GWAA of some of the most significant GxE SNPs identified by GxE GWAA for birth weight (a), weaning weight (b), and yearling weight (c). Points are colored by ecoregion and sized based on Cochran's Q statistic's *P*-value. United States ecoregions were represented as Fescue Belt (FB), Forested Mountains (FM), High Plains (HP), and Upper Midwest & Northeast (UN). Based on GxE GWAA results, SNPs 15:9792092, 6:28833081, 6:47837053, and 6:84274386 interact with HP, FM, FB, and UN ecoregions, respectively, and affect birth weight; SNPs 13:74842578, 25:30580346, and 4:71104948 interact with FM, FB, HP ecoregions, respectively, influencing weaning weight; SNPs 10:56742142, 11:106354315, 13:16232193, and 20:46061690 interact with FM, UN, FB, and HP ecoregions, respectively, affecting yearling weight.


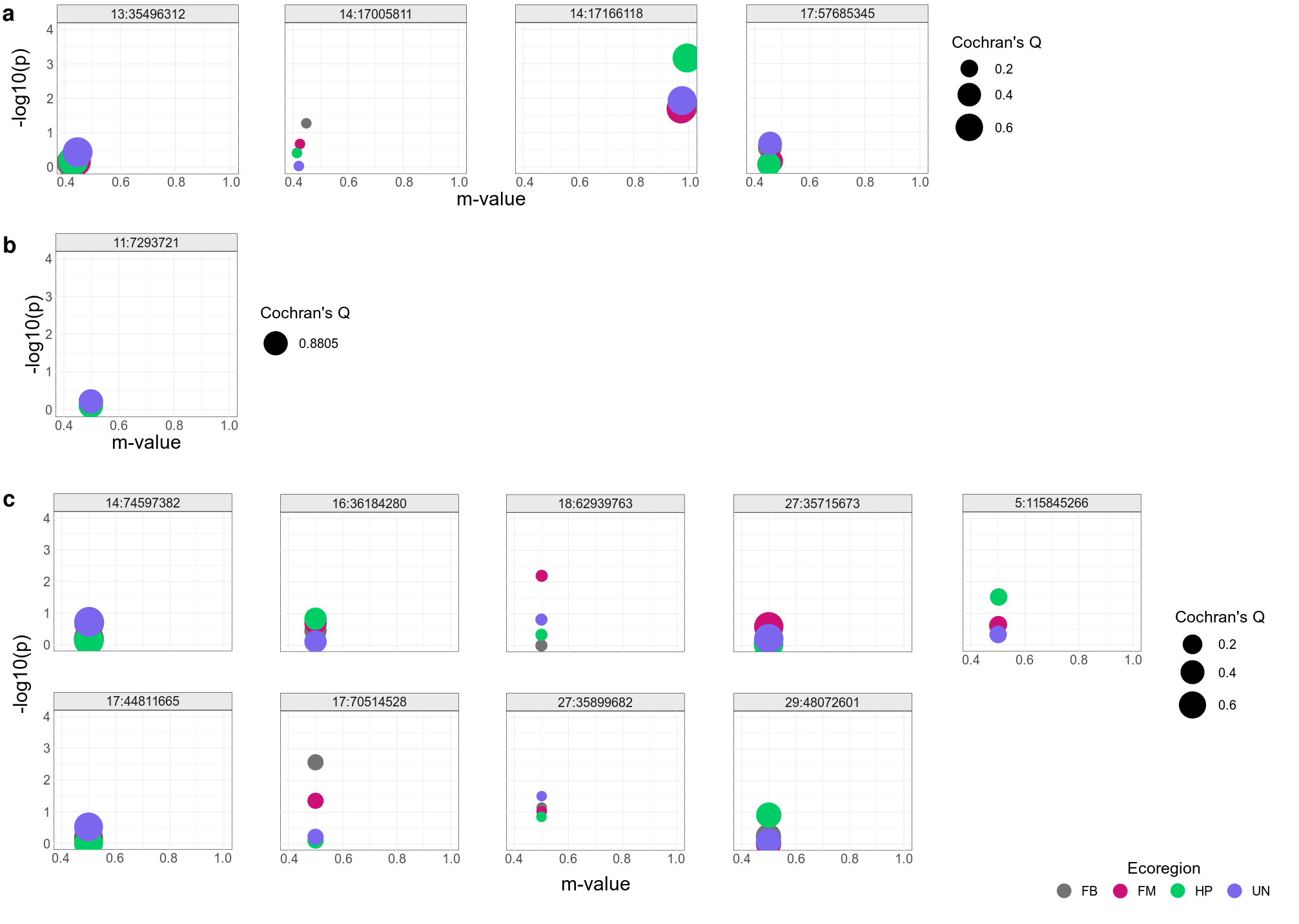
Figure S40. PM-plot (ecoregion-specific *P*-value and the posterior probability of an effect) from meta-analysis of ecoregion-specific GWAA of variance-heterogeneity SNPs identified by vGWAA for birth weight (a), weaning weight (b), and yearling weight (c). Points are colored by ecoregion and sized based on Cochran's Q statistic's *P*-value. United States ecoregions were represented as Fescue Belt (FB), Forested Mountains (FM), High Plains (HP), and Upper Midwest & Northeast (UN).


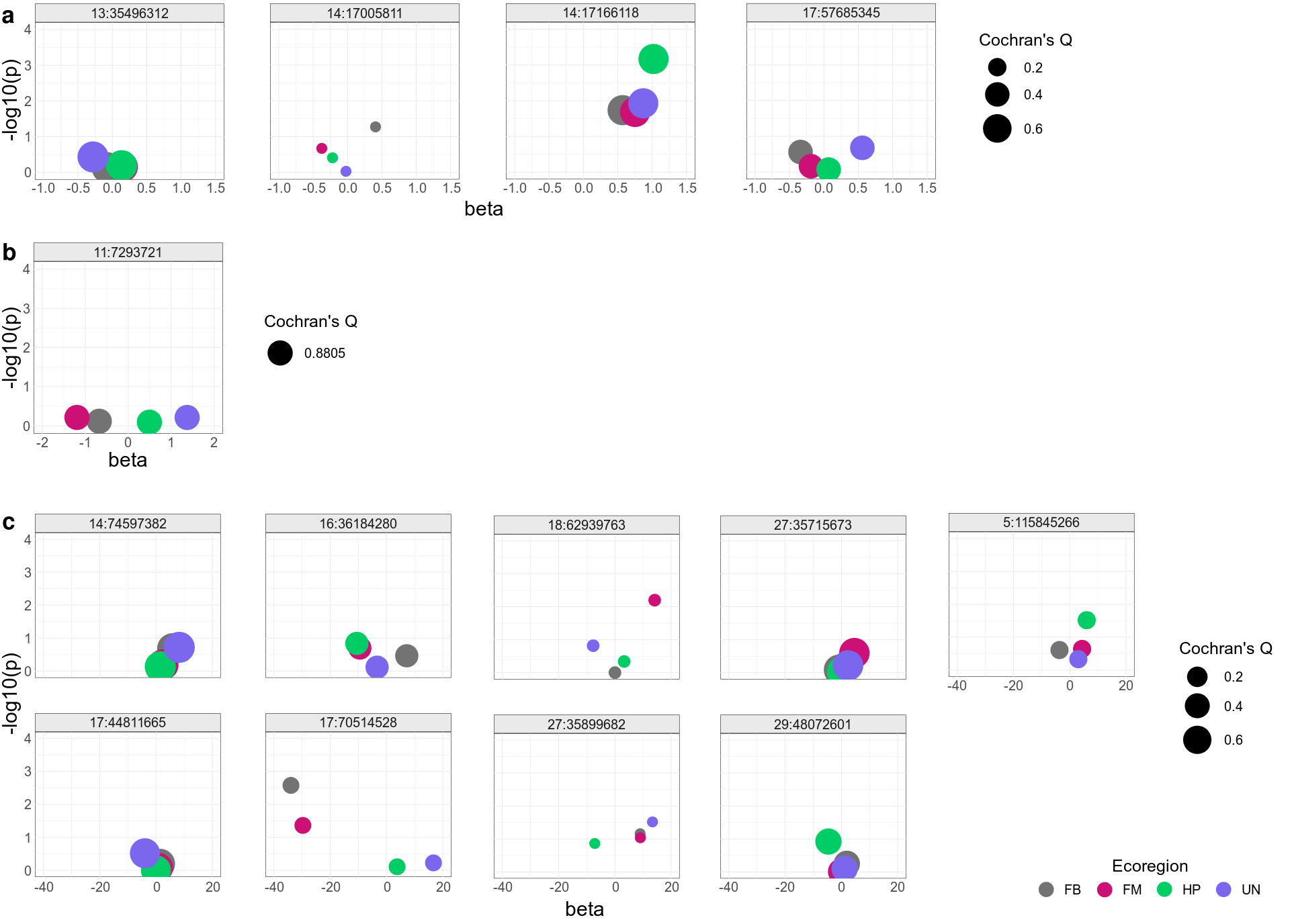
Figure S41. PB-plot (ecoregion-specific *P*-value and the effect size) from meta-analysis of ecoregion-specific GWAA of variance-heterogeneity SNPs identified by vGWAA for birth weight (a), weaning weight (b), and yearling weight (c). Points are colored by ecoregion and sized based on Cochran's Q statistic's *P*-value. United States ecoregions were represented as Fescue Belt (FB), Forested Mountains (FM), High Plains (HP), and Upper Midwest & Northeast (UN).
