# Supplementary material for: Genome-wide association analyses identify genotype-by-environment interactions of growth traits in Simmental cattle
Source: Sci Rep. 2021 Jun 25;11:13335. doi: 10.1038/s41598-021-92455-x (PMC8233360; doi:10.1038/s41598-021-92455-x)
Supplement: Supplementary file 2 — Supplementary Information 2. [file 41598_2021_92455_MOESM2_ESM.docx]

**Genome-wide association analyses identify genotype-by-environment interactions of growth traits in Simmental cattle**

Camila U. Braz, Troy N. Rowan, Robert D. Schnabel, Jared E. Decker

Additional file 2

Supplementary Tables

**Table S1.** Summary statistics for growth traits and environmental variables across the US ecoregions.

| Traits/Env. Variables | SE | HP | FM | FB | UN | DA | Total |
| --- | --- | --- | --- | --- | --- | --- | --- |
| Birth Weight (kg) |  |  |  |  |  |  |  |
| N | 1151 | 3271 | 2397 | 3924 | 2173 | 511 | 13427 |
| Mean | 35.5 | 39.9 | 38.7 | 37.1 | 38.9 | 37.2 | 38.2 |
| SD | 4.4 | 4.9 | 4.8 | 4.4 | 4.5 | 5.2 | 4.8 |
| Weaning Weight (kg) |  |  |  |  |  |  |  |
| N | 873 | 3283 | 2329 | 3025 | 1883 | 454 | 11847 |
| Mean | 303.6 | 313.0 | 287.1 | 296.8 | 315.7 | 289.5 | 302.6 |
| SD | 42.2 | 44.0 | 38.6 | 46.2 | 43.3 | 42.0 | 44.6 |
| Yearling Weight (kg) |  |  |  |  |  |  |  |
| N | 533 | 2525 | 1999 | 1926 | 1293 | 270 | 8546 |
| Mean | 491.5 | 551.4 | 512.0 | 484.9 | 542.5 | 486.0 | 520.1 |
| SD | 78.2 | 81.1 | 71.5 | 89.7 | 81.5 | 71.6 | 85.1 |
| Multivariate |  |  |  |  |  |  |  |
| N | 533 | 2307 | 1992 | 1917 | 1278 | 267 | 8294 |
| Elevation (m) |  |  |  |  |  |  |  |
| Mean | 170.2 | 681.8 | 1587.0 | 341.6 | 372.0 | 914.8 | 747.5 |
| SD | 89.9 | 215.1 | 307.5 | 147.5 | 65.9 | 496.7 | 517.2 |
| Precipitation (ml) |  |  |  |  |  |  |  |
| Mean | 1289.8 | 420.7 | 390.6 | 946 | 749.2 | 497.6 | 643.8 |
| SD | 188.1 | 118.4 | 90.3 | 155.3 | 111.7 | 150.3 | 329.5 |
| Mean Temp. (^o^C) |  |  |  |  |  |  |  |
| Mean | 17.1 | 7.2 | 6.8 | 12.3 | 7.3 | 14.1 | 9.9 |
| SD | 1.6 | 1.8 | 1.4 | 1.3 | 1.4 | 3.9 | 3.8 |
| Min. Temp. (^o^C) |  |  |  |  |  |  |  |
| Mean | 11.1 | 0.6 | -0.8 | 6.1 | 1.8 | 7.4 | 3.3 |
| SD | 1.8 | 1.6 | 1.4 | 1.2 | 1.4 | 4.9 | 4.0 |
| Max. Temp. (^o^C) |  |  |  |  |  |  |  |
| Mean | 23.7 | 14.2 | 14.5 | 18.3 | 13.3 | 22.8 | 16.4 |
| SD | 1.6 | 2.1 | 1.8 | 1.5 | 1.5 | 3.1 | 3.7 |
| Mean Dew point Temp. (^o^C) |  |  |  |  |  |  |  |
| Mean | 11.5 | 0.2 | -2.2 | 6.3 | 2.5 | 5.8 | 3.0 |
| SD | 1.7 | 1.5 | 1.0 | 1.2 | 1.9 | 5.2 | 4.3 |
| Min. Vapor Pres. Deficit (hPs) |  |  |  |  |  |  |  |
| Mean | 1.0 | 1.3 | 1.4 | 1.1 | 0.9 | 1.7 | 1.2 |
| SD | 0.5 | 0.4 | 0.3 | 0.2 | 0.2 | 0.6 | 0.4 |
| Max. Vapor Pres. Deficit (hPs) |  |  |  |  |  |  |  |
| Mean | 17.0 | 13.0 | 13.7 | 13.2 | 10.0 | 21.0 | 13.3 |
| SD | 1.7 | 2.1 | 1.8 | 1.6 | 1.1 | 2.6 | 2.9 |

Number of animals (N), mean (mean) and standard deviation (SD) of the traits used in the univariate (Birth Weight, Weaning Weight, and Yearling Weight) and in the multivariate analysis, along with the environmental variables (Env. Variables) such as elevation, precipitation, mean temperature, minimum (Min) temperature, maximum (Max) temperature, mean dew point temperature, minimum (Min) vapor pressure deficit and maximum (Max) vapor pressure deficit. This information was provided using the full dataset (Total) and by ecoregion, which were named as Southeast (SE), High Plains (HP), Forested Mountains (FM), Fescue Belt (FB), Upper Midwest & Northeast (UN), Desert & Arid Plains (DA).

Table S2. False discovery rates for the nominal significant threshold (*P*-value = 1e-5) before (FDR_B_) and after (FDR_A_) adjustment of the *P*-values for genomic control inflation factor (λ_GC_) for each genome-wide association analysis (GWAA).

| Model | Birth Weight | | | Weaning Weight | | | Yearling Weight | | | Multivariate | | |
| --- | --- | --- | --- | --- | --- | --- | --- | --- | --- | --- | --- | --- |
|  | FDR_B_ | λ_GC_ | FDR_A_ | FDR_B_ | λ_GC_ | FDR_A_ | FDR_B_ | λ_GC_ | FDR_A_ | FDR_B_ | λ_GC_ | FDR_A_ |
|  | GWAA | | | | | | | | | | | |
|  | 0.007 | 0.924 | 0.007 | 0.014 | 0.963 | 0.014 | 0.010 | 0.950 | 0.010 | 0.007 | 0.947 | 0.007 |
|  | vGWAA | | | | | | | | | | | |
| MA | 0.507 | 0.992 | 0.507 | 1.418 | 1.000 | 1.418 | 0.442 | 1.000 | 0.442 | 1.177 | 0.992 | 1.177 |
| MADE | 0.888 | 0.997 | 0.888 | 7.091 | 1.000 | 7.091 | 0.393 | 1.000 | 0.393 | 1.177 | 0.998 | 1.177 |
|  | Continuous GxE GWAA | | | | | | | | | | | |
| MTemp | 0.012 | 1.591* | 0.129 | 7.091 | 0.964 | 7.091 | 1.767 | 0.953 | 1.767 | 0.102 | 1.130* | 0.294 |
| MinTemp | 0.010 | 1.714* | 0.129 | 1.182 | 0.945 | 1.182 | - | 0.957 | - | 0.080 | 1.191* | 0.252 |
| MaxTemp | 0.017 | 1.494* | 0.145 | 7.091 | 0.910 | 7.091 | 1.767 | 0.972 | 1.767 | 0.307 | 1.098* | 0.784 |
| MDpTemp | 0.008 | 1.778* | 0.113 | 1.773 | 0.963 | 1.773 | 7.068 | 0.957 | 7.068 | 0.075 | 1.227* | 0.282 |
| Elev | 0.050 | 1.368* | 1.368 | 0.253 | 1.097* | 0.473 | 0.471 | 1.122* | 0.707 | 0.141 | 1.402* | - |
| Precip | 0.021 | 1.394* | 0.092 | 1.773 | 0.984 | 1.773 | 7.068 | 0.999 | 1.773 | 0.588 | 1.212* | 3.530 |
| MinVPD | 0.047 | 1.579* | 3.551 | 3.545 | 1.081* | -0.98 | 7.068 | 0.985 | 7.068 | 0.706 | 1.122* | - |
| MaxVPD | 0.032 | 1.541* | 1.420 | 0.709 | 0.944 | 0.709 | 7.068 | 1.035* | 0.709 | 0.415 | 1.196* | 1.177 |
|  | Discrete GxE GWAA | | | | | | | | | | | |
| DA | 0.057 | 1.507* | 0.546 | 0.886 | 1.038* | 2.364 | 1.178 | 1.168* | - | - | - | - |
| SE | 0.132 | 1.262* | 3.551 | - | 0.874 | - | 3.534 | 0.950 | 3.534 | 1.009 | 1.016* | 1.009 |
| HP | 0.178 | 1.219* | 2.367 | 0.443 | 1.084* | 0.591 | 1.767 | 1.081* | 7.068 | 0.307 | 1.238* | 0.543 |
| FM | 0.079 | 1.222* | 0.338 | 0.229 | 1.171 | 0.417 | 0.144 | 1.218* | 0.883 | 0.048 | 1.543* | 7.059 |
| FB | 0.151 | 1.063* | 0.222 | 7.091 | 0.991 | 7.091 | 7.068 | 0.916 | 7.068 | 0.642 | 1.110* | 1.765 |
| UN | 0.296 | 1.253* | 3.551 | - | 0.865 | - | 7.068 | 1.035* | 7.068 | 0.706 | 1.144* | 3.530 |

vGWAA = variance-heterogeneity GWAA; GxE GWAA = genotype-by-environment interaction GWAA; MA = model removing only additive effects from the residuals; MADE = model removing additive, dominance, and epstasis effects from the residuals; Mtemp = mean temperature; MinTemp = minimum temperature; MaxTemp = maximum temperature; MdpTemp = mean dew point temperature; Elev = elevation; Precip = precipitation; MinVPD = minimum vapor pressure deficit; MaxVPD = maximum vapor pressure deficit; DA = Desert & Arid Plains; SE = Southeast; HP = High Plains; FM = Forested Mountains; FB = Fescue Belt; UN = Upper Midwest & Northeast. *GxE GWAA with λ_GC_ > 1, therefore the *P-values* were adjusted for λ_GC_.

**Table S3.** Function enrichment analysis (FDR ≤ 10%) for candidate genotype-by-environment interaction genes.

| Source | Term name | Term id | *P*-value_adj_ | N | GxE Genes* |
| --- | --- | --- | --- | --- | --- |
| GO:MF | Transmitter-gated ion channel activity | GO:0022824 | 1.8E-02 | 6 | *GABRB3^YW_MV^, GABRA4^BW_FB^, GABRB1^BW_FB^, GRIN2D^WW_EL^, GRIK2^BW_EL, BW_FM^, GRID2^BW_DT, BWmT^* |
| GO:MF | Transmitter-gated channel activity | GO:0022835 | 1.8E-02 | 6 | *GABRB3^YW_MV^, GABRA4^BW_FB^, GABRB1^BW_FB^, GRIN2D^WW_EL^, GRIK2^BW_EL, BW_FM^, GRID2^BW_DT, BWmT^* |
| GO:MF | Extracellular ligand-gated ion channel activity | GO:0005230 | 4.6E-02 | 6 | *GABRB3,^YW_MV^ GABRA4^BW_FB^, GABRB1^BW_FB^, GRIN2D^WW_EL^, GRIK2^WW_EL, BW_FM^, GRID2^BW_DT, BWmT^* |
| GO:MF | GABA-gated chloride ion channel activity | GO:0022851 | 5.8E-02 | 3 | *GABRB3,^YW_MV^ GABRA4^BW_FB^, GABRB1^BW_FB^* |
| GO:MF | Ligand-gated ion channel activity | GO:0015276 | 6.9E-02 | 7 | *GABRB3,^YW_MV^ GABRA4^BW_FB^, GABRB1^BW_FB^, GRIN2D^WW_EL^, GRIK2^WW_EL, BW_FM^, GRID2^BW_DT, BWmT^,* *KCNJ14**^WW_EL^* |
| GO:MF | Ligand-gated channel activity | GO:0022834 | 6.9E-02 | 7 | *GABRB3^YW_MV^, GABRA4^BW_FB^, GABRB1^BW_FB^, GRIN2D^WW_EL^, GRIK2^WW_EL, BW_FM^, GRID2^BW_DT, BWmT^,* *KCNJ14^WW_EL^* |
| GO:MF | Ionotropic glutamate receptor activity | GO:0004970 | 8.7E-02 | 3 | *GRIN2D^WW_EL^, GRIK2^WW_EL, BW_FM^ , GRID2^BW_DT, BWmT^* |
| GO:MF | GABA-A receptor activity | GO:0004890 | 8.7E-02 | 3 | *GABRB3^YW_MV^, GABRA4^BW_FB^, GABRB1^BW_FB^* |
| GO:MF | Ligand-gated anion channel activity | GO:0099095 | 8.7E-02 | 3 | *GABRB3^YW_MV^, GABRA4^BW_FB^, GABRB1^BW_FB^* |
| GO:MF | Transmitter-gated ion channel activity involved in regulation of postsynaptic membrane potential | GO:1904315 | 8.7E-02 | 4 | *GABRB3^YW_MV^, GABRA4^BW_FB^, GRIK2^WW_EL, BW_FM^, GRID2^BW_DT, BWmT^* |
| GO:MF | GABA receptor activity | GO:0016917 | 8.9E-02 | 3 | *GABRB3^YW_MV^, GABRA4^BW_FB^, GABRB1^BW_FB^* |
| GO:MF | Thioredoxin peroxidase activity | GO:0008379 | 8.9E-02 | 2 | *PRDX5^BW_DA^, PRDX3^YW_PR, YW_DT^* |
| GO:MF | Neurotransmitter receptor activity involved in regulation of postsynaptic membrane potential | GO:0099529 | 8.9E-02 | 4 | *GABRB3^YW_MV^, GABRA4^BW_FB^, GRIK2^BW_EL, BW_FM^, GRID2^BW_DT, BWmT^* |
| GO:MF | Neurotransmitter receptor activity | GO:0030594 | 8.9E-02 | 6 | *GABRB3^YW_MV^, GABRA4^BW_FB^, GABRB1^BW_FB^, GRIN2D^WW_EL^, GRIK2^BW_EL, BW_FM^, GRID2^BW_DT, BWmT^* |
| GO:CC | Intermediate filament | GO:0005882 | 6.1E-04 | 11 | *KRT15^M_FB^, KRT35^M_FB^, KRT31^M_FB^, KRT32^M_FB^, KRT33A^M_FB^, KRT34^M_FB^, KRT36^M_FB^,*  *ENSBTAG00000030519^M_FB^,* *ENSBTAG00000050581^M_FB^,*  *ENSBTAG00000053139^M_FB^* |
| GO:CC | Intermediate filament cytoskeleton | GO:0045111 | 1.7E-03 | 11 | *KRT15^M_FB^, KRT35^M_FB^, KRT31^M_FB^, KRT32^M_FB^, KRT33A^M_FB^, KRT34^M_FB^, KRT36^M_FB^,*  *ENSBTAG00000030519^M_FB^,* *ENSBTAG00000050581^M_FB^,*  *ENSBTAG00000053139^M_FB^* |
| GO:CC | GABA-A receptor complex | GO:1902711 | 9.5E-02 | 3 | *GABRB3^YW_MV^, GABRA4^BW_FB^, GABRB1^BW_FB^* |
| GO:CC | GABA receptor complex | GO:1902710 | 9.5E-02 | 3 | *GABRB3^YW_MV^, GABRA4^BW_FB^, GABRB1^BW_FB^* |
| KEGG | Estrogen signaling pathway | KEGG:04915 | 5.2E-07 | 13 | *AKT2^M_MV^, KRT15^M_FB^, KRT35^M_FB^, KRT31^M_FB^, KRT32^M_FB^, MMP9, KRT33A^M_FB^, KRT34^M_FB^, KRT36^M_FB^,*  *ENSBTAG00000030519^M_FB^,* *ENSBTAG00000050581^M_FB^,*  *ENSBTAG00000053139^M_FB^* |
| KEGG | Staphylococcus aureus infection | KEGG:05150 | 1.8E-06 | 11 | *KRT15^M_FB^, KRT35^M_FB^, KRT31^M_FB^, KRT32^M_FB^, KRT33A^M_FB^, KRT34^M_FB^, KRT36^M_FB^,*  *ENSBTAG00000030519^M_FB^,* *ENSBTAG00000050581^M_FB^,*  *ENSBTAG00000053139^M_FB^* |
| KEGG | Metabolism of xenobiotics by cytochrome P450 | KEGG:00980 | 4.3E-05 | 8 | *HPGDS^BW_FM, M_FM^, SULT2A1^WW_EL^,* *ENSBTAG00000039971^WW_EL^,* *ENSBTAG00000048013^BW_DT, BW_UN, BW_MT, BW_T, BW_mT, M_UN^,* *ENSBTAG00000051713^BW_DT, BW_UN, BW_MT, BW_T, BW_mT, M_UN^,* *ENSBTAG00000053068^BW_DT, BW_UN, BW_MT, BW_T, BW_mT, M_UN^,* *ENSBTAG00000054846^BW_DT, BW_UN, BW_MT, BW_T, BW_mT, M_UN^,*  *ENSBTAG00000055078 ^BW_UN, M_UN^* |
| KEGG | Chemical carcinogenesis | KEGG:05204 | 1.1E-04 | 8 | *HPGDS^BW_FM, M_FM^, SULT2A1^WW_EL^,* *ENSBTAG00000039971^WW_EL^,* *ENSBTAG00000048013^BW_DT, BW_UN, BW_MT, BW_T, BW_mT, M_UN^,* *ENSBTAG00000051713^BW_DT, BW_UN, BW_MT, BW_T, BW_mT, M_UN^,* *ENSBTAG00000053068^BW_DT, BW_UN, BW_MT, BW_T, BW_mT, M_UN^,* *ENSBTAG00000054846^BW_DT, BW_UN, BW_MT, BW_T, BW_mT, M_UN^,*  *ENSBTAG00000055078 ^BW_UN, M_UN^* |
| KEGG | Ascorbate and aldarate metabolism | KEGG:00053 | 1.9E-04 | 5 | *ENSBTAG00000051713^BW_DT, BW_UN, BW_MT, BW_T, BW_mT, M_UN^,* *ENSBTAG00000053068^BW_DT, BW_UN, BW_MT, BW_T, BW_mT, M_UN^,* *ENSBTAG00000054846^BW_DT, BW_UN, BW_MT, BW_T, BW_mT, M_UN^,*  *ENSBTAG00000055078 ^BW_UN, M_UN^* |
| KEGG | Pentose and glucuronate interconversions | KEGG:00040 | 6.6E-04 | 5 | *ENSBTAG00000051713^BW_DT, BW_UN, BW_MT, BW_T, BW_mT, M_UN^,* *ENSBTAG00000053068^BW_DT, BW_UN, BW_MT, BW_T, BW_mT, M_UN^,* *ENSBTAG00000054846^BW_DT, BW_UN, BW_MT, BW_T, BW_mT, M_UN^,*  *ENSBTAG00000055078 ^BW_UN, M_UN^* |
| KEGG | Drug metabolism - cytochrome P450 | KEGG:00982 | 1.5E-03 | 6 | *HPGDS^BW_FM, M_FM^,* *ENSBTAG00000039971^WW_EL^,* *ENSBTAG00000048013^BW_DT, BW_UN, BW_MT, BW_T, BW_mT, M_UN^,* *ENSBTAG00000051713^BW_DT, BW_UN, BW_MT, BW_T, BW_mT, M_UN^,* *ENSBTAG00000053068^BW_DT, BW_UN, BW_MT, BW_T, BW_mT, M_UN^,* *ENSBTAG00000054846^BW_DT, BW_UN, BW_MT, BW_T, BW_mT, M_UN^,*  *ENSBTAG00000055078 ^BW_UN, M_UN^* |
| KEGG | Porphyrin and chlorophyll metabolism | KEGG:00860 | 1.7E-03 | 5 | *ENSBTAG00000048013^BW_DT, BW_UN, BW_MT, BW_T, BW_mT, M_UN^,* *ENSBTAG00000051713^BW_DT, BW_UN, BW_MT, BW_T, BW_mT, M_UN^,* *ENSBTAG00000053068^BW_DT, BW_UN, BW_MT, BW_T, BW_mT, M_UN^,* *ENSBTAG00000054846^BW_DT, BW_UN, BW_MT, BW_T, BW_mT, M_UN^,*  *ENSBTAG00000055078 ^BW_UN, M_UN^* |
| KEGG | Bile secretion | KEGG:04976 | 2.0E-03 | 7 | *SULT2A1^WW_EL^,* *ENSBTAG00000039971^WW_EL^,* *ENSBTAG00000048013^BW_DT, BW_UN, BW_MT, BW_T, BW_mT, M_UN^,* *ENSBTAG00000051713^BW_DT, BW_UN, BW_MT, BW_T, BW_mT, M_UN^,* *ENSBTAG00000053068^BW_DT, BW_UN, BW_MT, BW_T, BW_mT, M_UN^,* *ENSBTAG00000054846^BW_DT, BW_UN, BW_MT, BW_T, BW_mT, M_UN^,*  *ENSBTAG00000055078 ^BW_UN, M_UN^* |
| KEGG | Retinol metabolism | KEGG:00830 | 2.0E-03 | 6 | *ALDH1A2^YW_EL^, ENSBTAG00000039971^WW_EL^,* *ENSBTAG00000048013^BW_DT, BW_UN, BW_MT, BW_T, BW_mT, M_UN^,* *ENSBTAG00000051713^BW_DT, BW_UN, BW_MT, BW_T, BW_mT, M_UN^,* *ENSBTAG00000053068^BW_DT, BW_UN, BW_MT, BW_T, BW_mT, M_UN^,* *ENSBTAG00000054846^BW_DT, BW_UN, BW_MT, BW_T, BW_mT, M_UN^,*  *ENSBTAG00000055078 ^BW_UN, M_UN^* |
| KEGG | Steroid hormone biosynthesis | KEGG:00140 | 2.6E-03 | 6 | *SULT2A1^WW_EL^,* *ENSBTAG00000039971^WW_EL^,* *ENSBTAG00000048013^BW_DT, BW_UN, BW_MT, BW_T, BW_mT, M_UN^,* *ENSBTAG00000051713^BW_DT, BW_UN, BW_MT, BW_T, BW_mT, M_UN^,* *ENSBTAG00000053068^BW_DT, BW_UN, BW_MT, BW_T, BW_mT, M_UN^,* *ENSBTAG00000054846^BW_DT, BW_UN, BW_MT, BW_T, BW_mT, M_UN^,*  *ENSBTAG00000055078 ^BW_UN, M_UN^* |
| KEGG | Nicotine addiction | KEGG:05033 | 1.3E-02 | 4 | *GABRB3^YW_MV^, GABRA4^BW_FB^, GABRB1^BW_FB^, GRIN2D^WW_EL^* |
| KEGG | Drug metabolism - other enzymes | KEGG:00983 | 2.2E-02 | 5 | *ENSBTAG00000048013^BW_DT, BW_UN, BW_MT, BW_T, BW_mT, M_UN^,* *ENSBTAG00000051713^BW_DT, BW_UN, BW_MT, BW_T, BW_mT, M_UN^,* *ENSBTAG00000053068^BW_DT, BW_UN, BW_MT, BW_T, BW_mT, M_UN^,* *ENSBTAG00000054846^BW_DT, BW_UN, BW_MT, BW_T, BW_mT, M_UN^,*  *ENSBTAG00000055078 ^BW_UN, M_UN^* |
| KEGG | Phospholipase D signaling pathway | KEGG:04072 | 2.2E-02 | 7 | *AKT2^M_MV^, CYTH2^WW_EL^, CYTH1^WW_MV^, PLA2G4B^BW_FM^, PLD1^BW_MT, BW_MV^, PLA2G4E^BW_FM^, GNA13^BW_MV^* |
| KEGG | Endocytosis | KEGG:04144 | 2.5E-02 | 9 | *CYTH2^WW_EL^, CYTH1^WW_MV^, EHD4^BW_FM^, GRK5^YW_DT, YW_PR^, PLD1^BW_MT, BW_MV^, SH3GL3^M_DT, M_mT^, BOLA-NC1^M_T^, EHD2^WW_EL^,* *ENSBTAG00000038619^M_T^* |
| KEGG | Morphine addiction | KEGG:05032 | 4.7E-02 | 5 | *PDE10A^WW_mT^, GRK5^YW_DT, YW_PR^, GABRB3^YW_MV^, GABRA4^BW_FB^, GABRB1^BW_FB^* |
| KEGG | VEGF signaling pathway | KEGG:04370 | 5.0E-02 | 4 | *AKT2^M_MV^, PLA2G4B^BW_FM^, BAD^BW_DA^, PLA2G4E^BW_FM^* |
| KEGG | Long-term depression | KEGG:04730 | 5.0E-02 | 4 | *PLA2G4B^BW_FM^, PLA2G4E^BW_FM^, GNA13^BW_MV^, GRID2^BW_DT, BWmT^* |
| KEGG | Viral myocarditis | KEGG:05416 | 8.8E-02 | 4 | *SGCB^BW_PR^, BOLA-NC1^M_T^, CD40^BW_HP^,* *ENSBTAG00000038619^M_T^* |
| KEGG | Glutamatergic synapse | KEGG:04724 | 8.8E-02 | 5 | *PLA2G4B^BW_FM^, PLD1^BW_MT, BW_MV^, PLA2G4E^BW_FM^, GRIN2D^WW_EL^, GRIK2^W_EL, BW_FM^* |
| REAC | Formation of the cornified envelope | REAC:R-BTA-6809371 | 7.6E-03 | 7 | *KRT15^M_FB^, KRT31^M_FB^, KRT32^M_FB^, KRT33A^M_FB^, KRT34^M_FB^, KRT36^M_FB^* |
| REAC | Keratinization | REAC:R-BTA-6805567 | 2.1E-02 | 7 | *KRT15^M_FB^, KRT31^M_FB^, KRT32^M_FB^, KRT33A^M_FB^, KRT34^M_FB^, KRT36^M_FB^* |

*Denotes the trait and environmental variable analyzed. BW = birth weight; WW = weaning weight; YW = yearling weight; M = mutivariate; T = mean temperature; mT = minimum temperature; MT = maximum temperature; DT = mean dew point temperature; EL = elevation; PR = precipitation; mV = minimum vapor pressure deficit; MV = maximum vapor pressure deficit; DA = Desert & Arid Plains; SE = Southeast; HP = High Plains; FM = Forested Mountains; FB = Fescue Belt; UN = Upper Midwest & Northeast.

**Table S4.** Biological processes possibly being affected by candidate genotype-by-environment interaction genes.

| GO name | GO term | N | GxE genes |
| --- | --- | --- | --- |
| Nitrogen compound metabolic process | GO:0006807 | 72 | *AKT2, AMZ2, AREG, BAD, BICRA, C1QTNF1, CACUL1, CALCR, CANT1, CD40, CNOT6L, CNTD2, CRX, DCAF16, EDEM2, EMSY, ENTPD8, ESRRA, EXD3, FKBP14, FKBP2, GLUD1, GRK5, GSDME, HSPA5, ITIH2, ITIH5, JMJD7, KLHL20, KMT2E, LAP3, LATS1, LMTK3, MACROD1, MAP3K10, MMP24, MMP9, MRPL1, NANOS1, NELFB, NOP53, NR3C2, NRARP, PCIF1, PCMT1, PDHA2, PPP6C, PRDM16, PRDX3, PRDX5, PSMC4, RBFOX3, RPP21, SCRN1, SCYL2, SHLD2, SLIT2, SNCAIP, SPATA18, SULT2B1, TP73, TRIM39, TRMT112, TRPC4AP, TRPT1, UBAC2, USP24, VEGFB, WIPI1, YTHDC1, ZNF114, ZNF335* |
| Response to stimulus | GO:0050896 | 65 | *ADGRB3, AKT2, ALDH1A2, AREG, ARHGEF16, ARHGEF28, BAD, C1QTNF1, CALCR, CCDC88B, CD40, CRX, CXCL13, CYTH1, CYTH2, EDEM2, ESRRA, FERMT3, GABRA4, GABRB1, GABRB3, GAPVD1, GNA13, GNL1, GRID2, GRIK2, GRIN2D, GRK5, GSDME, HSPA5, KCNK4, KDELR1, LATS1, LRP11, LRRC32, MACROD1, MAP3K10, MAPKBP1, MMP24, MMP9, NCOA5, NOP53, NR3C2, NRARP, NRXN1, PDE10A, PLD1, PRDM16, PRDX3, PRDX5, PROCR, RERGL, RPP21, SHLD2, SLC12A5, SLIT2, SPATA18, TENM3, TP73, TRIM39, UBAC2, ULBP21, VEGFB, VEGFC, WIPI1* |
| Protein metabolic process | GO:0019538 | 51 | *AKT2, AMZ2, AREG, BAD, C1QTNF1, CACUL1, CANT1, CD40, CNOT6L, CNTD2, DCAF16, EDEM2, FKBP14, FKBP2, GRK5, GSDME, HSPA5, ITIH2, ITIH5, JMJD7, KLHL20, KMT2E, LAP3, LATS1, LMTK3, MACROD1, MAP3K10, MMP24, MMP9, MRPL1, NANOS1, NOP53, PCIF1, PCMT1, PPP6C, PRDM16, PRDX3, PRDX5, PSMC4, SCRN1, SCYL2, SLIT2, SPATA18, TRIM39, TRPC4AP, TRPT1, UBAC2, USP24, VEGFB, WIPI1, ZNF335* |
| Signaling | GO:0023052 | 48 | *ADGRB3, AKT2, ALDH1A2, AREG, ARHGEF28, BAD, C1QTNF1, CALCR, CD40, CXCL13, CYTH1, CYTH2, ESRRA, FERMT3, GABRB1, GABRB3, GAPVD1, GLUD1, GRID2, GRIK2, GRIN2D, GRK5, GSDME, HSPA5, LATS1, LRRC32, MAP3K10, MAPKBP1, MMP9, NCOA5, NOP53, NR3C2, NRARP, NRXN1, NSMF, PDE10A, PLD1, PRDM16, RERGL, SLC12A5, SLIT2, SNCAIP, TENM3, TP73, TPRG1L, TRIM39, UBAC2, VEGFB* |
| Developmental process | GO:0032502 | 46 | *ADGRB3, AKT2, ALDH1A2, AREG, BAD, CALCR, CD40, CRX, EHD2, FERMT3, GABRA4, GNA13, GRID2, GRK5, GSDME, HSPA5, KDELR1, KMT2E, KRT36, LATS1, LRRC32, MMP24, MMP9, MTURN, NANOS1, NRARP, NRXN1, NSMF, OTOL1, PRDM16, PRDX3, PSMC4, PTPRZ1, SGCB, SHLD2, SLC12A5, SLIT2, SULT2B1, TENM3, TP73, TRPC4AP, VEGFB, VEGFC, WDR72, YTHDC1, ZNF335* |
| Anatomical structure development | GO:0048856 | 44 | *ADGRB3, AKT2, ALDH1A2, AREG, BAD, CALCR, CD40, CRX, FERMT3, GABRA4, GNA13, GRID2, GSDME, HSPA5, KDELR1, KMT2E, KRT36, LATS1, LRRC32, MMP24, MMP9, MTURN NANOS1, NRARP, NRXN1, NSMF, OTOL1, PRDM16, PRDX3, PSMC4, PTPRZ1, SGCB, SHLD2, SLC12A5, SLIT2, SULT2B1, TENM3, TP73, TRPC4AP, VEGFB, VEGFC, WDR72, YTHDC1, ZNF335* |
| Regulation of metabolic process | GO:0019222 | 44 | *AKT2, ALDH1A2, AREG, BAD, BICRA, C1QTNF1, CACUL1, CALCR, CD40, CNOT6L, CRX, EDEM2, EMSY, ESRRA, GSDME, HSPA5, ITIH2, ITIH5, KMT2E, LATS1, LRRC32, MAP3K10, MMP9, NANOS1, NELFB, NOP53, NR3C2, NRARP, PCIF1, PRDM16, PRDX3, PRDX5, PSMC4, SHLD2, SLIT2, TP73, TRIM39, TRMT112, TRPT1, UBAC2, VEGFB, YTHDC1, ZNF114, ZNF335* |
| Gene expression | GO:0010467 | 31 | *ALDH1A2, BICRA, C1QTNF1, CALCR, CD40, CNOT6L, CRX, EMSY, ESRRA, KMT2E, LRRC32, MAP3K10, MRPL1, NANOS1, NELFB, NOP53, NR3C2, NRARP, PCIF1, PRDM16, PRDX3, PRDX5, PSMC4, RBFOX3, RPP21, TP73, TRMT112, TRPT1, YTHDC1, ZNF114, ZNF335* |
| Nervous system development | GO:0007399 | 30 | *ADGRB3, DFFB, SLC12A5, PTPRZ1, GSDME, CDH22, SLIT2 HSPA5, GABRA4, NANOS1, AREG, GRID2, BAD, GABRB1, GRK5, GABRB3, TENM3, NRXN1, PRDM16, TP73, AKT2, GLUD1, GPRIN2, MMP24, ALDH1A2, RBFOX3, SCYL2, VEGFC, ARHGEF28, GPRIN3* |
| Response to stress | GO:0006950 | 27 | *BAD, C1QTNF1, CCDC88B, CD40, CXCL13, EDEM2, FERMT3, GNA13, GNL1, GRIK2, HSPA5, LRP11, MACROD1, MAP3K10, MAPKBP1, MMP9, NOP53, PRDX3, PRDX5, PROCR, SHLD2, SLC12A5, SPATA18, TP73, UBAC2, VEGFB, WIPI1* |
| Immune system process | GO:0006955 | 15 | *MTURN, CALCR, MMP9, NRARP, BAD, PRDX3, PTPRZ1, KDELR1, ZNF335, SHLD2, LRRC32, CCDC88B, KMT2E, ULBP21, CXCL13* |

**Table S5.** Proportion of genetic variance based on the additive genomic model (MA), and additive, dominance and epistasis genomic model (MADE) and their standard errors for each trait.

| Trait | MA | MADE | | |
| --- | --- | --- | --- | --- |
|  | ${\sigma_{A}^{2}}/{\sigma_{P}^{2}}$ | ${\sigma_{A}^{2}}/{\sigma_{P}^{2}}$ | ${\sigma_{D}^{2}}/{\sigma_{P}^{2}}$ | ${\sigma_{Ep}^{2}}/{\sigma_{P}^{2}}$ |
| Birth weight | 0.37 ± 0.01 | 0.33 ± 0.01 | 0.00 ± 0.01 | 0.25 ± 0.03 |
| Weaning weight | 0.30 ± 0.01 | 0.28 ± 0.02 | 0.02 ± 0.02 | 0.12 ± 0.04 |
| Yearling weight | 0.40 ± 0.01 | 0.40 ± 0.02 | 0.05 ± 0.02 | 0.11 ± 0.05 |

${\sigma_{A}^{2}}/{\sigma_{P}^{2}}$**,** ${\sigma_{D}^{2}}/{\sigma_{P}^{2}}$**,** ${\sigma_{Ep}^{2}}/{\sigma_{P}^{2}}$ are proportion of additive, dominance, and epistasis variances to total phenotypic variance, respectively.

**Table S6.** Significant vQTL (*P* < 1e-5) for growth traits when using residuals adjusted for additive effects (MA) or for additive, dominance and epistasis effects (MADE).

| Trait | SNP | BTA | Position | Freq | MA | | MADE | | Genes |
| --- | --- | --- | --- | --- | --- | --- | --- | --- | --- |
|  |  |  |  |  | Effect (kg) | *P*-value | Effect (kg) | *P*-value |  |
| BW | *rs110323698* | 10 | 22,312,496 | 0.127 | 0.026 ± 1.6e-04 | 7.07e-06 | - | - | *DAD1, ABHD4* |
|  | *rs137529355* | 13 | 35,496,312 | 0.278 | - | - | -0.020 ± 9.3e-05 | 6.04e-06 | *LYZL1* |
|  | *rs133623280* | 14 | 17,005,811 | 0.458 | -0.019 ± 7.9e-05 | 2.94e-06 | -0.018 ± 7.9e-05 | 6.69e-06 | *ZHX2* |
|  | *rs109136768* | 14 | 17,007,761 | 0.418 | 0.018 ± 8.6e-05 | 9.60e-06 | - | - |  |
|  | *rs43026996* | 14 | 17,166,118 | 0.344 | 0.022 ± 1.1e-04 | 3.51e-06 | 0.021 ± 1.1e-04 | 6.67e-06 |  |
|  | *rs43186199* | 14 | 17,183,088 | 0.347 | 0.022 ± 1.1e-04 | 3.10e-06 | 0.021 ± 1.1e-04 |  |  |
|  | *rs42796317* | 14 | 17,195,903 | 0.356 | 0.021 ± 1.1e-04 | 9.31e-06 | - | - |  |
|  | *rs109515648* | 14 | 23,128,784 | 0.376 | 0.024 ± 1.4e-04 | 6.04e-06 | - | - | *TMEM68, TGS1, LYN, RPS20, MOS, PLAG1, CHCHD7, SDR16C5* |
|  | *rs137303549* | 14 | 23,298,062 | 0.388 | 0.024 ± 1.4e-04 | 6.48e-06 | - | - |  |
|  | *rs134215421* | 14 | 23,329,375 | 0.385 | 0.025 ± 1.4e-04 | 2.49e-06 | - | - |  |
|  | *rs109815800* | 14 | 23,338,890 | 0.384 | 0.024 ± 1.4e-04 | 5.38e-06 | - | - |  |
|  | *rs41843599* | 17 | 57,685,345 | 0.144 | - | - | 0.026 ± 1.7e-04 | 6.42e-06 | *NOS1, KSR2, FBXO21, TESC, FBXW8* |
|  | *rs41843601* | 17 | 57,685,935 | 0.146 | 0.026 ± 1.6e-04 | 6.60e-06 | 0.026 ± 1.6e-04 | 5.62e-06 |  |
|  | *rs109209365* | 17 | 57,699,496 | 0.141 | - | - | 0.026 ± 1.7e-04 | 8.79e-06 |  |
|  | *rs41847363* | 17 | 57,975,119 | 0.146 | 0.026 ± 1.6e-04 | 7.91e-06 | 0.026 ± 1.6e-04 | 9.43e-06 |  |
|  | *rs134957935* | 21 | 51,465,082 | 0.188 | -0.022 ± 1.2e-04 | 9.21e-06 | - | - | *LRFN5* |
| WW | *rs109967828* | 3 | 106,206,442 | 0.169 | 0.025 ± 1.6e-04 | 9.18e-06 | - | - | *MFSD2A, MYCL, TRIT1, BMP8B* |
|  | *rs43657596* | 11 | 7,293,721 | 0.133 | 0.027 ± 1.8e-04 | 7.17e-06 | - | - | *IL18RAP, SLC9A4, SLC9A2* |
|  | *rs134174267* | 11 | 7,304,992 | 0.101 | 0.035 ± 2.4e-04 | 5.08e-07 | 0.033 ± 2.4e-04 | 1.78e-06 |  |
|  | *rs109287854* | 18 | 37,372,422 | 0.219 | 0.022 ± 1.2e-04 | 8.65e-06 | - | - | *-* |
|  | *rs110803856* | 27 | 40,193,292 | 0.209 | 0.023 ± 1.3e-04 | 8.88e-06 | - | - | *TOP2B, RARB* |
| YW | *rs135719485^£^* | 5 | 115,845,266 | 0.336 | -0.025 ± 1.3e-04 | 1.93e-06 | -0.026 ± 1.3e-04 | 4.19e-07 | *RIBC2, FBLN1, SMC1B* |
|  | *rs133130907^¥^* | 14 | 74,597,382 | 0.092 | 0.044 ± 3.1e-04 | 7.42e-08 | 0.044 ± 3.2e-04 | 4.08e-08 | *MMP16* |
|  | *rs41802941* | 16 | 36,184,280 | 0.037 | 0.057 ± 7.7e-04 | 6.73e-06 | - | - | *XCL2, XCL1, DPT* |
|  | *rs41802957* | 16 | 36,200,777 | 0.050 | 0.050 ± 5.8e-04 | 4.27e-06 | 0.050 ± 5.8e-04 | 4.86e-06 |  |
|  | *rs137574197* | 16 | 36,231,107 | 0.050 | 0.051 ± 5.9e-04 | 3.36e-06 | 0.051 ± 5.9e-04 | 4.02e-06 |  |
|  | *rs109835794* | 16 | 36,242,339 | 0.052 | 0.050 ± 5.6e-04 | 3.09e-06 | 0.050 ± 5.7e-04 | 3.46e-06 |  |
|  | *rs134575841* | 17 | 44,811,665 | 0.432 | - | - | 0.022 ± 1.2e-04 | 8.59e-06 | *GALNT9* |
|  | *rs111011858* | 17 | 70,514,528 | 0.010 | 0.103 ± 2.5e-03 | 5.18e-06 | 0.107 ± 2.5e-03 | 2.53e-06 | *YWHAH, SLC5A1, DEPDC5, SLC5A4* |
|  | *rs110473197* | 17 | 70,523,020 | 0.010 | 0.103 ± 2.5e-03 | 5.08e-06 | 0.107 ± 2.5e-03 | 2.50e-06 |  |
|  | *rs109010785* | 17 | 70,527,754 | 0.010 | 0.103 ± 2.5e-03 | 5.08e-06 | 0.107 ± 2.5e-03 | 2.50e-06 |  |
|  | *rs108982594* | 17 | 70,540,863 | 0.010 | 0.103 ± 2.5e-03 | 5.08e-06 | 0.107 ± 2.5e-03 | 2.50e-06 |  |
|  | *rs378352090* | 18 | 62,939,763 | 0.149 | - | - | 0.034 ± 2.8e-04 | 7.85e-06 | *TTYH1, LENG8, LENG9, LAIR1, CDC42EP5, RPS9, TSEN34, MBOAT7, TMC4, LENG1, CNOT3, PRPF31, TFPT, NDUFA3, OSCAR, TARM1* |
|  | *rs383417308* | 18 | 63,229,878 | 0.155 | - | - | 0.034 ± 2.8e-04 | 7.42e-06 |  |
|  | *rs433152283* | 18 | 63,230,047 | 0.155 | - | - | 0.034 ± 2.8e-04 | 6.22e-06 |  |
|  | *rs43727853* | 27 | 33,796,650 | 0.460 | -0.023 ± 1.3e-04 | 7.00e-06 | - | - | *TACC1* |
|  | *rs110205571* | 27 | 34,027,900 | 0.281 | 0.024 ± 1.4e-04 | 5.33e-06 | - | - | *PLEKHA2* |
|  | *rs110909628* | 27 | 35,715,673 | 0.223 | - | - | 0.026 ± 1.6e-04 | 8.82e-06 | *ZMAT4* |
|  | *rs137312252^*^* | 27 | 35,899,682 | 0.097 | 0.041 ± 3.2e-04 | 3.42e-07 | 0.042 ± 3.2e-04 | 2.68e-07 | *ZMAT4* |
|  | *rs135719206^*^* | 27 | 35,902,981 | 0.092 | 0.042 ± 3.4e-04 | 6.24e-07 | 0.042 ± 3.4e-04 | 4.34e-07 |  |
|  | *rs134473626^*^* | 27 | 35,906,532 | 0.095 | 0.042 ± 3.3e-04 | 2.64e-07 | 0.042 ± 3.3e-04 | 2.51e-07 |  |
|  | *rs136265144* | 29 | 48,072,601 | 0.254 | -0.027 ± 1.5e-04 | 1.90e-06 | -0.027 ± 1.5e-04 | 1.40e-06 | *SHANK2* |
| MV | *rs135719485^£^* | 5 | 115,845,266 | 0.334 | - | - | - | 8.47e-06 | *RIBC2, FBLN1, SMC1B* |
|  | *rs109142386* | 8 | 4,822,842 | 0.258 | - | - | - | 7.78e-06 | *GALNTL6* |
|  | *rs133130907^¥^* | 14 | 74,597,382 | 0.091 | - | 9.98e-07 | - | 8.47e-06 | *MMP16* |
|  | *rs137312252^*^* | 27 | 35,899,682 | 0.098 | - | 2.04e-06 | - | 3.77e-07 | *ZMAT4* |
|  | *rs135719206^*^* | 27 | 35,902,981 | 0.092 | - | 1.06e-06 | - | 3.45e-06 |  |
|  | *rs134473626^*^* | 27 | 35,906,532 | 0.096 | - | 1.81e-06 | - | 3.10e-06 |  |
|  | *rs135379559* | 29 | 39,441,489 | 0.099 | - | 2.83e-06 | - | - | *PAG6, PAG11, LOC528815* |

Birth weight (BW), Weaning weight (WW), Yearling weight (YW), multivariate (MV), allele frequency (Freq), allele substitution effect (Effect).
